# Supplementary material for: A bipartite iron-dependent transcriptional regulation of the tryptophan salvage pathway in Chlamydia trachomatis
Source: eLife. 2019 Apr 2;8:e42295. doi: 10.7554/eLife.42295 (PMC6504234; doi:10.7554/eLife.42295)
Supplement: Source code 1. [file elife-42295-code1.pdf]

```
> setwd("~/Documents/Carabeo Lab/R Code/3'RACE")
> library(ggplot2)
> library(plyr)
> race<-read.csv("pRACE_v2.csv")

#calculate weight mean
> rmean<-ddply(race, "Product", summarize, r.mean=weighted.mean(Position, Count))
> rmean
  Product   r.mean
1  aFirst 511665.4
2 bSecond 511811.1
3  cThird 511889.1
4 dFourth 511985.5

#plot data
> ggplot(race, aes(x=Position, fill=Product)) + geom_vline(data = rmean,
aes(xintercept=r.mean, color=Product), linetype="dashed", size=1) +
geom_histogram(aes(y=..density..), binwidth=20, color="black", alpha=0.25) +
geom_density(alpha=0.75) + theme_classic(base_size=24) +
scale_y_continuous(expand=c(0,0), limits=c(0,0.05)) + scale_x_continuous(limits =
c(511600, 512050), breaks = c(511600, 511700, 511800, 511900, 512000))
Printed 10.5x5.25
```
