# Supplementary material for: A bipartite iron-dependent transcriptional regulation of the tryptophan salvage pathway in Chlamydia trachomatis
Source: eLife. 2019 Apr 2;8:e42295. doi: 10.7554/eLife.42295 (PMC6504234; doi:10.7554/eLife.42295)
Supplement: Source code 2. [file elife-42295-code2.pdf]

```
> trpb<-read.csv("finaltrpb.csv")

#One-Way ANOVA
> anova(lm(Expression~Condition, data=trpb))
Analysis of Variance Table

Response: Expression
          Df Sum Sq Mean Sq F value    Pr(>F)
Condition  2 5.1456 2.57278  29.645 0.0007761 ***
Residuals  6 0.5207 0.08679
---
Signif. codes:  0 ‘***’ 0.001 ‘**’ 0.01 ‘*’ 0.05 ‘.’ 0.1 ‘ ’ 1

#Pair-wise t-test with bonferronis correction for multiple comparisons
> pairwise.t.test(trpb$Expression, trpb$Condition, p.adj="bonferroni")

          Pairwise comparisons using t tests with pooled SD

data:  trpb$Expression and trpb$Condition

     12UT    18UT
18UT 0.44870 -
6BPD 0.00383 0.00098
```
