# Supplementary material for: A bipartite iron-dependent transcriptional regulation of the tryptophan salvage pathway in Chlamydia trachomatis
Source: eLife. 2019 Apr 2;8:e42295. doi: 10.7554/eLife.42295 (PMC6504234; doi:10.7554/eLife.42295)
Supplement: Source code 3. [file elife-42295-code3.pdf]

```
> y<-read.csv("trpBA_3.csV")

#Student's t-test with Welch's correction for unequal variance
> t.test(Miller~Condition, data=y)

	Welch Two Sample t-test

data:  Miller by Condition
t = 7.6208, df = 2.2332, p-value = 0.01219
alternative hypothesis: true difference in means is not equal to 0
95 percent confidence interval:
 166.8512 516.5333
sample estimates:
  mean in group pET151-EV mean in group pET151-YtgR
                 589.8532                  248.1609
```
