# Supplementary material for: A bipartite iron-dependent transcriptional regulation of the tryptophan salvage pathway in Chlamydia trachomatis
Source: eLife. 2019 Apr 2;8:e42295. doi: 10.7554/eLife.42295 (PMC6504234; doi:10.7554/eLife.42295)
Supplement: Supplementary file 3. [file elife-42295-supp3.docx]

May 10 2017

>B1

NNNNNNTNNNNNNGGGNAGCAGTGGTATCAACGCAGAGTACATGGGTTAACACTATAATTTTATGGAGAAAAGATGTTCAAACATAAACATCCTTTTGGGGGAGCGTTCCTTCCCGAAGAACTATTAGCCCCTATACAGAATCTAAAAGCGGAATGGGAGATTCTCAAAACTCAGCAAAGTTTTTTATCTGAACTAGATTGTATTTTGAAAAACTATGCGGGGAGACAAACTCCTCTGACTGAAGTTAAGAATTTTGCTCGAGCTATTGATGGCCCTAGAGTATTTCTTAAACGCGAAGATCTTTTGCATACAGGAGCACATAAACTGAATAATGCTCTTGGTCAGTGTTTGCTTGCTAAATATCTTGGGAAAACACGTTTTGTAGCTGAAACAGGTGCGGGACAACATGGAGTAGCAACAGCAACAGCGTGTGCTTATCTAGGATTAGATTGTGTAGTATACATGGGAGCAAAAGATGTGGAACGACAGAAACCAAATGTAGAGAAAATGCGCTTTTTAGGTGCTGAAGTCGTTTCTGTAACAAAAGGATCTTGTGGACTCAAAGATGCAGTTAATCAAGCTCTACAAGATTGGGCAACAACACACTCATTTACTCACTATTGCTTAGGATCGGCCTTAGGACCTTTACCTTATCCCGATATCGTTCGATTTTTTCAGTCTGTTATAAGCGCTGAAGTGAAAGAGCAAATCCATGCAGTTGCAGGAAGAGATCCTGATATTCTGATTGCATGTATCGGAGGTGGCTCCAACGCTATTGGATTTTTCCATCATTTTATCCCGAATCCAAAAGTCCAATTAATTGGAGTGGAAGGGGGGAGGACTGGGCATTTCTTCAGGAAAACATGCAGCACGTTTTGCAACAGGGCGACCTGGAGTATTCCACGGATTTTATTCGTATCTTCTTCAAGATGACGATGGACAAGTATTACAAACTCACTCCATTTCCGCTGGATTAGATTATCCTTCAGTTGGGNCAGATCATGCCGAAATGCATGAGTCAGGACGAGCCTTNANNNTTAANCCACCGATGAAANGNNTNNNNAGCTAANTNGGCNANNNNTGGNCNNNNNNNTTNCCNGGGNNAATTGTATCCNNNNNNTTCCANNNNNNCGAGCNGGNNNNANNNTNANNNNNGGGGGNCNNNNNNNNNNAACNNNTNNNNNNNNNNNANNNNNNTTNNNNNGNANNNNNNNNNNNNNNNNNNNNNNNNNNNNNNNNNNNNNNNNNNNTNNGNNNNNNNNNNNNNNNNNNNNNNNNNNNNNCNNNNNNNNNNNANNNNNNNNNNNCCNNANNNNNNNNNCNNGGNNNGGNNANNNNNN

>B2

NNNNNNTNCCNNNNGGGNAGCAGTGGTATCAACGCAGAGTACATGGGACATAAACATCCTTTTGGGGGAGCGTTCCTTCCCGAAGAACTATTAGCCCCTATACAGAATCTAAAAGCGGAATGGGAGATTCTCAAAACTCAGCAAAGTTTTTTATCTGAACTAGATTGTATTTTGAAAAACTATGCGGGGAGACAAACTCCTCTGACTGAAGTTAAGAATTTTGCTCGAGCTATTGATGGCCCTAGAGTATTTCTTAAACGCGAAGATCTTTTGCATACAGGAGCACATAAACTGAATAATGCTCTTGGTCAGTGTTTGCTTGCTAAATATCTTGGGAAAACACGTGTTGTAGCTGAAACAGGTGCGGGACAACATGGAGTAGCAACAGCAACAGCGTGTGCTTATCTAGGATTAGATTGTGTAGTATACATGGGAGCAAAAGATGTGGAACGACAGAAACCAAATGTAGAGAAAATGCGCTTTTTAGGTGCTGAAGTCGTTTCTGTAACAAAAGGATCTTGTGGACTCAAAGATGCAGTTAATCAAGCTCTACAAGATTGGGCAACAACACACTCATTTACTCACTATTGCTTAGGATCGGCCTTAGGACCTTTACCTTATCCCGATATCGTTCGATTTTTTCAGTCTGTTATAAGCGCTGAAGTGAAAGAGCAGATCCATGCAGTTGCAGGAAGAGATCCTGATATTCTGATTGCATGTATCGGAGGTGGCTCCAACGCTATTGGATTTTTCCATCATTTTATCCCGAATCCAAAAGTCCAATTAATTGGAGTGGAAGGGGGGAGGACTGGGCATTTCTTCAGGAAAACATGCAGCACGTTTTGCAACAGGGCGACCTGCAGTATTCCACGGATTTTATTCGTATCTTCTTCAAGATGACGATGGACAAGTATTACAANCTCACTCCATTTCCGCTGGATTAGATTATCCTTCAGTTGGGCCNNATCATGCCGAANTGCATGAGTCAGGACTAGCCTTTNNTCATTAGCCACCGATGAAGAGGNNTACGANNTAGCTTNNCGGATCNNGGTNATACNTGTTCCNNNNGNAATCGTNNNNNNCNNATTNANAACTANAAGCCGAAGCTAANNNNANNCNGNNNNNCNANNNNNNNCNANNNNNNANNGNTNNNNNNNNNCCNTTNCCAANNGGAACNNNNNNNCNNNNNNNNANNCCCNNNCCNGNNNNNNNTTNNNNNNGGCNNNNNNNNNNNANNNNNNNNNCGCCGNNNNNANNNNNNNN

>B3

NNNNNNNTNNNNNNGGNNAGCAGTGGTATCAACGCAGAGTGCATGGGTGTCGCCAGTTTTTTCTATGATTTTAACACTATAATTTTATGGAGAAAAGATGTTCAAACATAAACATCCTTTTGGGGGAGCGTTCCTTCCCGAAGAACTATTAGCCCCTATACAGAATCTAAAAGCGGAATGGGAGATTCTCAAAACTCAGCAAAGTTTTTTATCTGAACTAGATTGTATTTTGAAAAACTATGCGGGGAGACAAACTCCTCTGACTGAAGTTAAGAATTTTGCTCGAGCTATTGATGGCCCTAGAGTATTTCTTAAACGCGAAGATCTTTTGCATACAGGAGCACATAAACTGAATAATGCTCTTGGTCAGTGTTTGCTTGCTAAATATCTTGGGAAAACACGTGTTGTAGCTGAAACAGGTGCGGGACAACATGGAGTAGCAACAGCAACAGCGTGTGCTTATCTAGGATTAGATTGTGTAGTATACATGGGAGCAAAAGATGTGGAACGACAGAAACCAAATGTAGAGAAAATGCGCTTTTTAGGTGCTGAAGTCGTTTCTGTAACAAAAGGATCTTGTGGACTCAAAGATGCAGTTAATCAAGCTCTACAAGATTGGGCAACAACACACTCATTTACTCACTATTGCTTAGGATCGGCCTTAGGACCTTTACCTTATCCCGATATCGTTCGATTTTTTCAGTCTGTTATAAGCGCTGAAGTGAAAGAGCAAATCCATGCAGTTGCAGGAAGAGATCCTGATATTCTGATTGCATGTATCGGAGGTGGCTCCAACGCTATTGGATTTTTCCATCATTTTATCCCGAATCCANAAGTCCATTAATTGGATGGAANNGGGGNNNAAGGACTGGGCNTTTNCTTCAGGAAAACATGCAGCACGTTTTGCAACAGGGCGACCTGGAGTATTCCACGGATTTTATTCGTATCTTCTTCAAGATGACGATGGACAAGTATTACAAACTCACTCCATTTCCGCTGGATTAGATTATCCTTCAGTTGGGCCAGATCATGCCGAAATGCATGAGTCAGGACAAGCCTTNNNNCATNAGCNNCCGATGAAGAGNNTTACGAGNNANNTTGNNNNNNNNTGGNNNNNNNNNNNNNNNGGNNAANTTNNNNCCNNTCCAATTCCANNNNNTNNNNCNNNNNNNAANNNNAANNNNGGGNNNNNNNNNNNANGCAACCNNTNNTNNNTNNNNNNNNNNNNNNNCNNNNNANNNNNNNNNNNNNNNNNNNNNNANNCNGNNNNNNNNNNNNNNNNNNNNNNNCNNAANNNNNNNNNNNNNNNNNNNNNNNNNNNNNNNNNNNNNNNNNNNNNNN

>B4

NNNNNNNTNNNNNNGGGCAGCAGTGGTATCAACGCAGAGTGCATGGGAACATAAACATCCTTTTGGGGGAGCGTTCCTTCCCGAAGAACTATTAGCCCCTATACAGAATCTAAAAGCGGAATGGGAGATTCTCAAAACTCAGCAAAGTTTTTTATCTGAACTAGATTGTATTTTGAAAAACTATGCGGGGAGACAAACTCCTCTGACTGAAGTTAAGAATTTTGCTCGAGCTATTGATGGCCCTAGAGTATTTCTTAAACGCGAAGATCTTTTGCATACAGGAGCACATAAACTGAATAATGCTCTTGGTCAGTGTTTGCTTGCTAAATATCTTGGGAAAACACGTGTTGTAGCTGAAACAGGTGCGGGACAACATGGAGTAGCAACAGCAACAGCGTGTGCTTATCTAGGATTAGATTGTGTAGTATACATGGGAGCAAAAGATGTGGAACGACAGAAACCAAATGTAGAGAAAATGCGCTTTTTAGGTGCTGAAGTCGTTTCTGTAACAAAAGGATCTTGTGGACTCAAAGATGCAGTTAATCAAGCTCTACAAGATTGGGCAACAACACACTCATTTACTCACTATTGCTTAGGATCGGCCTTAGGACCTTTACCTTATCCCGATATCGTTCGATTTTTTCAGTCTGTTATAAGCGCTGAAGTGAAAGAGCAAATCCATGCAGTTGCAGGAAGAGATCCTGATATTCTGATTGCATGTATCGGAGGTGGCTCCAACGCTATTGGATTTTTCCATCATTTTATCCCGAATCCAAAAGTCCAATTAATTGGAGTGGAAGGGGGGAGGACTGGGCATTTCTTCAGGAAAACATGCAGCACGTTTTGCAACAGGGCGACCTGGAGTATTCCACGGATTTTATTCGTATCTTCTTCAAGATGACGATGGACAAGTATTACAAACTCACTCCATTTCCGCTGGATTAGATTATCCTTCAGTTGGNCCAGATCATGCCGAAATGCATGAGTCAGGACGAGCCTTNATACTTAGCCACCGATGAAGNGNNGTACCAGCTAACTTGGCNNNTCATGGTCTAGCTGTTTCCTGGGGNAATTGTATCCNNCNAATTCNACAACTANNNCCNGANGCTAANNNNAAGCNNGGGGTGCCAANNNNNNNNNNNNNNNNNTTNNNNGNNNNNGNCCNTTNNNNNNGGAACTNNNNNNNNTGTTNNNNNNNNNNNNNNNNNGNNNGNNNNNNNNGTTNGGNNNNCNNNNNNNNNANNNNNNNNNGNNNNNNNNNNANNNNNNNNNNNNNNNNNNNNNCNNNNNNNNNNNNCNNNCNGAGCNNNCNNNNNNGNGTNAACNNNNNNNNNNNNNNNNTNNNNNNNAGNN

>B5

NNNNNNTNNNNNNGGNNAGCAGTGGTATCAACGCAGAGTACATGGGCTATGATTTTAACACTATAATTTTATGGAGAAAAGATGTTCAAACATAAACATCCTTTTGGGGGAGCGTTCCTTCCCGAAGAACTATTAGCCCCTATACAGAATCTAAAAGCGGAATGGGAGATTCTCAAAACTCAGCAAAGTTTTTTATCTGAACTAGATTGTATTTTGAAAAACTATGCGGGGAGACAAACTCCTCTGACTGAAGTTAAGAATTTTGCTCGAGCTATTGATGGCCCTAGAGTATTTCTTAAACGCGAAGATCTTTTGCATACAGGAGCACATAAACTGAATAATGCTCTTGGTCAGTGTTTGCTTGCTAAATATCTTGGGAAAACACGTGTTGTAGCTGAAACAGGTGCGGGACAACATGGAGTAGCAACAGCAACAGCGTGTGCTTATCTAGGATTAGATTGTGTAGTATACATGGGAGCAAAAGATGTGGAACGACAGAAACCAAATGTAGAGAAAATGCGCTTTTTAGGTGCTGAAGTCGTTTCTGTAACAAAAGGATCTTGTGGACTCAAAGATGCAGTTAATCAAGCTCTACAAGATTGGGCAACAACACACTCATTTACTCACTATTGCTTAGGATCGGCCTTAGGACCTTTACCTTATCCCGATATCGTTCGATTTTTTCAGTCTGTTATAAGCGCTGAAGTGAAAGAGCAAATCCATGCAGTTGCAGGAAGAGATCCTGATATTCTGATTGCATGTATCGGAGGTGGCTCCAACGCTATTGGATTTTTCCATCATTTTATCCCGAATCCAAAAGTCCAATTAATTGGAGTGAAAGGGGGGAGGACTGGGCATTTCTTCAGGAAAACATGCAGCACGTTTTGCAACAGGGCGACCTGGAGTATTCCACGGATTTTATTCNTATCTTCTTCAAGATGACGATGGNNNAGTATTACAACTCCTCCATTTCCGCTGGATANNTTATCCTTCAGTTGGGCNNNTCATGCCGAANTGNNTGAGTCAGNNNNNNNTTTNAACTTAGCCNNCCGATGAAANGGNTTACANNTAANTNGGNNNNNNNNGGNCAANNGNTTNCCNGGGNAAATNNNNCCNNNNANTTCCNNNANNNNNNCCNGGANNNANNNTGAANCCNGGGGGNCNNNNNNNNNNNNNANNTNNNNNNGNNNNNNNCCNTTNNNNNGNANNNNNNNNNNNNANNNNNNNNNANNNNGNNNGNNNNNNNNTNGGNNNNNNNNNNNNNNNNNNNCCNNNNGNNNNNNNN

>B6

NNNNNNCTNNNNNNGGNNNGCAGTGGTATCAACGCAGAGTACATGGGTAACACTATAATTTTATGGAGAAAAGATGTTCAAACATAAACATCCTTTTGGGGGAGCGTTCCTTCCCGAAGAACTATTAGCCCCTATACAGAATCTAAAAGCGGAATGGGAGATTCTCAAAACTCAGCAAAGTTTTTTATCTGAACTAGATTGTATTTTGAAAAACTATGCGGGGAGACAAACTCCTCTGACTGAAGTTAAGAATTTTGCTCGAGCTATTGATGGCCCTAGAGTATTTCTTAAACGCGAAGATCTTTTGCATACAGGAGCACATAAACTGAATAATGCTCTTGGTCAGTGTTTGCTTGCTAAATATCTTGGGAAAACACGTGTTGTAGCTGAAACAGGTGCGGGACAACATGGAGTAGCAACAGCAACAGCGTGTGCTTATCTAGGATTAGATTGTGTAGTATACATGGGAGCAAAAGATGTGGAACGACAGAAACCAAATGTAGAGAAAATGCGCTTTTTAGGTGCTGAAGTCGTTTCTGTAACAAAAGGATCTTGTGGACTCAAAGATGCAGTTAATCAAGCTCTACAAGATTGGGCAACAACACACTCATTTACTCACTATTGCTTAGGATCGGCCTTAGGACCTTTACCTTATCCCGATATCGTTCGATTTTTTCAGTCTGTTATAAGCGCTGAAGTGAAAGAGCAAATCCATGCAGTTGCAGGAAGAGATCCTGATATTCTGATTGCATGTATCGGAGGTGGCTCCAACGCTATTGGATTTTTCCATCATTTTATCCCGAATCCAAAAGTCCAATTAATTGGAGTGGAAGGGGGGAGGACTGGGCATTTCTTCAGGAAAACATGCAGCACGTTTTGCAACAGGGCGACCTGGAGTATTCCACGGATTTTATTCGTATCTTCTTCAAGATGACGATGGACAAGTATTACAAACTCACTCCATTTCCGCTGGNNTAGATTATCCTTCAGTTGGGCNNATCATGCCGAAATGCATGAGTCAGGACGAGCCTTNNTNCTTNNCCNNCCGATGAANNGNNTTNNAGNNAANTTGGNNANNNNNGGNNNNNNNNNTTNCCNGGGGNAATTGTNNCCNNNNNTTCCNNNCNACGNCCNNNNNNNAANGTGAANCCNGGGGGNCNANNNNNNNNNNNNNNNNNNNGGNNNNNNNNCNNTTNNNNGNANNNNTGNNNNNANNNNANNNNNNNNNNNCGNANNNNNNNNNGGNNNNNNNNNNNNNNGNCNNNNGNNNNNNNNNNNNNNNNNNNNNNNNNNNNNNNANNNNNNNANNNNNNNNNNNNGNNNNNNNNNNNNNTNNNNNNNNNNNNNNNNNNNNNNNNNNNNNNNNNNNNNNNNNNNNNNN

>B7

NNNNNNNTANNNNNGGGCAGCAGTGGTATCAACGCAGAGTACATGGTCTATGATTTTAACACTATAATTTTATGGAGAAAAGATGTTCAAACATAAACATCCTTTTGGGGGAGCGTTCCTTCCCGAAGAACTATTAGCCCCTATACAGAATCTAAAAGCGGAATGGGAGATTCTCAAAACTCAGCAAAGTTTTTTATCTGAACTAGATTGTATTTTGAAAAACTATGCGGGGAGACAAACTCCTCTGACTGAAGTTAAGAATTTTGCTCGAGCTATTGATGGCCCTAGAGTATTTCTTAAACGCGAAGATCTTTTGCATACAGGAGCACATAAACTGAATAATGCTCTTGGTCAGTGTTTGCTTGCTAAATATCTTGGGAAAACACGTGTTGTAGCTGAAACAGGTGCGGGACAACATGGAGTAGCAACAGCAACAGCGTGTGCTTATCTAGGATTAGATTGTGTAGTATACATGGGAGCAAAAGATGTGGAACGACAGAAACCAAATGTAGAGAAAATGCGCTTTTTAGGTGCTGAAGTCGTTTCTGTAACAAAAGGATCTTGTGGACTCAAAGATGCAGTTAATCAAGCTCTACAAGATTGGGCAACAACACACTCATTTACTCACTATTGCTTAGGATCGGCCTTAGGACCTTTACCTTATCCCGATATCGTTCGATTTTTTCAGTCTGTTATAAGCGCTGAAGTGAAAGAGCAAATCCATGCAGTTGCAGGAAGAGATCCTGATATTCTGATTGCATGTATTCGGAGGTGGCTCCAACGCNNNTTGGATTTTTCCATCATTTTATCCCGAATCCAAAGTCCATTAATTGGAGTGGAANNGGGGNAAGGNNTNGGGCATTTCTTCAGGAAAACATGCAGCACGTTTTGCAACAGGGCGACCTGGAGTATTCCACGGATTTTATTCGTATCTTCTTCAAGATGACGATGGACAAGTATTACAANCTCACTCCATTTCCGCTGGATTAGATTATCCTTCAGTTGGGCCAGATCATGCCGAAATGCATGAGTCAGGACGAGCCTTNATACTTAGCCACCGATGAAGANNNNTNNGAGCTAAGCTTGGCGANTNATGGNCNANGNTGTNCCNGNTNNAATTGTNNCNNNNNNAATTCCANNNNCNNNNNNNNGNNNNNNAANNNNAANGNNGGGGGNNNNANNNNNNCNNNNNNNNNNNNNNTNNNNNNNNNNNNTTNNNNNGGNANNTGNNNNNNNNNNNNNNNNNNNNNNNNNNNNNGNNTNNNNNNGNNNNNNNNCNNNNNNCNNNNNNTTNGGNANNNNNNNNNGNNNNNNNNNNNNNGNNNNNNNGNCNNNNNNNNNNNANNNNNNNNNNNNNNNNNNNNTNNNTNNNNNNNNNNNNN

>B8

NNNNNNNTNNNNNNGGGCAGCAGTGGTATCAACGCAGAGTACATGGGTAACACTATAATTTTATGGAGAAAAGATGTTCAAACATAAACATCCTTTTGGGGGAGCGTTCCTTCCCGAAGAACTATTAGCCCCTATACAGAATCTAAAAGCGGAATGGGAGATTCTCAAAACTCAGCAAAGTTTTTTATCTGAACTAGATTGTATTTTGAAAAACTATGCGGGGAGACAAACTCCTCTGACTGAAGTTAAGAATTTTGCTCGAGCTATTGATGGCCCTAGAGTATTTCTTAAACGCGAAGATCTTTTGCATACAGGAGCACATAAACTGAATAATGCTCTTGGTCAGTGTTTGCTTGCTAAATATCTTGGGAAAACACGTGTTGTAGCTGAAACAGGTGCGGGACAACATGGAGTAGCAACAGCAACAGCGTGTGCTTATCTAGGATTAGATTGTGTAGTATACATGGGAGCAAAAGATGTGGAACGACAGAAACCAAATGTAGAGAAAATGCGCTTTTTAGGTGCTGAAGTCGTTTCTGTAACAAAAGGATCTTGTGGACTCAAAGATGCAGTTAATCAAGCTCTACAAGATTGGGCAACAACACACTCATTTACTCACTATTGCTTAGGATCGGCCTTAGGACCTTTACCTTATCCCGATATCGTTCGATTTTTTCAGTCTGTTATAAGCGCTGAAGTGAAAGAGCAAATCCATGCAGTTGCAGGAAGAGATCCTGATATTCTGATTGCATGTATCGGAGGTGGCTCCAACGCTATTGGATTTTTCCATCATTTTATCCCGAATCCAAAAGTCCAATTAATTGGAGTGGAAGGGGGAGGACTGGGCATTTCTTCAGGAAAACATGCAGCACGTTTTNNAACNGGGNGACCTGGAGTATTCCCNGGATNNNATTCNNNNNTTCTNNAAGATGACGATGGNNNNNNNNNNNANNNNNNNCNN

>M1

NNNNNNNNNNNNCTNNNGGGNAGCAGTGGTATCAACGCAGAGTACCTGGGTACAGCGTGCCTGGCTTTCTTTTGAAAGCTGGCGCTTATCTACTTGGCGATAGGCCTAATTAAGAAGCCTTTTATTTGATTAAGAGATGTTCTTATAGAAGTAAGAGCGTCTTTTTTGCGCAGGATTATTCTGTCGCCAGTTTTTTCTATGATTTTAACACTATAATTTTATGGAGAAAAGATGTTCAAACATAAACATCCTTTTGGGGGAGCGTTCCTTCCCGAAGAACTATTAGCCCCTATACAGAATCTAAAAGCGGAATGGGAGATTCTCAAAACTCAGCAAAGTTTTTTATCTGAACTAGATTGTATTTTGAAAAACTATGCGGGGAGACAAACTCCTCTGACTGAAGTTAAGAATTTTGCTCGAGCTATTGATGGCCCTAGAGTATTTCTTAAACGCGAAGATCTTTTGCATACAGGAGCACATAAACTGAATAATGCTCTTGGTCAGTGTTTGCTTGCTAAATATCTTGGGAAAACACGTGTTGTAGCTGAAACAGGTGCGGGACAACATGGAGTAGCAACAGCAACAGCGTGTGCTTATCTAGGATTAGATTGTGTAGTATACATGGGAGCAAAAGATGTGGAACGACAGAAACCAAATGTAGAGAAAATGCGCTTTTTAGGTGCTGAAGTCGTTTCTGTAACAAAAGGATCTTGTGGACTCAAAGATGCAGTTAATCAAGCTCTACAAGATTGGGCAACAACACACTCATTTACTCACTATTGCTTAGGATCGGCCTTAGGACCTTTACCTTATCCCGATATCGTTCGATTTTTTCAGTCTGTTATAAGCGCTGAAGTGAAAGAGCAAATCCATGCAGTTGCAGGAAGAGATCCTGATATTCTGATTGCATGTATCGGAGGTGGCTCCAACGCTATTGGATTNTTCCATCATTTNATCCCGAATCCAAAAGTCCANTNAATTGGAGTGGAAGGGGNAGGANGGGNCNTTNNNNACGGAANNNNNNNNNNNGGNNNNAANNNGNCNNCTNGNNNNNTNCGCGGNATTATNNNNNNNNNTNNGNNGCGC

>M2

NNNNNNCTNNNNNNGGGCAGCAGTGGTATCAACGCAGAGTAACATGGGCTACTTGGCGATAGGCCTAATTAAGAAGCCTTTTATTTGATTAAGAGATGTTCTTATAGAAGTAAGAGCGTCTTTTTTGCGCAGGATTATTCTGTCGCCAGTTTTTTCTATGATTTTAACACTATAATTTTATGGAGAAAAGATGTTCAAACATAAACATCCTTTTGGGGGAGCGTTCCTTCCCGAAGAACTATTAGCCCCTATACAGAATCTAAAAGCGGAATGGGAGATTCTCAAAACTCAGCAAAGTTTTTTATCTGAACTAGATTGTATTTTGAAAAACTATGCGGGGAGACAAACTCCTCTGACTGAAGTTAAGAATTTTGCTCGAGCTATTGATGGCCCTAGAGTATTTCTTAAACGCGAAGATCTTTTGCATACAGGAGCACATAAACTGAATAATGCTCTTGGTCAGTGTTTGCTTGCTAAATATCTTGGGAAAACACGTGTTGTAGCTGAAACAGGTGCGGGACAACATGGAGTAGCAACAGCAACAGCGTGTGCTTATCTAGGATTAGATTGTGTAGTATACATGGGAGCAAAAGATGTGGAACGACAGAAACCAAATGTAGAGAAAATGCGCTTTTTAGGTGCTGAAGTCGTTTCTGTAACAAAAGGATCTTGTGGACTCAAAGATGCAGTTAATCAAGCTCTACAAGATTGGGCAACAACACACTCATTTACTCACTATTGCTTAGGATCGGCCTTAGGACCTTTACCTTATCCCGATATCGTTCGATTTTTTTCAGTCTGTTATAAGCGCTGAAGTGAAAGAGCAAATCCATGCAGTTGCAGGAAGAGATCCTGATATTCTGATTGCATGTATCGGAGGTGGCTCCAACGCTATTGGATTTTTCCATCATTTTATCCCGAATCCAAAAGTCCNTNNANTTGGAGTGGAAGGGGGAGGACTGNNNATTTCTTNNGNAAACATGNNTCACGTGTTNNANCGGNTNNACNGGNNNNNNCCNGGNATTNATTNNNNNNNNNNNNNNNNNNNNNGNANNNNNNNNANNNNNN

>M3

NNNNANNNCCNNNNGGNNAGCAGTGGTATCAACGCAGAGTACATGGGCTATGATTTTAACACTATAATTTTATGGAGAAAAGATGTTCAAACATAAACATCCTTTTGGGGGAGCGTTCCTTCCCGAAGAACTATTAGCCCCTATACAGAATCTAAAAGCGGAATGGGAGATTCTCAAAACTCAGCAAAGTTTTTTATCTGAACTAGATTGTATTTTGAAAAACTATGCGGGGAGACAAACTCCTCTGACTGAAGTTAAGAATTTTGCTCGAGCTATTGATGGCCCTAGAGTATTTCTTAAACGCGAAGATCTTTTGCATACAGGAGCACATAAACTGAATAATGCTCTTGGTCAGTGTTTGCTTGCTAAATATCTTGGGAAAACACGTGTTGTAGCTGAAACAGGTGCGGGACAACATGGAGTAGCAACAGCAACAGCGTGTGCTTATCTAGGATTAGATTGTGTAGTATACATGGGAGCAAAAGATGTGGAACGACAGAAACCAAATGTAGAGAAAATGCGCTTTTTAGGTGCTGAAGTCGTTTCTGTAACAAAAGGATCTTGTGGACTCAAAGATGCAGTTAATCAAGCTCTACAAGATTGGGCAACAACACACTCATTTACTCACTATTGCTTAGGATCGGCCTTAGGACCTTTACCTTATCCCGATATCGTTCGATTTTTTCAGTCTGTTATAAGCGCTGAAGTGAAAGAGCAAATCCATGCAGTTGCAGGAAGAGATCCTGATATTCTGATTGCATGTATCGGAGGTGGCTCCAACGCTATTGGATTTTTCCATCATTTTATCCCGAATCCAAAAGTCCAATTAATTGGAGTGGAAAGGGGGAGGACTGGGCATTTCTTCAGGAAAACATGCAGCACGTTTTGCAACAGGGCGACCTGGAGTATTCCACGGATTTTATTCATATCTTCTTCAAGATGACGATGGACAAGTATTACAAACTCACTCCATTTCCGCTGGATTNNATTATCCTTCAGTTGGNCCAGATCATGCCGAANTGCATGAGTCAGGACGNGCNTTNNNNNATTANCNNCNGATGAAGNGGNTNACGANNNANNTNGGCGAACCNGGGCTANNNGTTCNNGGGNAAATTGTNNCNNNNCNATTCCACNNNNNNNNCCGNNNNNANGNNNAANCNGGNNNNNNNGNNNAGCANNNNNTNANNNNNNNNNNNNNNNNNTNNNNGNNNAANCNGNNNNNNNTNANNNNNNNNNCNNNNNNNNN

>M4

NNNNNNTNNNNNNGGGNAGCAGTGGTATCAACGCAGGGCACATGGGCTACTTGGCGATAGGCCTAATTAAGAAGCCTTTTATTTGATTAAGAGATGTTCTTATAGAAGTAAGAGCGTCTTTTTTGCGCAGGATTATTCTGTCGCCAGTTTTTTCTATGATTTTAACACTATAATTTTATGGAGAAAAGATGTTCAAACATAAACATCCTTTTGGGGGAGCGTTCCTTCCCGAAGAACTATTAGCCCCTATACAGAATCTAAAAGCGGAATGGGAGATTCTCAAAACTCAGCAAAGTTTTTTATCTGAACTAGATTGTATTTTGAAAAACTATGCGGGGAGACAAACTCCTCTGACTGAAGTTAAGAATTTTGCTCGAGCTATTGATGGCCCTAGAGTATTTCTTAAACGCGAAGATCTTTTGCATACAGGAGCACATAAACTGAATAATGCTCTTGGTCAGTGTTTGCTTGCTAAATATCTTGGGAAAACACGTGTTGTAGCTGAAACAGGTGCGGGACAACATGGAGTAGCAACAGCAACAGCGTGTGCTTATCTAGGATTAGATTGTGTAGTATACATGGGAGCAAAAGATGTGGAACGACAGAAACCAAATGTANAGAAAATGCGCTTTTTAAGTGCTGAAGTCATTTCTGTAACNANAGGATCTTGTGNANTANNTGATGCAGNTAATCNNNNTCNACAAGATTNNNCATCCNNNNNCTCNNTTACNCANNNTGNATTAGGATCCGACCTTAGANCNTNTACCCNNCCTTANCCGTTCNANNNANTNAATNTNATTATATNNNCAGANNNAANAAANNNATTTACTTCGNNANNNNGNANANANNTCATNNNACANGAANNCTGNATNNTGCACGGAACTAATGCNGNTCNANTNTGATGAGNTNNACNNTAATTCCNNNNNNCCATNNNCNNNNNNNNTANNNNANNNCNNCTNNNCNGNNNNNATNNN

>M5

NNNNNNNTNNNNNNGGGCAGCAGTGGTATCAACGCAGAGTACATGGGATAATTTTATGGAGAAAAGATGTTCAAACATAAACATCCTTTTGGGGGAGCGTTCCTTCCCGAAGAACTATTAGCCCCTATACAGAATCTAAAAGCGGAATGGGAGATTCTCAAAACTCAGCAAAGTTTTTTATCTGAACTAGATTGTATTTTGAAAAACTATGCGGGGAGACAAACTCCTCTGACTGAAGTTAAGAATTTTGCTCGAGCTATTGATGGCCCTAGAGTATTTCTTAAACGCGAAGATCTTTTGCATACAGGAGCACATAAACTGAATAATGCTCTTGGTCAGTGTTTGCTTGCTAAATATCTTGGGAAAACACGTGTTGTAGCTGAAACAGGTGCGGGACAACATGGAGTAGCAACAGCAACAGCGTGTGCTTATCTAGGATTAGATTGTGTAGTATACATGGGAGCAAAAGATGTGGAACGACAGAAACCAAATGTAGAGAAAATGCGCTTTTTAGGTGCTGAAGTCGTTTCTGTAACAAAAGGATCTTGTGGACTCAAAGATGCAGTTAATCAAGCTCTACAAGATTGGGCAACAACACACTCATTTACTCACTATTGCTTAGGATCGGCCTTAGGACCTTTACCTTATCCCGATATCGTTCGATTTTTTCAGTCTGTTATAAGCGCTGAAGTGAAAGAGCAAATCCATGCAGTTGCAGGAAGAGATCCTGATATTCTGATTGCATGTATCGGAGGTGGCTCCAACGCTATTGGATTTTTCCATCATTTTATCCCGAATCCAAAAGTCCAATTAATTGGAGTNANNGGGGGGAGGACTGGGCATTTCTTCAGGAAAACATGCAGCACGTTTTGCAACAGGGCGACCTGGAGTATTCCACGGATTTTATTCGTATCTTCTTCAAGATGACGATGGACAAGTATTACAAACTCACTCCATTTCCNNNTGGATTAGATTATCCTTCAGTTGGGNNAGATCATGCCGAAATGCATGAGTCAGGACGAGCCTTTTNNCTTNNCCNACCGATGAAGNGNNTTCNAGCTAANNNGNNNANTCNTGGNCNAGNNNNTTNCCNNNGNNAATTGTATCNNNNNANTTCCANNNNNACNNNNNNNNNNNAANNNNNANNCCNGGGNTGCNAANNNNNNNNNNNNNNNNNNNNTNNNNNNNNNNNTTNNNNNGNANNNGNNNNNNNNNNANNNNNNNNNNCNNNNNNGNNGNNNNNNNNGNNNNNNNNCNCNNNNNNNNNNNNNNNNNANNNNNNANNNNNNCNANNNNNNNANNNGNGGNNNNNNNNNNNNNTNNNNNNNNNNNNNNNNNNNNNNNNNNTNNNNNNNNNNNNNNNNNNNNNNNNNNNNNN

>M8

NNNNNNNTNNNNNNGGGCAGCAGTGGTATCAACGCAGAGACATGGGTTAACACTATAATTTTATGGAGAAAAGATGTTCAAACATAAACATCCTTTTGGGGGAGCGTTCCTTCCCGAAGAACTATTAGCCCCTATACAGAATCTAAAAGCGGAATGGGAGATTCTCAAAACTCAGCAAAGTTTTTTATCTGAACTAGATTGTATTTTGAAAAACTATGCGGGGAGACAAACTCCTCTGACTGAAGTTAAGAATTTTGCTCGAGCTATTGATGGCCCTAGAGTATTTCTTAAACGCGAAGATCTTTTGCATACAGGAGCACATAAACTGAATAATGCTCTTGGTCAGTGTTTGCTTGCTAAATATCTTGGGAAAACACGTGTTGTAGCTGAAACAGGTGCGGGACAACATGGAGTAGCAACAGCAACAGCGTGTGCTTATCTAGGATTAGATTGTGTAGTATACATGGGAGCAAGAGATGTGGAACGACAGAAACCAAATGTAGAGAAAATGCGCTTTTTAGGTGCTGAAGTCGTTTCTGTAACAAAAGGATCTTGTGGACTCAAAGATGCAGTTAATCAAGCTCTACAAGATTGGGCAACAACACACTCATTTACTCACTATTGCTTAGGATCGGCCTTAGGACCTTTACCTTATCCCGATATCATTCGATTTTTTCAGTCTGTTATAAGCGCTGAAGTGAAAGAGCAAATCCATGCAGTTGCAGGAAGAGATCCTGATATTCTGATTGCATGTATCGGAGGTGGCTCCAACGCTATTGGATTTTTCCATCATTTTATCCCGAATCCAAAAGTCCAATTAATTGGAGTGGAAGGGGGAGGACTGGGCATTTCTTCAGGAAAACATGCAGCACGTTTTGCANNATGGCGACCTGGAGTATTCNACGGATTTNATTCGANTCTTCTCAAGATGNNGATGGACNNNNTNNCCAACNCNCNCNATNNCCCCNGGATCAGNNNNCNNNNNNNNGNCTNGATCAGGCCNAATTNNNNNNTNNNNNNNNCNNNNNGNNNNNNNNNNNNNNNNNNNNNNNNTN

>T1

NNNNNNNNNNNNNGGGNNGCAGTGGTATCAACGCAGAGTACATGGGATTACAAAAAGGTGCGATATGAAAAATCAAGAGGAGTCTGGCTGGCAAGCTTTTCTGACATTATGCTCTAAAATGCAAAAAGAAAAGTTTTTACAAGACCTTTTTTCGCTGTTTTTGTCTTTTGGCGAACGTAAAGATGTCGCTTCTCGCTATCATATCATTCGAGCTCTTTTAGAAGGGGAGCTCACTCAAAGAGAGATAGCAGAGAAATACGGAGTCAGTATCGCACAAATTACCAGAGGATCTAATGCCCTTAAAGGATCAGATCCTCAATTTAAAGAGTTTTTACAAAAAGAGATCTGATCTTCTTTTGTAAAATACAAATAAGATTGAAAGTATTTGTATGCACGCGTTGTTAATGAACAAATATTCTGTTTTAGCAGTTTTGGTACGTAAGTATAGCTGCAGCATGCCATGCAAATCAGCTTTTCAAGCTGATTGCTTCCAAGATATTCAAAAATTCATCCTCTTACAGCGTGCCTGGCTTTCTTTTGAAAGCTGGCGCTTATCTACTTGGCGATAGGCCTAATTAAGAAGCCTTTTATTTGATTAAGAGATGTTCTTATAGAAGTAAGAGCGTCTTTTTTGCGCAGGATTATTCTGTCGCCAGTTTTTTCTATGATTTTAACACTATAATTTTATGGAGAAAAGATGTTCAAACATAAACATCCTTTTGGGGGAGCGTTCCTTCCCGAAGAACTATTAGCCCCTATACAGAATCTAAAAGCGGAATGGGAGATTCTCAAAACTCAGCAAAGTTTTTTATCTGAACTAGATTGTATTTTGAAAAACTATGCGGGGAGACAAACTCCTCTGACTGAAGTTAAGAATTTTGCTCGAGCTATTGATGGCCCTAGAGTATTTCTTAAACGCGAAGATCTTTTGCATACAGGAGCACATAAACTGANNAATGCTCTTNGGTCAGTGTTGNNNNNTAAATATCTTGGGAAAACACGTGTTGNNCTGAAACAGNNNNNNNNNNNNNGGATANAACAGCACNCTGGNNNNNNNNAGNNNNNGATTGGNNNNNNNCATGGGAGCAANGNNGGNACGNNNAANCCAATGNNNNANNNNNCNTTTTNAGGNCTGAATNNTNNNNNNNAAANGGNNNNGGNNNNAAANGNNTNNNNNNNNTCCAANNGGGNNNNNNNTTNNNNCNTTNNNNNNNNNNTNNNNNNNNNNCNNNNNNGNTTTNCNNNNNNNNNNNNNNNNNNNGNNNNGAANNNNNTNNNNNNNNNNNCCN

>T2

NNNNNNNTNNNNNNGGNNNGCAGTGGTATAACGCAGAGTACATGGGCTTACAAAAAGGTGCGATATGAAAAATCAAGAGGAGTCTGGCTGGCAAGCTTTTCTGACATTATGCTCTAAAATGCAAAAAGAAAAGTTTTTACAAGACCTTTTTTCGCTGTTTTTGTCTTTTGGCGAACGTAAAGATGTCGCTTCTCGCTATCATATCATTCGAGCTCTTTTAGAAGGGGAGCTCACTCAAAGAGAGATAGCAGAGAAATACGGAGTCAGTATCGCACAAATTACCAGAGGATCTAATGCCCTTAAAGGATCAGATCCTCAATTTAAAGAGTTTTTACAAAAAGAGATCTGATCTTCTTTTGTAAAATACAAATAAGATTGAAAGTATTTGTATGCACGCGTTGTTAATGAACAAATATTCTGTTTTAGCAGTTTTGGTACGTAAGTATAGCTGCAGCATGCCATGCAAATCAGCTTTTCAAGCTGATTGCTTCCAAGATATTCAAAAATTCATCCTCTTACAGCGTGCCTGGCTTTCTTTTGAAAGCTGGCGCTTATCTACTTGGCGATAGGCCTAATTAAGAAGCCTTTTATTTGATTAAGAGATGTTCTTATAGAAGTAAGAGCGTCTTTTTTGCGCAGGATTATTCTGTCGCCAGTTTTTTCTATGATTTTAACACTATAATTTTATGGAGAAAAGATGTTCAAACATAAACATCCTTTTGGGGGAGCGTTCCTTCCCGAAGAACTATTAGCCCCTATACAGAATCTAAAAGCGGAATGGGAGATTCTCAAAACTCAGCAAAGTTTTTTATCTGAACTAGATTGTATTTTGAAAAACTATGCGGGGAGACAAACTCCTCTGACTGAAGTTAAGAATTTTGCTCGAGCTATTGATGGCCCTAGAGTATTTCTTAAACGCGAAGATCTTTTGCATACAGGAGCACATAAACTGAATAATGCTCTTGGTCAGGNTTGCTTGCTAATATCTTGGGAANNACGTGTTGAGCTGAANNGGTNCGGNACANATGGNTAGCACNNCANAGCTGGNCTATCTAGGANNANNNGGGNNNNAACCTGGGNNNAAAANNGGNNNNANNAANCCAATGAANNAANNNNNTTNNNGGNCNNNNNNTTNNGAACAANGNNNNNGGNATCAAANGCGTNANCNNNTCCAAATGGGCNNNNNNNNTNNNNNNNNGGNNNNNNNNNNNNNTNCNTNCNNANNNNNTTNNCNNGNNNNNNNNNNNGNGNCCNNNNGGNAATCNNNNNGNNNTCCANNCNNNNNNNN

>T3

NNNNNNTNNNNNNGGGCAGCAGTGGTATCAACGCAGAGTACATGGGATTACAAAAAGGTGCGATATGAAAAATCAAGAGGAGTCTGGCTGGCAAGCTTTTCTGACATTATGCTCTAAAATGCAAAAAGAAAAGTTTTTACAAGACCTTTTTTCGCTGTTTTTGTCTTTTGGCGAACGTAAAGATGTCGCTTCTCGCTATCATATCATTCGAGCTCTTTTAGAAGGGGAGCTCACTCAAAGAGAGATAGCAGAGAAATACGGAGTCAGTATCGCACAAATTACCAGAGGATCTAATGCCCTTAAAGGATCAGATCCTCAATTTAAAGAGTTTTTACAAAAAGAGATCTGATCTTCTTTTGTAAAATACAAATAAGATTGAAAGTATTTGTATGCACGCGTTGTTAATGAACAAATATTCTGTTTTAGCAGTTTTGGTACGTAAGTATAGCTGCAGCATGCCATGCAAATCAGCTTTTCAAGCTGATTGCTTCCAAGATATTCAAAAATTCATCCTCTTACAGCGTGCCTGGCTTTCTTTTGAAAGCTGGCGCTTATCTACTTGGCGATAGGCCTAATTAAGAAGCCTTTTATTTGATTAAGAGATGTTCTTATAGAAGTAAGAGCGTCTTTTTTGCGCAGGATTATTCTGTCGCCAGTTTTTTCTATGATTTTAACACTATAATTTTATGGAGAAAAGATGTTCAAACATAAACATCCTTTTGGGGGAGCGTTCCTTCCCGAAGAACTATTAGCCCCTATACAGAATCTAAAAGCGGAATGGGAGATTCTCAAAACTCAGCAAAGTTTTTTATCTGAACTAGATTGTATTTTGAAAAACTATGCGGGGAGACAAACTCCTCTGACTGAAGTTAAGAATTTTGCTCGAGCTATTGATGGGCCTAGAGTATTTCTTAAACGCGAAGATCTTTTGCNTACAGGNNNACTNNNNCTNNNTAANGNNCTTGGANNGNNTTGNNTGNNNNNNCNNNGGNAANNNNNNNTTGAACTNANNNGGNNNNACNNNNNGNNNN

>T4

NNNGNNTNNNNNNGGGCAGCAGTGGTATCAACGCAGAGTACATGGGACAAAAAGGTGCGATATGAAAAATCAAGAGGAGTCTGGCTGGCAAGCTTTTCTGACATTATGCTCTAAAATGCAAAAAGAAAAGTTTTTACAAGACCTTTTTTCGCTGTTTTTGTCTTTTGGCGAACGTAAAGATGTCGCTTCTCGCTATCATATCATTCGAGCTCTTTTAGAAGGGGAGCTCACTCAAAGAGAGATAGCAGAGAAATACGGAGTCAGTATCGCACAAATTACCAGAGGATCTAATGCCCTTAAAGGATCAGATCCTCAATTTAAAGAGTTTTTACAAAAAGAGATCTGATCTTCTTTTGTAAAATACAAATAAGATTGAAAGTATTTGTATGCACGCGTTGTTAATGAACAAATATTCTGTTTTAGCAGTTTTGGTACGTAAGTATAGCTGCAGCATGCCATGCAAATCAGCTTTTCAAGCTGATTGCTTCCAAGATATTCAAAAATTCATCCTCTTACAGCGTGCCTGGCTTTCTTTTGAAAGCTGGCGCTTATCTACTTGGCGATAGGCCTAATTAAGAAGCCTTTTATTTGATTAAGAGATGTTCTTATAGAAGTAAGAGCGTCTTTTTTGCGCAGGATTATTCTGTCGCCAGTTTTTTCTATGATTTTAACACTATAATTTTATGGAGAAAAGATGTTCAAACATAAACATCCTTTTGGGGGAGCGTTCCTTCCCGAAGAACTATTAGCCCCTATACAGAATCTAAAAGCGGAATGGGAGATTCTCAAAACTCAGCAAAGTTTTTTATCTGAACTAGATTGTATTTTGAAAAACTATGCGGGGAGACAAACTCCTCTGACTGAAGTTAAGAATTTTACTCGAGCTATTGATGGCCCTAGAGTATTTCTTAAACGCGAAGATCTTTNGCATANNGNAGCACATAAACTGAATAATGCTCTTGGTCNNTGTTTGCTTGCTAAATATCTTGGNAAANNACGTGTTGTAGCTGAANCAGGTNNGGNACACATGGNGTAGCACAGCANNNCGNNNNTNANNAGGATNNNTTGNNNNNNCNTGGGAGCAAAANNNGGANNNNAAANCCANNNNNNNNNNNGNCTTTTNNGGNNNNNNCCNNNNNNNANNNNNNTTNNN

>T5

NNNNNNTNNNNNAGGGNAGCAGTGGTATCAACGCAGAGTACATGGGACAAAAAGGTGCGATATGAAAAATCAAGAGGAGTCTGGCTGGCAAGCTTTTCTGACATTATGCTCTAAAATGCAAAAAGAAAAGTTTTTACAAGACCTTTTTTCGCTGTTTTTGTCTTTTGGCGAACGTAAAGATGTCGCTTCTCGCTATCATATCATTCGAGCTCTTTTAGAAGGGGAGCTCACTCAAAGAGAGATAGCAGAGAAATACGGAGTCAGTATCGCACAAATTACCAGAGGATCTAATGCCCTTAAAGGATCAGATCCTCAATTTAAAGAGTTTTTACAAAAAGAGATCTGATCTTCTTTTGTAAAATACAAATAAGATTGAAAGTATTTGTATGCACGCGTTGTTAATGAACAAATATTCTGTTTTAGCAGTTTTGGTACGTAAGTATAGCTGCAGCATGCCATGCAAATCAGCTTTTCAAGCTGATTGCTTCCAAGATATTCAAAAATTCATCCTCTTACAGCGTGCCTGGCTTTCTTTTGAAAGCTGGCGCTTATCTACTTGGCGATAGGCCTAATTAAGAAGCCTTTTATTTGATTAAGAGATGTTCTTATAGAAGTAAGAGCGTCTTTTTTGCGCAGGATTATTCTGTCGCCAGTTTTTTCTATGATTTTAACACTATAATTTTATGGAGAAAAGATGTTCAAACATAAACATCCTTTTGGGGGAGCGTTCCTTCCCGAAGAACTATTAGCCCCTATACAGAATCTAAAAGCGGAATGGGAGATTCTCAAAACTCAGCAAAGTTTTTTATCTGAACTAGATTGTATTTTGAAAAACTATGCGGGGAGACAAACTCCTCTGACTGAAGTTAAGAATTTTGCTCGAGCTATTGATGGCCCTAGAGTATTNCTTAAACGCGAAGATCTTTTGCNTANNGNAGCACATAAACTGAATAATGCTCTTGGTNNNTGTTTGCTTGCAANTNNNNNGGNAANCCNGTGTTGAACNNAACAGNNNNGNACNCANGNNTNGCNNCGNACAACGNGNNNNNNNAGGATANNTTGGNNNNNNNNNGGNAGCAAANNNGNNANNNNANNNNCCNNNNNNNANNNGNNNNNNANNGGN

>T6

NNNGNNCTNCNNNNAGGGCAGCAGTGGTATCAACGCAGAGTACATGGGCTTACAAAAAGGTGCGATATGAAAAATCAAGAGGAGTCTGGCTGGCAAGCTTTTCTGACATTATGCTCTAAAATGCAAAAAGAAAAGTTTTTACAAGACCTTTTTTCGCTGTTTTTGTCTTTTGGCGAACGTAAAGATGTCGCTTCTCGCTATCATATCATTCGAGCTCTTTTAGAAGGGGAGCTCACTCAAAGAGAGATAGCAGAGAATACGGAGTCAGTATCGCACAAATTACCAGAGGATCTAATGCCCTTAAAGGATCAGATCCTCAATTTAAAGAGTTTTTACAAAAAGAGATCTGATCTTCTTTTGTAAAATACAAATAAGATTGAAAGTATTTGTATGCACGCGTTGTTAATGAACAAATATTCTGTTTTAGCAGTTTTGGTACGTAAGTATAGCTGCAGCATGCCATGCAAATCAGCTTTTCAAGCTGATTGCTTCCAAGATATTCAAAAATTCATCCTCTTACAGCGTGCCTGGCTTTCTTTTGAAAGCTGGCGCTTATCTACTTGGCGATAGGCCTAATTAAGAAGCCTTTTATTTGATTAAGAGATGTTCTTATAGAAGTAAGAGCGTCTTTTTTGCGCAGGATTATTCTGTCGCCAGTTTTTTCTATGATTTTAACACTATAATTTTATGGAGAAAAGATGTTCAAACATAAACATCCTTTTGGGGGAGCGTTCCTTCCCGAAGAACTATTAGCCCCTATACAGAATCTAAAAGCGGAATGGGAGATTCTCAAAACTCAGCAAAGTTTTTTATCTGAACTAGATTGTATTTTGAAAAACTATGCGGGGAGACAAACTCCTCTGACTGAAGTTAAGAATTTTGCTCGAGCTATTGATGGCCCTAGAGTATTTCTTAAACGCGAAGATCTTTTGCATACAGGAGCACATAAACTGAATAATGCTCTTGGTCAGTGTTTGCTTGCTAATATCTTGGGAAAACACGTGTTNNNGCTGAANNANGTNCGGGNNNNCATGGANANCANCNGCACNNNNGGGNNTATCTAGGATTAGANNGGNNGNNNNATGGGAGCAAAGATGGNACGNNGAANCCAATGGANNAANNNNNNTTTNNNGGNNNNNNNNNTTNNNNNNAANNGGNNNNNNGGNCNAANNNNNNTNNNNNNNTNCAANTGNNNNNNNCNNNTTTNNNNNNNGNTNNNNNNNNNNCTTNNNNNCNNNNNNNNNTTNNNNNNNNNNGNNNNNNNNNNNNNCNNNNNNNNNNNNNNNNNNGNNNNNNNNNNNCNNNTNNNNNNNNNNNGTTCNNNNNNNNNNNNNNNNN

>T7

NNNNNNTNNNNNNGGGNAGCAGTGGTATCAACGCAGAGCACATGGGTACAAAAAGGTGCGATATGAAAAATCAAGAGGAGTCTGGCTGGCAAGCTTTTCTGACATTATGCTCTAAAATGCAAAAAGAAAAGTTTTTACAAGACCTTTTTTCGCTGTTTTTGTCTTTTGGCGAACGTAAAGATGTCGCTTCTCGCTATCATATCATTCGAGCTCTTTTAGAAGGGGAGCTCACTCAAAGAGAGATAGCAGAGAAATACGGAGTCAGTATCGCACAAATTACCAGAGGATCTAATGCCCTTAAAGGATCAGATCCTCAATTTAAAGAGTTTTTACAAAAAGAGATCTGATCTTCTTTTGTAAAATACAAATAAGATTGAAAGTATTTGTATGCACGCGTTGTTAATGAACAAATATTCTGTTTTAGCAGTTTTGGTACGTAAGTATAGCTGCAGCATGCCATGCAAATCAGCTTTTCAAGCTGATTGCTTCCAAGATATTCAAAAATTCATCCTCTTACAGCGTGCCTGGCTTTCTTTTGAAAGCTGGCGCTTATCTACTTGGCGATAGGCCTAATTAAGAAGCCTTTTATTTGATTAAGAGATGTTCTTATAGAAGTAAGAGCGTCTTTTTTGCGCAGGATTATTCTGTCGCCAGTTTTTTCTATGATTTTAACACTATAATTTTATGGAGAAAAGATGTTCAAACATAAACATCCTTTTGGGGGGAGCGTTCCTTCCCGAAGAACTATTAGCCCCTATACAGAATCTAAAAGCGGAAATGGGAGATTCTCAAAACTCAGCAAAGTTTTTTATCTGAACTAGATTGTATTTTGAAAAACTATGCCGGGGAGACAAACTCCTCTGACTGAAGTTAAGAATTTTGCTCGAGCTATTGATGGCCCTAGAGTATTTCTTAAACGCGAAGATCTTTTGCATACAGGAGCACATAAACTGAATAATGCTCTTGGNCAGTGTTNGCTTGCTAAANATCTTGGGAAANCANGTGTTNNAGCTGAAACAGGTGCGGNNNNNCATGGNGTANNNNNNNNNNCAGCGTGTNNTNNTCTAGGANANNNTTGGNNNNNNNNATGGGAGCAANANNNGGNAACGACNAANCCAATNNAANNNNTNNNNTTNNNNNNNANNNNNTTNNNNNNAANGNNNNNGGNANNAANTGNNTTNNCAGCTTCNANNTGGNNNNNNNNNNTTNNCCNNNNNNNNNANCNCNNNNNNTNNNNNCCNANNNNNNNTTNNNNNNNANNNNNNNNGAANNNNNGNTGNNANNCNNNNNTNNNNNTNNGGNNNNNTTTNNNNNNTNNNNNN

>T8

NNNNNNNNNNNNNNGGNNNNCAGTGGTATCAACGCAGAGTACATGGGCTTACAAAAAGGTGCGATATGAAAAATCAAGAGGAGTCTGGCTGGCAAGCTTTTCTGACATTATGCTCTAAAATGCAAAAAGAAAAGTTTTTACAAGACCTTTTTTCGCTGTTTTTGTCTTTTGGCGAACGTAAAGATGTCGCTTCTCGCTATCATATCATTCGAGCTCTTTTAGAAGGGGAGCTCACTCAAAGAGAGATAGCAGAGAAATACGGAGTCAGTATCGCACAAATTACCAGAGGATCTAATGCCCTTAAAGGATCAGATCCTCAATTTAAAGAGTTTTTACAAAAAGAGATCTGATCTTCTTTTGTAAAATACAAATAAGATTGAAAGTATTTGTATGCACGCGTTGTTAATGAACAAATATTCTGTTTTAGCAGTTTTGGTACGTAAGTATAGCTGCAGCATGCCATGCAAATCAGCTTTTCAAGCTGATTGCTTCCAAGATATTCAAAAATTCATCCTCTTACAGCGTGCCTGGCTTTCTTTTGAAAGCTGGCGCTTATCTACTTGGCGATAGGCCTAATTAAGAAGCCTTTTATTTGATTAAGAGATGTTCTTATAGAAGTAAGAGCGTCTTTTTTGCGCAGGATTATTCTGTCGCCAGTTTTTTCTATGATTTTAACACTATAATTTTATGGAGAAAAGATGTTCAAACATAAACATCCTTTTGGGGGAGCGTTCCTTCCCGAAGAACTATTAGCCCCTATACAGAATCTAAAAGCGGAATGGGAGATTCTCAAAACTCAGCAAAGTTTTTTATCTGAACTAGATTGTATTTTGAAAAACTATGCGGGGAGACAAACTCCTCTGACTGAAGTTAAGAATTTTGCTCGAGCTATTGATGGCCCTAGAGTATTTCTTAAACGCGAAGATCTTTTGCATACAGGAGCACATAAACTGAATAATGCTCTTGGTCAGTGTTGCTTGCTAATATCTTGGNAANNACGTGTTGAGCTGAAANAGNNCGGGACANNTGGATNGCACAGCANAGCNGNNCTNTCTAGGATTAGATGGGNNNNAACATGGGAGCAAAGATGGNACGNNGAANCCAATNNNNNANTNNCCTTTNNGGNCNAATCTNNNNNAACAANGGNNNNGNNNNNAANNGCGTNNNNNNNNTCAANTGGGNAACCCNNNNTTNCCCNNTNNNNNANNCNNGNANNNNNNNNNTNNNNNNNNNTNNNNNGNTNAANNNNNNNNAANNNNNN

May 11 2017

>B1

NNNNNNNNNNNNNGGNNNNCAGTGGTATCAACGCAGAGTACATGGGTCTGTCGCCAGTTTTTTCTATGATTTTAACACTATAATTTTATGGAGAAAAGATGTTCAAACATAAACATCCTTTTGGGGGAGCGTTCCTTCCCGAAGAACTATTAGCCCCTATACAGAATCTAAAAGCGGAATGGGAGATTCTCAAAACTCAGCAAAGTTTTTTATCTGAACTAGATTGTATTTTGAAAAACTATGCGGGGAGACAAACTCCTCTGACTGAAGTTAAGAATTTTGCTCGAGCTATTGATGGCCCTAGAGTATTTCTTAAACGCGAAGATCTTTTGCATACAGGAGCACATAAACTGAATAATGCTCTTGGTCAGTGTTTGCTTGCTAAATATCTTGGGAAAACACGTGTTGTAGCTGAAACAGGTGCGGGACAACATGGAGTAGCAACAGCAACAGCGTGTGCTTATCTAGGATTAGATTGTGTAGTATACATGGGAGCAAAAGATGTGGAACGACAGAAACCAAATGTAGAGAAAATGCGCTTTTTAGGTGCTGAAGTCGTTTCTGTAACAAAAGGATCTTGTGGACTCAAAGATGCAGTTAATCAAGCTCTACAAGATTGGGCAACAACACACTCATTTACTCACTATTGCTTAGGATCGGCCTTAGGACCTTTACCTTATCCCGATATCGTTCGATTTTTTCAGTCTGTTATAAGCGCTGAAGTGAAAGAGCAAATCCATGCAGTTGCAGGAAGAGATCCTGATATTCTGATTGCATGTATCGGAGGTGGCTCCAACGCTATTGGATTTTTCCATCATTTTATCCCGAATCCAAAAGTCCAATTAATTGGAGTGGAAGGGGGAGGACTGGGCATTTCTTCAGGAAAACATGCAGCACGTTTTGCAACAGGGCGACCTGGAGTATTCCACGAAGCTTGGCGTAATCATGGTCATAGCTGTTTCCTGTGTGAAATTGTTATCCGCTCACAATTCCCNNANCATACGAGCCGGAAGCNNAANGTGTAAAGCCTGGGNTNCCTAATGAGTGANNNAACTNANNTTAATTGNNNNNNNNNNNNTGCCNNNTTTNCAGTCGGGAANCTNNCNGGCNNNNNNTNAATGNNCGGCCAANNNNNGGGNNNNNNGGTTNNNNTNGGNNNNNTCCGNTNNNNNNNNNNAACNNNNNNNNGGNNNNNNGNNGGNNNNNNNNNNNNNNNNNANNGGGGNNNNNNNNNNCCNNNNNNNGGGNNNNCNGAAANNNNNNNNNNNNNNCNNNNNGNNNNNNNNNNNNNNGTNNNNTNNGTNNNNNNNNNCCNNNNNNNNNNNNNANNNNNNNNNNNNNNNNNNN

>B2

NNNNNNNNNNNNNGGNNNNCAGTGGTATCAACGCAGAGTACATGGGTTAACACTATAATTTTATGGAGAAAAGATGTTCAAACATAAACATCCTTTTGGGGGAGCGTTCCTTCCCGAAGAACTATTAGCCCCTATACAGAATCTAAAAGCGGAATGGGAGATTCTCAAAACTCAGCAAAGTTTTTTATCTGAACTAGATTGTATTTTGAAAAACTATGCGGGGAGACAAACTCCTCTGACTGAAGTTAAGAATTTTGCTCGAGCTATTGATGGCCCTAGAGTATTTCTTAAACGCGAAGATCTTTTGCATACAGGAGCACATAAACTGAATAATGCTCTTGGTCAGTGTTTGCTTGCTAAATATCTTGGGAAAACACGTGTTGTAGCTGAAACAGGTGCGGGACAACATGGAGTAGCAACAGCAACAGCGTGTGCTTATCTAGGATTAGATTGTGTAGTATACATGGGAGCAAAAGATGTGGAACGACAGAAACCAAATGTAGAGAAAATGCGCTTTTTAGGTGCTGAAGTCGTTTCTGTAACAAAAGGATCTTGTGGACTCAAAGATGCAGTTAATCAAGCTCTACAAGATTGGGCAACAACACACTCATTTACTCACTATTGCTTAGGATCGGCCTTAGGACCTTTACCTTATCCCGATATCGTTCGATTTTTTCAGTCTGTTATAAGCGCTGAAGTGAAAGAGCAAATCCATGCAGTTGCAGGAAGAGATCCTGATATTCTGATTGCATGTATCGGAGGTGGCTCCAACGCTATTGGATTTTTCCATCATTTTATCCCGAATCCAAAAGTCCAATTAATTGGAGTGGAAGGGGGGAGGACTGGGCATTTCTTCAGGAAAACATGCAGCACGTTTTGCAACAGGGCGACCTGGAGTATTCCACGAAGCTTGGCGTAATCATGGTCATAGCTGTTTCCTGTGTGAAATTGTTATCCGCTCACAATTCCNNNANCATACGAGCCGGAAGCATAANGTGTAANGCCTGGGNNGCNTAATGAGTGNNNANCTCACNTTAATTGCGTTNNGCNNCTGCCNNNTTNNAGTCGGGAANCNNNNNGGCNNNTGCANAATGATCGNCNNNNNNNCCGGGNNNGGCGGTTNNNNTTGGNNNNTNNCNNTNCNNNNNCCTNNNNNNNNNNNNGGNTNNNGGTGGGGNNGGGNNNNNNNNNNNAAGGGGNNNNNNNTNNCCNANNNNNGGNNNNNNNNNNANNNTNNNNNNNNNCCNAANNNNNNNNNGNAANGGNNNNNNNGNTNNNNNNNCCNGCNNNNNNNANNNNTNNNNNNNNNNNNNNNCNNNNNNNNNNNNNNCNNNNNN

>B3

NNNNNNNNNNNNNNGGNNNNCAGTGGTATCAACGCAGAGTACATGGGCTATGATTTTAACACTATAATTTTATGGAGAAAAGATGTTCAAACATAAACATCCTTTTGGGGGAGCGTTCCTTCCCGAAGAACTATTAGCCCCTATACAGAATCTAAAAGCGGAATGGGAGATTCTCAAAACTCAGCAAAGTTTTTTATCTGAACTAGATTGTATTTTGAAAAACTATGCGGGGAGACAAACTCCTCTGACTGAAGTTAAGAATTTTGCTCGAGCTATTGATGGCCCTAGAGTATTTCTTAAACGCGAAGATCTTTTGCATACAGGAGCACATAAACTGAATAATGCTCTTGGTCAGTGTTTGCTTGCTAAATATCTTGGGAAAACACGTGTTGTAGCTGAAACAGGTGCGGGACAACATGGAGTAGCAACAGCAACAGCGTGTGCTTATCTAGGATTAGATTGTGTAGTATACATGGGAGCAAAAGATGTGGAACGACAGAAACCAAATGTAGAGAAAATGCGCTTTTTAGGTGCTGAAGTCGTTTCTGTAACAAAAGGATCTTGTGGACTCAAAGATGCAGTTAATCAAGCTCTACAAGATTGGGCAACAACACACTCATTTACTCACTATTGCTTAGGATCGGCCTTAGGACCTTTACCTTATCCCGATATCGTTCGATTTTTTCAGTCTGTTATAAGCGCTGAAGTGAAAGAGCAAATCCATGCAGTTGCAGGAAGAGATCCTGATATTCTGATTGCATGTATCGGAGGTGGCTCCAACGCTATTGGATTTTTCCATCATTTTATCCCGAATCCAAAAGTCCAATTAATTGGAGTGGANGGGGNNAGGACTGGGCATTTCTTCAGGAAAACATGCAGCACGTTTTGCAACAGGGCGACCTGGAGTATTCCACGAAGCTTGGCGTAATCATGGTCATAGCTGTTTCCTGTGTGAAATTGTTATCCGCTCACAATTCCACACAACATACGAGCCGGAAGCATAAAGTGTAANGCCTGGGNTNCNTAATGAGTGAGCTACTCACATTAATTGCGTTNNNNNNCTGCCCNTNNCCAGTCGGGAANNTGTCTGGCNNNTGCNNAATGATNNNCCNNNNCCNGGNNNNGNNNNGNTTNNNNNTTGGNNNNTNNCCNTTNNNNNNNNNGNNNNNNGGNNGGNNNNNNGNNNGGGGNAGGGNNNNNNNNNNNAAGGGGNNNNNNNNNNCNNNNNNNNNGGNANCNNNNNAANNTNNNNNANNNNCNNNNNNNNNNNNCNNNNANGNNNNNNNNNGNTTNNNNNNGNCNNNNNNNNNNNNCANCNNNNNNNNNNNNNGNNNNNNNNNNNNNNNNNNNNNNCCNNNNNNNNNN

>B4

NNNNNTNNNNNNGGNNNNCAGTGGTATCAACGCAGAGTACACGGGGAACATAAACATCCTTTTGGGGGAGCGTTCCTTCCCGAAGAACTATTAGCCCCTATACAGAATCTAAAAGCGGAATGGGAGATTCTCAAAACTCAGCAAAGTTTTTTATCTGAACTAGATTGTATTTTGAAAAACTATGCGGGGAGACAAACTCCTCTGACTGAAGTTAAGAATTTTGCTCGAGCTATTGATGGCCCTAGAGTATTTCTTAAACGCGAAGATCTTTTGCATACAGGAGCACATAAACTGAATAATGCTCTTGGTCAGTGTTTGCTTGCTAAATATCTTGGGAAAACACGTGTTGTAGCTGAAACAGGTGCGGGACAACATGGAGTAGCAACAGCAACAGCGTGTGCTTATCTAGGATTAGATTGTGTAGTATACATGGGAGCAAAAGATGTGGAACGACAGAAACCAAATGTAGAGAAAATGCGCTTTTTAGGTGCTGAAGTCGTTTCTGTAACAAAAGGATCTTGTGGACTCAAAGATGCAGTNAATCAAGCTCTACAAGATTGGTCAACGACNCACTCATTTACTCACTNTNGCTTACGATNNNNNNNANNNNNTNNACCNNNNCCNGATNNNNNNNNNNNTTNATCANNNN

>B5

NNNNNTNNNNNNGGNNNNCAGTGGTATCAACGCAGAGTACATGGGCAAACATAAACATCCTTTTGGGGGAGCGTTCCTTCCCGAAGAACTATTAGCCCCTATACAGAATCTAAAAGCGGAATGGGAGATTCTCAAAACTCAGCAAAGTTTTTTATCTGAACTAGATTGTATTTTGAAAAACTATGCGGGGAGACAAACTCCTCTGACTGAAGTTAAGAATTTTGCTCGAGCTATTGATGGCCCTAGAGTATTTCTTAAACGCGAAGATCTTTTGCATACAGGAGCACATAAACTGAATAATGCTCTTGGTCAGTGTTTGCTTGCTAAATATCTTGGGAAAACACGTGTTGTAGCTGAAACAGGTGCGGGACAACATGGAGTAGCAACAGCAACAGCGTGTGCTTATCTAGGATTAGATTGTGTAGTATACATGGGAGCAAAAGATGTGGAACGACAGAAACCAAATGTAGAGAAAATGCGCTTTTTAGGTGCTGAAGTCGTTTCTGTAACAAAAGGATCTTGTGGACTCGAAGATGCAGTTAATCAAGCTCTACAAGATTGGGCAACAACACACTCATTTACTCACTATTGCTTAGGATCGGCCTTAGGACCTTTACCTTATCCCGATATCGTTCGATTTTTTCAGTCTGTTATAAGCGCTGAAGTGAAAGAGCAAATCCATGCAGTTGCAGGAAGAGATCCTGATATTCTGATTGCATGTATCGGAGGTGGCTCCAACGCTATTGGATTTTTCCATCATTTTATCCCGAATCCAAAAGTCCAATTAATTGGAGTGGAAAGGGGGGAGGACTGGNGCATTTCTTCAGGAAAACATGCAGCACGTTTTGCAACAGGGCGACCTGGAGTATTCCACGAAGCTTGGCGTAATCATGGTCATAGCTGTTTCCTGTGTGAAATTGTTATCCGCTCACAATTCCACACAACATACGAGCCGGAAGCATAAAGTGTAANGCCTGGGNTGCCTAATGAGTGAGCTANNNNNCATTAATTGCGTTGCNNNNNTGCCNGCTTTCNNTCGGNAACCTGTCTGGCNNNNNNANANTGATNNNCCNNNNNNGGGNAANNNGGTTNGNNNTTGGGNCCNNTCCNNTNNNNNNNNNGNACCCNTNNNNNGNNNTNNGNNGGNNNNGNNNNNNNNNNNNNNANGGGGNANNNGGTTNCCNANANNNGGGNANCCNNGNAANNNNNNNNNNANNNCCNNAANNNANNNNTNANNNNNGNNNGGGNNTNNNNANNNCCNNNNNNNNNNNANNNNNNNNNNNNNNGNNNNCNNNNNNNAANNNNNNNCNNNNNNNNNNNNNNANNNNNNNNNNNGNNNNNNNNNNNNNNNNNNNNNNNNNNNNNNNAGNN

>B6

NNNNNNNNNNNNNGGNNNNCAGTGGTATCAACGCAGAATGCATGGGTTTAACACTATAATTTTATGGAGAAAAGNNGTTCAAACATAAACATCCTTTTGGGGGAGCGTTCCTTCCCGAAGAACTATTAGCCCCTATACAGAATCTAAAAGCGGAATGGGAGATTCTCAAAACTCAGCAAAGTTTTTTATCTGAACTAGATTGTATTTTGAAAAACTATGCGGGGAGACAAACTCCTCTGACTGAAGTTAAGAATTTTGCTCGAGCTATTGATGGCCCTAGAGTATTTCTTAAACGCGAAGATCTTTTGCATACAGGAGCACATAAACTGAATAATGCTCTTGGTCAGTGTTTGCTTGCTAAATATCTTGGGAAAACACGTGTTGTAGCTGAAACAGGTGCGGGACAACATGGAGTAGCAACAGCAACAGCGTGTGCTTATCTAGGATTAGATTGTGTAGTATACATGGGAGCAAAAGATGTGGAACGACAGAAACCAAATGTAGAGAAAATGCGCTTTTTAAGTGCTGAAGTCGTTTCTGTAACAAAAGGATCTTGTGGACTCAAAGATGCAGTTAATCAAGCTCTACAAGATTGGGCAACAACACACTCATTTACTCACTATTGCNNANNATCGGCCTTATGGACCTTTACCTTATCCCGATATCGNNCNNNNNGNNCNNNNCTNNNATAAGCGNNGAAGCNNNNNNNNNGTTCNNNTNNNGNNNNCNNCNNN

>B7

NNNNNNNNNNNNNNNGGNNNNNAGTGGTATCAACGCAGAGTACATGGGTTAACACTATAATTTTATGGAGAAAAGNTCGTTNAANCATAAACATCCTTTTGGGGGAGCGTTCCTTCCCGAAGAACTATTAGCCCCTATACAGAATCTAAAAGCGGAATGGGAGATTCTCAAAACTCAGCAAAGTTTTTTATCTGAACTAGATTGTATTTTGAAAAACTATGCGGGGAGACAAACTCCTCTGACTGAAGTTAAGAATTTTGCTCGAGCTATTGATGGCCCTAGAGTATTTCTTAAACGCGAAGATCTTTTGCATACAGGAGCACATAAACTGAATAATGCTCTTGGTCAGTGTTTGCTTGCTAAATATCTTGGGAAAACACGTGTTGTAGCTGAAACAGGTGCGGGACAACATGGAGTAGCAACAGCAACAGCGTGTGCTTATCTAGGATTAGATTGTGTAGTATACATGGGAGCAAAAGATGTGGAACGACAGAAACCAAATGTAGAGAAAATGCGCTTTTTAGGTGCTGAAGTCGTTTCTGTAACAAAAGGATCTTGTGGACTCAAAGATGCAGTTAATCAAGCTCTACAAGATTGGGCAACAACACACTCATTTACTCACTATTGCTTAGGATCGGCCTTAGGACCTTTACCTTATCCCGATATCGTTCGATTTTTTCAGTCTGTTATAAGCGCTGAAGTGAAAGAGCAAATCCATGCAGTTGCAGGAAGAGATCCTGATATTCTGATTGCATGTATCGGAGGTGGCTCCAACGCTATTGGATTTTTCCATCATTTTATCCCGAATCCAAAAGTCCAATTAATTGGAGTGGAAGGGGGGAGGACTGGGCATTTCTTCAGGAAAACATGCAGCACGTTTTGCAACAGGGCGACCTGGAGTATTCCACGAAGCTTGGCGTAATCATGGTCATAGCTGTTTCCTGGNTGAANTTGTTATCCGCTCACAATTCCACACAACATACGAGCCGGAAGCATAAAGTGTAANGCCTGGGNNGGCTAATGAGTGAGCTAANTCNNNTTAATTGCNTTNNNNNACTGCCNNNTTNCCAGTCGGGAANCCNNNNTNNNNNNNNNNTNAATGATCCNNCCNNNNNCCGGGGNAAGGCGGTTNNNNNTTGGGNNNNNTNCCNTTNTNNNNNNNNNANNNNNNGNNNNNNNNNCTTNGNTGGGNNNNGGNNNANNNNNNNNNANGGNNNNANNNNNNNNCCNNNNNNNNGGNNNNNNNNNGNANNATNNNNNNCANNNCNNNANNGNNANNNCNNNAANGGNNNNNNNGGNTTNNNNANNNCCCCNNNNNNNNNNNNNNNNNCNNNNNANNNGNNNNNNNNNNNNNNNNNNNNNNNNNNNNNNNNNNTTNATTNNC

>B8

NNNNNNNNNCNNNNNGGNNNNCAGTGGTATCAACGCAGAGTACATGGGCTATGATTTTAACACTATAATTTTATGGACAAANGATGTTCAAACATAAACATCCTTTTGGGGGAGCGTTCCTTCCCGAAGAACTATTAGCCCCTATACAGAATCTAAAAGCGGAATGGGAGATTCTCAAAACTCAGCAAAGTTTTTTATCTGAACTAGATTGTATTTTGAAAAACTATGCGGGGAGACAAACTCCTCTGACTGAAGTTAAGAATTTTGCTCGAGCTATTGATGGCCCTAGAGTATTTCTTAAACGCGAAGATCTTTTGCATACAGGAGCACATAAACTGAATAATGCTCTTGGTCAGTGTTTGCTTGCTAAATATCTTGGGAAAACACGTGTTGTAGCTGAAACAGGTGCGGGACAACATGGAGTAGCAACAGCAACAGCGTGTGCTTATCTAGGATTAGATTGTGTAGTATACATGGGAGCAAAAGATGTGGAACGACAGAAACCAAATGTAGAGAAAATGCGCTTTTTAGGTGCTGAAGTCGTTTCTGTAACAAAAGGATCTTGTGGACTCAAAGATGCAGTTAATCAAGCTCTACAAGATTGGGCAACAACACACTCATTTACTCACTATTGCTTAGGATCGGCCTTAGGACCTTTACCTTATCCCGATATCGTTCGATTTTTTCAGTCTGTTATAAGCGCTGAAGTGAAAGAGCAAATCCATGCAGTTGCAGGAAGAGATCCTGATATTCTGATTGCATGTATCGGAGGTGGCTCCAACGCTATTGGATTTTTCCATCATTTTATCCCGAATCCAAAAGTCCAATTAATTGGAGTGGAAGGGGGGAGGACTGGGCATTTCTTCAGGAAAACATGCAGCACGTTTTGCAACAGGGCGACCTGGAGTATTCCACGAAGCTTGGCGTAATCATGGTCATAGCTGTTTCCTGTGTGAAATTGTTATCCGCTCACAATTCCNNACAACATACGAGCCGGAAGCATAAAGTGTAAAGCCTGGGGNGCCTAATGAGTGAGCTAACTCNNATTAATTGCGTTNNNNNNCTGCCNNNTNNCAGTCGGGAANNTGTCGNNCNNNTGNNNAATGNNNGGCCANCNNNNGGGNAGAGNNGGTNNNNNTNGGGNNNNNNCCNTTNNNNNNCCTGANCNNNGGNNNNNNNNTNNGNNGGGGGNAGGNNNNNNNNNNTCAAGGGNNANNCGNTTNCNNNNNTNNNGGNNANNNNGNNANNNNNNNNNANNNNCNNNNNNNNNNNNNAAAANNNNNNNNNGNNTNNNNNNNGN

>M1

NNNNNNNNNNNNNAGGGNNNNNAGTGGTATCAACGCAGAGTACATGGGCTACTTGGCGATAGGCCTAATTAAGAAGCCTTTTATTTGATTAAGAGATGTTCTTATAGAAGTAAGAGCGTCTTTTTTGCGCAGGATTATTCTGTCGCCAGTTTTTTCTATGATTTTAACACTATAATTTTATGGAGAAAAGATGTTCAAACATAAACATCCTTTTGGGGGAGCGTTCCTTCCCGAAGAACTATTAGCCCCTATACAGAATCTAAAAGCGGAATGGGAGATTCTCAAAACTCAGCAAAGTTTTTTATCTGAACTAGATTGTATTTTGAAAAACTATGCGGGGAGACAAACTCCTCTGACTGAAGTTAAGAATTTTGCTCGAGCTATTGATGGCCCTAGAGTATTTCTTAAACGCGAAGATCTTTTGCATACAGGAGCACATAAACTGAATAATGCTCTTGGTCAGTGTTTGCTTGCTAAATATCTTGGGAAAACACGTGTTGTAGCTGAAACAGGTGCGGGACAACATGGAGTAGCAACAGCAACAGCGTGTGCTTATCTAGGATTAGATTGTGTAGTATACATGGGAGCAAAAGATGTGGAACGACAGAAACCAAATGTAGAGAAAATGCGCTTTTTAGGTGCTGAAGTCGTTTCTGTAACAAAAGGATCTTGTGGACTCAAAGATGCAGTTAATCAAGCTCTACAAGATTGGGCAACAACACACTCATTTACTCACTATTGCTTAGGATCGGCCTTAGGACCTTTACCTTATCCCGATATCGTTCGATTTTTTCAGTCTGTTATAAGCGCTGAAGTGAAAGAGCAAATCCATGCAGTTGCAGGAAGAGATCCTGATATTCTGATTGCATGTATCGGAGGTGGCTCCAACGCTATTGGATTTTTCCATCATTTTATCCCGAATCCAAANGTCCAATTAATTGGATGGNAANNGGGGAAGGACTGGGCATTTCTTCAGGAAACATGCAGCACGTTTTGCACAGGGCGACCTGGAGTATNNANGAAGCTTGGCGTATCATGGTNNNAGCTGTTTCCTGGGGNAATTGTATCCNCTCNAATTCCNNNANNNACGAGCCGGAAGCTAANNNNAANGCCNGGGNTGCCAATNNNNNNNNANNNNNNNNTNNNNNNCNNNNANNCCNNTTNNNNNNGNNAACNGNNNNNNNNNNTNNNNNNNNNNNNNCCCCNGGNNNNNNNNTTNNNNNNNGGNNNNNNTNNNNNNNNNNNNNNNNNNGNNNNNNNNNCNCGNNNNNNNNNNNNGGNNNNNNTTCN

>M2

NNNNNNTNNNNNNNGGNNNNNAGTGGTATCAACGCAGAGTACATGGGTCATCCTCTTACAGCGTGCCTGGCTTTCTTTTGAAAGCTGGCGCTTATCTACTTGGCGATAGGCCTAATTAAGAAGCCTTTTATTTGATTAAGAGATGTTCTTATAGAAGTAAGAGCGTCTTTTTTGCGCAGGATTATTCTGTCGCCAGTTTTTTCTATGATTTTAACACTATAATTTTATGGAGAAAAGATGTTCAAACATAAACATCCTTTTGGGGGAGCGTTCCTTCCCGAAGAACTATTAGCCCCTATACAGAATCTAAAAGCGGAATGGGAGATTCTCAAAACTCAGCAAAGTTTTTTATCTGAACTAGATTGTATTTTGAAAAACTATGCGGGGAGACAAACTCCTCTGACTGAAGTTAAGAATTTTGCTCGAGCTATTGATGGCCCTAGAGTATTTCTTAAACGCGAAGATCTTTTGCATACAGGAGCACATAAACTGAATAATGCTCTTGGTCAGTGTTTGCTTGCTAAATATCTTGGGAAAACACGTGTTGTAGCTGAAACAGGTGCGGGACAACATGGAGTAGCAACAGCAACAGCGTGTGCTTATCTAGGATTAGATTGTGTAGTATACATGGGAGCAAAAGATGTGGAACGACAGAAACCAAATGTAGAGAAAATGCGCTTTTTAGGTGCTGAAGTCGTTTCTGTAACAAAAGGATCTTGTGGACTCAAAGATGCAGTTAATCAAGCTCTACAAGATTGGGCAACAACACACTCATTTACTCACTATTGCTTAGGATCGGCCTTAGGACCTTTACCTTATCCCGATCGTTCGATTTTTTCAGTCTGTTATAAGCGCTGAAGTGAAAGAGCAAATCCATGCAGTTGCAGGAAGAGATCCTGATATTCTGATTGCATGTATCGGAGGTGGCTCCACGCTATTGGATTTNCCATCATTTATCCNGNTCCAAAGTCAATTATTGANNGNAAANGGGGNANGGACTGGNATTTCTTCAGGAANCATGCANNNNGTTTGCACAGGNCGANNGNAGNNTCNNNAANTTGGCGANNNNTGGTCTAGCTGGTNCCNNTNNAATTGTNNCNNNCNNNTTCCNNNNNNNNGNNNNNNNNNNAAGGTGAAGNNGGGNNNNNAANAGGAGCAACCNNNAANGGGTNNNNANNNCCNTTNNNNNGNNNNNNNNNGGNNNNTNAANGANNNNANCNNNNNNNNNNNTNNNNNGNNNNNNNNNCNNNNNNNNTNNNCNNNNNNNN

>M3

NNNNNNTNCCNNNNGGNNAGCAGTGGTATCAACGCAGAGTACATGGGCTACTTGGCGATAGGCCTAATTAAGAAGCCTTTTATTTGATTAAGAGATGTTCTTATAGAAGTAAGAGCGTCTTTTTTGCGCAGGATTATTCTGTCGCCAGTTTTTTCTATGATTTTAACACTATAATTTTATGGAGAAAAGATGTTCAAACATAAACATCCTTTTGGGGGAGCGTTCCTTCCCGAAGAACTATTAGCCCCTATACAGAATCTAAAAGCGGAATGGGAGATTCTCAAAACTCAGCAAAGTTTTTTATCTGAACTAGATTGTATTTTGAAAAACTATGCGGGGAGACAAACTCCTCTGACTGAAGTTAAGAATTTTGCTCGAGCTATTGATGGCCCTAGAGTATTTCTTAAACGCGAAGATCTTTTGCATACAGGAGCACATAAACTGAATAATGCTCTTGGTCAGTGTTTGCTTGCTAAATATCTTGGGAAAACACGTGTTGTAGCTGAAACAGGTGCGGGACAACATGGAGTAGCAACAGCAACAGCGTGTGCTTATCTAGGATTAGATTGTGTAGTATACATGGGAGCAAAAGATGTGGAACGACAGAAACCAAATGTAGAGAAAATGCGCTTTTTAGGTGCTGAAGTCGTTTCTGTAACAAAAGGATCTTGTGGACTCAAAGATGCAGTTAATCAAGCTCTACAAGATTGGGCAACAACACACTCATTTACTCACTATTGCTTAGGATCGGCCTTAGGACCTTTACCTTATCCCGATATCGTTCGATTTTTTCAGTCTGTTATAAGCGCTGAAGTGAAAGAGCAAATCCATGCAGTTGCAGGAAGAGATCCTGATATTCTGATTGCATGTATCGGAGGTGGCTCCAACGCTATTGGATTTTCCATCATTTTATCCCGAATCCAAAGTCCATNANNNGATGGNAANGGGGNNAGGACTGGGCATTTCTTCAGGAAACATGCAGCACGTTTTGCACAGGNCGACCTGGAGTATTCNNGANGCTTGGCNNATCATGGTCTAGCTGTTTCCTNNNTGAATTGTATCCNNCACNTTCNANNANCTACGAGCCGGAAGCNAANGTNAANGCNGGGGNTNNNNNNNNNNAGCTAANNNNTNNNTNNNNNNNNNANNNCCNNTTCCNNNGGNNNCNNNNNNNNNNGCTTAANNNCCGCNNCNGNGGNNANGGGNTTNNNNNGGNNNNNNNNNNTNNNNNNNNNNNNNNNNNTTNNNGNCNNNNNNANNNNNANNGGGNNNNNNNCNANNCNNNNNNNNNNNNNNNNNNGNC

>M4

NNNNNTNCNNNNGGNNANCAGTGGTATCAACGCAGAGTACATGGGACAGCGTGCCTGGCTTTCTTTTGAAAGCTGGCGCTTATCTACTTGGCGATAGGCCTAATTAAGAAGCCTTTTATTTGATTAAGAGATGTTCTTATAGAAGTAAGAGCGTCTTTTTTGCGCAGGATTATTCTGTCGCCAGTTTTTTCTATGATTTTAACACTATAATTTTATGGAGAAAAGATGTTCAAACATAAACATCCTTTTGGGGGAGCGTTCCTTCCCGAAGAACTATTAGCCCCTATACAGAATCTAAAAGCGGAATGGGAGATTCTCAAAACTCAGCAAAGTTTTTTATCTGAACTAGATTGTATTTTGAAAAACTATGCGGGGAGACAAACTCCTCTGACTGAAGTTAAGAATTTTGCTCGAGCTATTGATGGCCCTAGAGTATTTCTTAAACGCGAAGATCTTTTGCATACAGGAGCACATAAACTGAATAATGCTCTTGGTCAGTGTTTGCTTGCTAAATATCTTGGGAAAACACGTGTTGTAGCTGAAACAGGTGCGGGACAACATGGAGTAGCAACAGCAACAGCGTGTGCTTATCTAGGATTAGATTGTGTAGTATACATGGGAGCAAAAGATGTGGAACGACAGAAACCAAATGTAGAGAAAATGCGCTTTTTAGGTGCTGAAGTCGTTTCTGTAACAAAAGGATCTTGTGGACTCAAAGATGCAGTTAATCAAGCTCTACAAGATTGGGCAACAACACACTCATTTACTCACTATTGCTTAGGATCGGCCTTAGGACCTTTACCTTATCCCGATATCGTTCGATTTTTTCAGTCTGTTATAAGCGCTGAAGTGAAAGAGCAAATCCATGCAGTTGCAGGAAGAGATCCTGATATTCTGATTGCATGTATCGGAGGTGGCTCCAACGCTATTGGATTTNCCATCATTTATCCCNATCCAAAGTCAATTANTTGGGGGGNAAANGGGGNAAGACNGNNNNTTTTNNNGGAANCATGCAGCACGTTTTNNNNNGGNNNANNNNGA

>M5

NNNNNNNTNNNNNNGGNNNNCAGTGGTATCAACGCAGAGTGCATGGGTTACAGCGTGCCTGGCTTTCTTTTGAAAGCTGGCGCTTATCTACTTGGCGATAGGCCTAATTAAGAAGCCTTTTATTTGATTAAGAGATGTTCTTATAGAAGTAAGAGCGTCTTTTTTGCGCAGGATTATTCTGTCGCCAGTTTTTTCTATGATTTTAACACTATAATTTTATGGAGAAAAGATGTTCAAACATAAACATCCTTTTGGGGGAGCGTTCCTTCCCGAAGAACTATTAGCCCCTATACAGAATCTAAAAGCGGAATGGGAGATTCTCAAAACTCAGCAAAGTTTTTTATCTGAACTAGATTGTATTTTGAAAAACTATGCGGGGAGACAAACTCCTCTGACTGAAGTTAAGAATTTTGCTCGAGCTATTGATGGCCCTAGAGTATTTCTTAAACGCGAAGATCTTTTGCATACAGGAGCACATAAACTGAATAATGCTCTTGGTCAGTGTTTGCTTGCTAAATATCTTGGGAAAACACGTGTTGTAGCTGAAACAGGTGCGGGACAACATGGAGTAGCAACAGCAACAGCGTGTGCTTATCTAGGATTAGATTGTGTAGTATACATGGGAGCAAAAGATGTGGAACGACAGAAACCAAATGTAGAGAAAATGCGCTTTTTAGGTGCTGAAGTCGTTTCTGTAACAAAAGGATCTTGTGGACTCAAAGATGCAGTTAATCAAGCTCTACAAGATTGGGCAACAACACACTCATTTACTCACTATTGCTTAGGATCGGCCTTAGGACCTTTACCTTATCCCGATATCGTTCGATTTTTTCAGTCTGTTATAAGCGCTGAAGTGAAAGAGCAAATCCATGCAGTTGCAGGAAGAGATCCTGATATTCTGATTGCATGTATCGGAGGTGGCTCCAACGCTATTGGATTTNCCATCATTTATCCCNNTCCAAAGTCAATTATTGNNTNNNAAANGGGGNAAGNNNGGGNCATTTCTTCAGGAAACATGCAGCACGTTTTGCANAGGNCNNCCTGGAGTNTCCNNNAGCTTGGCGNNNNNTGGGCANNNNGGTTCCNNNGNNAATTGNNTCNNNNNNNTNCNNNNCNNNNANCCNGAANNNAANGTNANNCNNGGGNTGCCAANNNGGNNAACCANNNANNNCGTGCNNNNNNCCGNTNNAACGGNAANNNNNNNNNNNNNANGNANNNNNNNNNNGNNAAGGNTNNNNNNNGGCNNCNNNNNNNNNNNNNNNNNNNNTNNNNNNNNNNNAGANNCANNNNNGNNNNNNCANNNNNNNNNNNNNNNNCNNNNNNNNNNNNNTNNNNNNN

>M6

NNNNNNNTNNNNNNNGGNNNNCAGTGGTATCAACGCAGAGTACATGGGTTCAAAAATTCATCCTCTTACAGCGTGCCTGGCTTTCTTTTGAAAGCTGGCGCTTATCTACTTGGCGATAGGCCTAATTAAGAAGCCTTTTATTTGATTAAGAGATGTTCTTATAGAAGTAAGAGCGTCTTTTTTGCGCAGGATTATTCTGTCGCCAGTTTTTTCTATGATTTTAACACTATAATTTTATGGAGAAAAGATGTTCAAACATAAACATCCTTTTGGGGGAGCGTTCCTTCCCGAAGAACTATTAGCCCCTATACAGAATCTAAAAGCGGAATGGGAGATTCTCAAAACTCAGCAAAGTTTTTTATCTGAACTAGATTGTATTTTGAAAAACTATGCGGGGAGACAAACTCCTCTGACTGAAGTTAAGAATTTTGCTCGAGCTATTGATGGCCCTAGAGTATTTCTTAAACGCGAAGATCTTTTGCATACAGGAGCACATAAACTGAATAATGCTCTTGGTCAGTGTTTGCTTGCTAAATATCTTGGGAAAACACGTGTTGTAGCTGAAACAGGTGCGGGACAACATGGAGTAGCAACAGCAACAGCGTGTGCTTATCTAGGATTAGATTGTGTAGTATACATGGGAGCAAAAGATGTGGAACGACAGAAACCAAATGTAGAGAAAATGCGCTTTTTAGGTGCTGAAGTCGTTTCTGTAACAAAAGGATCTTGTGGACTCAAAGATGCAGTTAATCAAGCTCTACAAGATTGGGCAACAACACACTCATTTACTCACTATTGCTTAGGATCGGCCTTAGGACCTTTACCTTATCCCGATATCGTTCGATTTTTTCAGTCTGTTATAAGCGCTGAAGTGAAAGAGCAAATCCATGCAGTTGCAGGAAGAGATCCTGATATTCTGATTGCATGTATCGGAGGTGGCTCCAACGCTATTGGATTTTCCATCATTNATCCCGAATCCAAAGTCCATTAATTGGANNGNNAAGGGGNAGGACTGGGNNTTCTTNNGGAANCATGCANNNNNTTTGCACAGGNCGACTGGANTNNCCNNAANNTGGNGAATNNNGGCNNNNTNTNNCNNNTGGAATTGTATCNNNNANTTCCANANNNNNNNNNNNANNNANNTGNAANCNNGGNTGCCAAGGNGNNNNNNNNNNAATGCNNNNNNNNNNCNGNNNNNNNNNNAANNNNNNNCNNNNNNNNNNNNNNNNNNNNNNNNGNNNNNNNNNNNNNGNNNNNCNNCNNNNCGAANCNNNNNNNNNNNNNNNNGGNNNNNNNTNNNNNNNNNNNNNTN

>M7

NNNNNNTNNCNNNNGGGCAGCAGTGGTATCAACGCAGAGTACATGGGACAGCGTGCCTGGCTTTCTTTTGAAAGCTGGCGCTTATCTACTTGGCGATAGGCCTAATTAAGAAGCCTTTTATTTGATTAAGAGATGTTCTTATAGAAGTAAGAGCGTCTTTTTTGCGCAGGATTATTCTGTCGCCAGTTTTTTCTATGATTTTAACACTATAATTTTATGGAGAAAAGATGTTCAAACATAAACATCCTTTTGGGGGAGCGTTCCTTCCCGAAGAACTATTAGCCCCTATACAGAATCTAAAAGCGGAATGGGAGATTCTCAAAACTCAGCAAAGTTTTTTATCTGAACTAGATTGTATTTTGAAAAACTATGCGGGGAGACAAACTCCTCTGACTGAAGTTAAGAATTTTGCTCGAGCTATTGATGGCCCTAGAGTATTTCTTAAACGCGAAGATCTTTTGCATACAGGAGCACATAAACTGAATAATGCTCTTGGTCAGTGTTTGCTTGCTAAATATCTTGGGAAAACACGTGTTGTAGCTGAAACAGGTGCGGGACAACATGGAGTAGCAACAGCAACAGCGTGTGCTTATCTAGGATTAGATTGTGTAGTATACATGGGAGCAAAAGATGTGGAACGACAGAAACCAAATGTAGAGAAAATGCGCTTTTTAGGTGCTGAAGTCGTTTCTGTAACAAAAGGATCTTGTGGACTCAAAGATGCAGTTAATCAAGCTCTACAAGATTGGGCAACAACACACTCATTTACTCACTATTGCTTAGGATCGGCCTTAGGACCTTTACCTTATCCCGATATCGTTCGATTTTTTCAGTCTGTTATAAGCGCTGAAGTGAAAGAGCAAATCCATGCAGTTGCAGGAAGAGATCCTGATATTCTGATTGCATGTATCGGAGGTGGCTCCAACGCTATTGGATTTTTCCATCATTTTATCCCGAATCCAANAGTCNATTAATTGGNNNNNNAANGGGGNAGGACTGGGNATTTCTTCAGGAANCATGCN

>M8

NNNNNNNTNCNNNNNGGGNAGCAGTGGTATCAACGCAGAGTGCATGGGTTACAGCGTGCCTGGCTTTCTTTTGAAAGCTGGCGCTTATCTACTTGGCGATAGGCCTAATTAAGAAGCCTTTTATTTGATTAAGAGATGTTCTTATAGAAGTAAGAGCGTCTTTTTTGCGCAGGATTATTCTGTCGCCAGTTTTTTCTATGATTTTAACACTATAATTTTATGGAGAAAAGATGTTCAAACATAAACATCCTTTTGGGGGAGCGTTCCTTCCCGAAGAACTATTAGCCCCTATACAGAATCTAAAAGCGGAATGGGAGATTCTCAAAACTCAGCAAAGTTTTTTATCTGAACTAGATTGTATTTTGAAAAACTATGCGGGGAGACAAACTCCTCTGACTGAAGTTAAGAATTTTGCTCGAGCTATTGATGGCCCTAGAGTATTTCTTAAACGCGAAGATCTTTTGCATACAGGAGCACATAAACTGAATAATGCTCTTGGTCAGTGTTTGCTTGCTAAATATCTTGGGAAAACACGTGTTGTAGCTGAAACAGGTGCGGGACAACATGGAGTAGCAACAGCAACAGCGTGTGCTTATCTAGGATTAGATTGTGTAGTATACATGGGAGCAAAAGATGTGGAACGACAGAAACCAAATGTAGAGAAAATGCGCTTTTTAGGTGCTGAAGTCGTTTCTGTAACAAAAGGATCTTGTGGACTCAAAGATGCAGTTAATCAAGCTCTACAAGATTGGGCAACAACACACTCATTTACTCACTATTGCTTAGGATCGGCCTTAGGACCTTTACCTTATCCCGATATCGTTCGATTTTTTCAGTCTGTTATAAGCGCTGAAGTGAAAGAGCAAATCCATGCAGTTGCAGGAAGAGATCCTGATATTCTGATTGCATGTATCGGAGGTGGCTCCAACGCTATTGGATTTTTCCATCATTTTATCCCGAATCCAAAGTCCAATTAATTGGAGTGGAAGGGGNAGGACTGGGCATTTCTTNAGNAAACATGCAGCACGTTTNNNANNNGGNNNNCNTGGAGNNNNCCNNNNNNNTTGNCNNN

>T1

NNNNNNNNNNNNNNNNNGGNNNNGCAGTGGTATCAACGCAGAGTACATGGGACAAAAAGGTGCGATATGAAAAATCAAGAGGNGTCTGGCTGGCAAGCTTTTCTGACATTATGCTCTAAAATGCAAAAAGAAAAGTTTTTACAAGACCTTTTTTCGCTGTTTTTGTCTTTTGGCGAACGTAAAGATGTCGCTTCTCGCTATCATATCATTCGAGCTCTTTTAGAAGGGGAGCTCACTCAAAGAGAGATAGCAGAGAAATACGGAGTCAGTATCGCACAAATTACCAGAGGATCTAATGCCCTTAAAGGATCAGATCCTCAATTTAAAGAGTTTTTACAAAAAGAGATCTGATCTTCTTTTGTAAAATACAAATAAGATTGAAAGTATTTGTATGCACGCGTTGTTAATGAACAAATATTCTGTTTTAGCAGTTTTGGTACGTAAGTATAGCTGCAGCATGCCATGCAAATCAGCTTTTCAAGCTGATTGCTTCCAAGATATTCAAAAATTCATCCTCTTACAGCGTGCCTGGCTTTCTTTTGAAAGCTGGCGCTTATCTACTTGGCGATAGGCCTAATTAAGAAGCCTTTTATTTGATTAAGAGATGTTCTTATAGAAGTAAGAGCGTCTTTTTTGCGCAGGATTATTCTGTCGCCAGTTTTTTCTATGATTTTAACACTATAATTTTATGGAGAAAAGATGTTCAAACATAAACATCCTTTTGGGGGAGCGTTCCTTCCCGAAGAACTATTAGCCCCTATACAGAATCTAAAAGCGGAATGGGAGATTCTCAAAACTCAGCAAAGTTTTTTATCTGAACTAGATTGTATTTTGAAAAACTATGCGGGGAGACAAACTCCTCTGACTGAAGTTAAGAATTTTGCTCGAGCTATTGATGGCCCTAGAGTATTTCTTAAACGCGAAGATCTTTTGCATACAGGAGCACATAAACTGAATAATGCTCTTGGTCAGTGTTNGCTTGCTAAATATCTTGGGAAAACACGTGTTGTAGCTGAAACAGGTGCGGNANANCATGGAGTAGCAACAGCAACAGNNNNNNCTNNTCTAGGATTAGATTGGNNNGTAACATGGNNNCNAANNNTGGNNANNNNNNAANCCAANNNNANNAANNTNNNNTTTTNAGNNNNNANNNNNNNCTTGNNNNNNNNNNNNNGGNNNNNNNNNNNNNCNNNNNNNNNNNNNNNNNNGGNCNNNNNN

>T2

NNNNNNTNNNNNNGGGNNAGCAGTGGTATCAACGCAGAGTACATGGGGATTACAAAAAGGTGCGATATGAAAAATCAAGAGGAGTCTGGCTGGCAAGCTTTTCTGACATTATGCTCTAAAATGCAAAAAGAAAAGTTTTTACAAGACCTTTTTTCGCTGTTTTTGTCTTTTGGCGAACGTAAAGATGTCGCTTCTCGCTATCATATCATTCGAGCTCTTTTAGAAGGGGAGCTCACTCAAAGAGAGATAGCAGAGAAATACGGAATCAGTATCGCACAAATTACCAGAGGATCTAATGCCCTTAAAGGATCAGATCCTCAATTTAAAGAGTTTTTACAAAAAGAGATCTGATCTTCTTTTGTAAAATACAAATAAGATTGAAAGTATTTGTATGCACGCGTTGTTAATGAACAAATATTCTGTTTTAGCAGTTTTGGTACGTAAGTATAGCTGCAGCATGCCATGCAAATCAGCTTTTCAAGCTGATTGCTTCCAAGATATTCAAAAATTCATCCTCTTACAGCGTGCCTGGCTTTCTTTTGAAAGCTGGCGCTTATCTACTTGGCGATAGGCCTAATTAAGAAGCCTTTTATTTGATTAAGAGATGTTCTTATAGAAGTAAGAGCGTCTTTTTTGCGCAGGATTATTCTGTCGCCAGTTTTTTCTATGATTTTAACACTATAATTTTATGGAGAAAAGATGTTCAAACATAAACATCCTTTTGGGGGAGCGTTCCTTCCCGAAGAACTATTAGCCCCTATACAGAATCTAAAAGCGGAATGGGAGATTCTCAAAACTCAGCAAAGTTTTTTATCTGAACTAGATTGTATTTTGAAAAACTATGCGGGGAGACAAACTCCTCTGACTGAAGTTAAGAATTTTGCTCGAGCTATTGATGGCCCTAGTATTTCTTAAACGCGAAGATCTTTTGCATACAGGAGCACATAAACTGAATAATGCTCTTGGTCAGTGTTTGCTTGCTAATATCTTGGGAAANCACGTGTTGTAGCTGAANNNGGTGCGGGNNNNCATGGAGTANNNNCAGCANAGCNNNTGNNATCTAGGATTAGATTGGNNNGNANCATGGGAGCAANGNNGNNAANNNNAANNCCAATNNNNNNANNGNNNTTTNAGGNCNNANTNNTNNNNNNNNAANGNNNNNGGGNACCAANNNGNNNTANNNNGNNNCANNNNGGGNNNNNNNNNNNTNNNCNNNTNNNNNNNNNNNNTNNANNTNNNNNNNCNNNNNNNNNNTTTTNNNNNNNANNNNNNNNNANNCANNNNNNNNNNNNNNAANNNNNNNNNN

>T3

NNNNNNTNNNNNNNGGNAGCAGTGGTATCAACGCAGAGTACATGGGCAAAAAGGTGCGATATGAAAAACCAAGAGNAGTCTGGCTGGCAAGCTTTTCTGACATTATGCTCTAAAATGCAAAAAGAAAAGTTTTTACAAGACCTTTTTTCGCTGTTTTTGTCTTTTGGCGAACGTAAAGATGTCGCTTCTCGCTATCATATCATTCGAGCTCTTTTAGAAGGGGAGCTCACTCAAAGAGAGATAGCAGAGAAATACGGAGTCAGTATCGCACAAATTACCAGAGGATCTAATGCCCTTAAAGGATCAGATCCTCAATTTAAAGAGTTTTTACAAAAAGAGATCTGATCTTCTTTTGTAAAATACAAATAAGATTGAAAGTATTTGTATGCACGCGTTGTTAATGAACAAATATTCTGTTTTAGCAGTTTTGGTACGTAAGTATAGCTGCAGCATGCCATGCAAATCAGCTTTTCAAGCTGATTGCTTCCAAGATATTCAAAAATTCATCCTCTTACAGCGTGCCTGGCTTTCTTTTGAAAGCTGGCGCTTATCTACTTGGCGATAGGCCTAATTAAGAAGCCTTTTATTTGATTAAGAGATGTTCTTATAGAAGTAAGAGCGTCTTTTTTGCGCAGGATTATTCTGTCGCCAGTTTTTTCTATGATTTTAACACTATAATTTTATGGAGAAAAGATGTTCAAACATAAACATCCTTTTGGGGGAGCGTTCCTTCCCGAAGAACTATTAGCCCCTATACAGAATCTAAAAGCGGAATGGGAGATTCTCAAAACTCAGCAAAGTTTTTTATCTGAACTAGATTGTATTTTGAAAAACTATGCGGGGAGACAAACTCCTCTGACTGAAGTTAAGAATTTTGCTCGAGCTATTGATGGCCCTAGAGTATTNCTAANCGCGAAGATTTNNNNNTACNGNNNCCNNNAANCCGANGNNTGCTCTTGGTCANNGTNNGCNNNCAANNNNNNNNNAAANNNCNTGTTGANNNNNNNNNGNTGNGNNAANCANGNCNNNNNANNGCNNNNNNGGNTGCTNNNNNGGNNNNNTNN

>T4

NNNNNNNNNNNNNNNGGGNAGCAGTGGTATCAACGCAGAGTGCATGGGCAAAAAGGTGCGATATGAAAAATCAAGAGNAGTCTGGCTGGCAAGCTTTTCTGACATTATGCTCTAAAATGCAAAAAGAAAAGTTTTTACAAGACCTTTTTTCGCTGTTTTTGTCTTTTGGCGAACGTAAAGATGTCGCTTCTCGCTATCATATCATTCGAGCTCTTTTAGAAGGGGAGCTCACTCAAAGAGAGATAGCAGAGAAATACGGAGTCAGTATCGCACAAATTACCAGAGGATCTAATGCCCTTAAAGGATCAGATCCTCAATTTAAAGAGTTTTTACAAAAAGAGATCTGATCTTCTTTTGTAAAATACAAATAAGATTGAAAGTATTTGTATGCACGCGTTGTTAATGAACAAATATTCTGTTTTAGCAGTTTTGGTACGTAAGTATAGCTGCAGCATGCCATGCAAATCAGCTTTTCAAGCTGATTGCTTCCAAGATATTCAAAAATTCATCCTCTTACAGCGTGCCTGGCTTTCTTTTGAAAGCTGGCGCTTATCTACTTGGCGATAGGCCTAATTAAGAAGCCTTTTATTTGATTAAGAGATGTTCTTATAGAAGTAAGAGCGTCTTTTTTGCGCAGGATTATTCTGTCGCCAGTTTTTTCTATGATTTTAACACTATAATTTTATGGAGAAAAGATGTTCAAACATAAACATCCTTTTGGGGGAGCGTTCCTTCCCGAAGAACTATTAGCCCCTATACAGAATCTAAAAGCGGAATGGGAGATTCTCAAAACTCAGCAAAGTTTTTTATCTGAACTAGATTGTATTTTGAAAAACTATGCGGGGAGACAAACTCCTCTGACTGAAGTTAAGAATTTTGCTCGAGCTATTGATGGCCCTAGAGTATTTCTTAAACGCGAAGATCTTTTGCATACAGGAGCACATAAACTGAATAATGCTCTTGGTCAGTGTTTGCTTGCTAATATCTTGGGAAAACACGTGTTGTAGCTGAAACAGGTGCGGGNNANCATGGAGTAGNNNNNNCNCAGCGTGGNNTATCTAGGANNNGNTTGGNNNGNNNNATGGGAGCAANNNNGGNNACGANNAANCCAANNNAANNAANNGCNNTTTNAGGNNNNNNNNNTTNNNGAACAANGNNNNNGGNNNNNANNNNNNNNTNNNNNNNNNNNNNNNNNNNNNNNNCCNNTNNNCCCNTNNNNNNNANNNNTNNNNNNTNNNTNNNNNNNNNNNAATTNNNNNNNNAAANNNNNNNNNNNGAANNNNNNNNTNNNAANNNNNNNNNNNNNNNNNNNNNNNNCNNGNNNNNNNNNNNTNN

>T5

NNNNNNANNTNNNNNNGGNNAGCAGTGGTATCAACGCAGAGTAGATGGGATTACAAAAAGGTGCGATATGAAAAATCANGAGGAGTCTGGCTGGCAAGCTTTTCTGACATTATGCTCTAAAATGCAAAAAGAAAAGTTTTTACAAGACCTTTTTTCGCTGTTTTTGTCTTTTGGCGAACGTAAAGATGTCGCTTCTCGCTATCATATCATTCGAGCTCTTTTAGGAGGGGAGCTCACTCAAAGAGAGATAGCAGAGAAATACGGAGTCAGTATCGCACAAATTACCAGAGGATCTAATGCCCTTAAAGGATCAGATCCTCAATTTAAAGAGTTTTTACAAAAAGAGATCTGATCTTCTTTTGTAAAATACAAATAAGATTGAAAGTATTTGTATGCACGCGTTGTTAATGAACAAATATTCTGTTTTAGCAGTTTTGGTACGTAAGTATAGCTGCAGCATGCCATGCAAATCAGCTTTTCAAGCTGATTGCTTCCAAGATATTCAAAAATTCATCCTCTTACAGCGTGCCTGGCTTTCTTTTGAAAGCTGGCGCTTATCTACTTGGCGATAGGCCTAATTAAGAAGCCTTTTATTTGATTAAGAGATGTTCTTATAGAAGTAAGAGCGTCTTTTTTGCGCAGGATTATTCTGTCGCCAGTTTTTTCTATGATTTTAACACTATAATTTTATGGAGAAAAGATGTTCAAACATAAACATCCTTTTGGGGGAGCGTTCCTTCCCGAAGAACTATTAGCCCCTATACAGAATCTAAAAGCGGAATGGGAGATTCTCAAAACTCAGCAAAGTTTTTTATCTGAACTAGATTGTATTTTGAAAAACTATGCGGGGAGACAAACTCCTCTGACTGAAGTTAAGAATTTTGCTCGAGCTATTGATGGCCCTAGAGTATTTCTTAAACGCGAAGATCTTTTGCATACAGGAGCACATAAACTGAATAATGCTCTTGNTCAGTGTTTGCTTGCTAANTATCTTGGGAAAACACGTGTTGTAGCTGAAACAGGTGCGGGACAACATGGAGTAGCNNNNGCANNNGNNNNNNNTTATCTAGGATTAGATTGGNNNNNAACNTGGGNAGCAANNNNNNGNNCNNNNNAAACCAANNNANNNAANNNNNNTTTNNAGGNNNNANNNNNTTNNGGAAAAANGNNNNNGGGNACNAAANNNGNTNNNNNNNNNNCNNNTNGGNNNCANNNNNTTNNNNCNNNNNNTTAGGNNNNNNTNNNNNTTCNTNCCNNNNNNNNNNTTNNTNNGNNTANNGNGNANNAANNNNNNNNNNNNNNNNNNNNNACNNN

>T6

NNNNNNTNCCNNNNGGNNAGCAGTGGTATCAACGCAGAGTACATGGGGTGCGATATGAAAAATCAAGAGGAGTCTGGCTGGCAAGCTTTTCTGACATTATGCTCTAAAATGCAAAAAGAAAAGTTTTTACAAGACCTTTTTTCGCTGTTTTTGTCTTTTGGCGAACGTAAAGATGTCGCTTCTCGCTATCATATCATTCGAGCTCTTTTAGAAGGGGAGCTCACTCAAAGAGAGATAGCAGAGAAATACGGAGTCAGTATCGCACAAATTACCAGAGGATCTAATGCCCTTAAAGGATCAGATCCTCAATTTAAAGAGTTTTTACAAAAAGAGATCTGATCTTCTTTTGTAAAATACAAATAAGATTGAAAGTATTTGTATGCACGCGTTGTTAATGAACAAATATTCTGTTTTAGCAGTTTTGGTACGTAAGTATAGCTGCAGCATGCCATGCAAATCAGCTTTTCAAGCTGATTGCTTCCAAGATATTCAAAAATTCATCCTCTTACAGCGTGCCTGGCTTTCTTTTGAAAGCTGGCGCTTATCTACTTGGCGATAGGCCTAATTAAGAAGCCTTTTATTTGATTAAGAGATGTTCTTATAGAAGTAAGAGCGTCTTTTTTGCGCAGGATTATTCTGTCGCCAGTTTTTTCTATGATTTTAACACTATAATTTTATGGAGAAAAGATGTTCAAACATAAACATCCTTTTGGGGGAGCGTTCCTTCCCGAAGAACTATTAGCCCCTATACAGAATCTAAAAGCGGAATGGGAGATTCTCAAAACTCAGCAAAGTTTTTTATCTGAACTAGATTGTATTTTGAAAAACTATGCGGGGAGACAAACTCCTCTGACTGAAGTTAAGAATTTTGCTCGAGCTATTGATGGCCCTAGAGTATTTCTTAAACGCGAAGATCTTTTGCATACAGGAGCACATAAACTGAATAATGCTCTTGGTCAGTGTTTGCTTGCTAAATATCTTGGGAAANCACGTGTTGTAGCTGAAACAGGTGCGGGNNNNCATGGAGTAGCNNNNNCANNNCNTGGNCTTNTCTAGGNNNNNNTTGGNNNNNAACATGGNAGCAAANNNGNNAACGNNGGNNCNAANNNNNNNAAANGCGCTTTTNAGGNNNNNNNNNTTNNNNAANNAANGNNTNNGNNNNNNNAANNNNNNNTNNCNNNNNNNNNANNNGGNNNNNNCNNNNTNCNNTNNNNNNNNNNNNNCGNNNTGGACTTCNNNNNNN

>T7

NNNNNNNTNCNNNNNGGGNNNCAGTGGTATCAACGCAGAGTACATGGGATTACAAAAAGGTGCGATATGAAAAATCAAGAGGAGTCTGGCTGGCAAGCTTTTCTGACATTATGCTCTAAAATGCAAAAAGAAAAGTTTTTACAAGACCTTTTTTCGCTGTTTTTGTCTTTTGGCGAACGTAAAGATGTCGCTTCTCGCTATCATATCATTCGAGCTCTTTTAGAAGGGGAGCTCACTCAAAGAGAGATAGCAGAGAAATACGGAGTCAGTATCGCACAAATTACCAGAGGATCTAATGCCCTTAAAGGATCAGATCCTCAATTTAAAGAGTTTTTACAAAAAGAGATCTGATCTTCTTTTGTAAAATACAAATAAGATTGAAAGTATTTGTATGCACGCGTTGTTAATGAACAAATATTCTGTTTTAGCAGTTTTGGTACGTAAGTATAGCTGCAGCATGCCATGCAAATCAGCTTTTCAAGCTGATTGCTTCCAAGATATTCAAAAATTCATCCTCTTACAGCGTGCCTGGCTTTCTTTTGAAAGCTGGCGCTTATCTACTTGGCGATAGGCCTAATTAAGAAGCCTTTTATTTGATTAAGAGATGTTCTTATAGAAGTAAGAGCGTCTTTTTTGCGCAGGATTATTCTGTCGCCAGTTTTTTCTATGATTTTAACACTATAATTTTATGGAGAAAAGATGTTCAAACATAAACATCCTTTTGGGGGAGCGTTCCNTCCCCGAANAACTATTAGCCCCTATACAGAANCTAAAAACGGAATGGGAGATTCTCAAAACTCAGCANAGNTTNTTTATCTGAACTAGATTGNNTTTTGAAAACTATGCTGGGNAGACAAAACTCCCTNTGGACTGAANNNNNNNAATTTTGCCTCTANCNTNTTGNTGNGNCCCNANANN

December 2 2017

>121

NNNNNNNNNNNNNTAGGNGCAGCAGTGGTATCNACGCAGAGTACATGGGCTATGATTTTAACACTATAATTTTATGGAGAAAAGATGTTCAAACATAAACATCCTTTTGGGGGAGCGTTCCTTCCCGAAGAACTATTAGCCCCTATACAGAATCTAAAAGCGGAATGGGAGATTCTCAAAACTCAGCAAAGTTTTTTATCTGAACTAGATTGTATTTTGAAAAACTATGCGGGGAGACAAACTCCTCTGACTGAAGTTAAGAATTTTGCTCGAGCTATTGATGGCCCTAGAGTATTTCTTAAACGCGAAGATCTTTTGCATACAGGAGCACATAAACTGAATAATGCTCTTGGTCAGTGTTTGCTTGCTAAATATCTTGGGAAAACACGTGTTGTAGCTGAAACAGGTGCGGGACAACATGGAGTAGCAACAGCAACAGCGTGTGCTTATCTAGGATTAGATTGTGTAGTATACATGGGAGCAAAAGATGTGGAACGACAGAAACCAAATGTAGAGAAAATGCGCTTTTTAGGTGCTGAAGTCGTTTCTGTAACAAAAGGATCTTGTGGACTCAAAGATGCAGTTAATCAAGCTCTACAAGATTGGGCAACAACACACTCATTTACTCACTATTGCTTAGGATCGGCCTTAGGACCTTTACCTTATCCCGATATCGTTCGATTTTTTCAGTCTGTTATAAGCGCTGANGTNACAGAGNNNNT

>122

NNNNNNNNNNNNNNNNGCAGCAGTGGTATCAACGCAGAGTACATGGGCTATGATTTTAACACTATAATTTTATGGAGAAAAGATGTTCAAACATAAACATCCTTTTGGGGGAGCGTTCCTTCCCGAAGAACTATTAGCCCCTATACAGAATCTAAAAGCGGAATGGGAGATTCTCAAAACTCAGCAAAGTTTTTTATCTGAACTAGATTGTATTTTGAAAAACTATGCGGGGAGACAAACTCCTCTGACTGAAGTTAAGAATTTTGCTCGAGCTATTGATGGCCCTAGAGTATTTCTTAAACGCGAAGATCTTTTGCATACAGGAGCACATAAACTGAATAATGCTCTTGGTCAGTGTTTGCTTGCTAAATATCTTGGGAAAACACGTGTTGTAGCTGAAACAGGTGCGGGACAACATGGAGTAGCAACAGCAACAGCGTGTGCTTATCTAGGATTAGATTGTGTAGTATACATGGGAGCAAAAGATGTGGAACGACAGAAACCAAATGTAGAGAAAATGCGCTTTTTAGGTGCTGAAGTCGTTTCTGTAACAAAAGGATCTTGTGGACTCAAAGATGCAGTTAATCAAGCTCTACAAGATTGGGCAACAACACACTCATTTACTCACTATTGCTTAGGATCGGCCTTAGGACCTTTACCTTATCCCGATATCGTTCGATTTTTTCAGTCTGTTATAAGCGCTGAAGTGAAAGAGCAAATCCATGCAGTTGCAGGAAGAGATCCTGATATTCTGATTGCATGTATCGGAGGTGGCTCCAACGCTATTGGATTTTTCCATCATTTTATCCCGAATCCAAAAGTCCAATTAATTGGAGTGGAAGGGGGAGGACTGGGCATTTCTTCAGGAAAACATGCNNCACGTTTTGCAANNGGGCGANTNGNANNATTCCNNAA

>123

NNNNNNNNNNNNNGGGCAGCAGTGGTATCAACGCAGAGTACATGGGCTATGATTTTAACACTATAATTTTATGGAGAAAAGATGTTCAAACATAAACATCCTTTTGGGGGAGCGTTCCTTCCCGAAGAACTATTAGCCCCTNTCAGAATCTAAAAGCGGAATGGGAGATTCTCAAACTCAGCAAGTTTTTTATCTGAACAGATTGATTTTGAANNCTATGCGGGGAGACAACTCCTCTGACTGAAGTTAAGAATTTTGCTCGAGCTATTGATGGCCCTAGAGTATTTCTTANACGCGAAGATCTTTTGCATACAGGAGCACATAAACTGAACAATGCTCTTGGACAGTGTTTGCTTGCTNNATATCTTNNNAAAANNCNNGTTGAANNTNGANATANACGNGGNATCTTNTNCATANCANNGCANNGCNGCTNTNNNNNNGATNNNNTNNGNNTCTCAANNCTNNNAACATCNANNNNNNANTTAACCNTACCAANNNNNCTCTTNGCATNANCNTCAGNATTTNCTNCCTANNTTACTCNTCTNNTNCNTTTCNACNANCCTNTATTACATTNNCNNNNTANNGNNNNGCNNNTTCTTCNNNNNNNNTNCANNACAAANNNNCTNTATACNNACNCTNNTNTATNNTTCTNNCNNANNTCACANNNCTCATNCNTNNTNNCTANNTC

>128

NNNNNNNNNNNNNGGGNAGCAGTGGTATCAACGGAGAGTACATGGGCTACTTGGCGATAGGCCTAATTAAGAAGCCTTTTATTTGATTAAGAGATGTTCTTATAGAAGTAAGAGCGTCTTTTTTGCGCAGGATTATTCTGTCGCCAGTTTTTTCTATGATTTTAACACTATAATTTTATGGAGAAAAGATGTTCAAACATAAACATCCTTTTGGGGGAGCGTTCCTTCCCGAAGAACTATTAGCCCCTATACAGAATCTAAAAGCGGAATGGGAGATTCTCAAAACTCAGCAAAGTTTTTTATCTGAACTAGATTGTATTTTGAAAAACTATGCGGGGAGACAAACTCCTCTGACTGAAGTTAAGAATTTTGCTCGAGCTATTGATGGCCCTAGAGTATTTCTTAAACGCGAAGATCTTTTGCATACAGGAGCACATAAACTGAATAATGCTCTTGGTCAGTGTTTGCTTGCTAAATATCTTGGGAAAACACGTGTTGTAGCTGAAACAGGTGCGGGACAACATGGAGTAGCAACAGCAACAGCGTGTGCTTATCTAGGATTAGATTGTGTAGTATACATGGGAGCAAAAGATGTGGAACGACAGAAACCAAATGTAGAGAAAATGCGCTTTTTAGGTGCTGAAGTCGTTTCTGTAACAAAAGGATCTTGTGGACTCAAAGATGCAGTTAATCAAGCTCTACAAGATTGGGCAACAACACACTCATTTACTCACTATTGCTTAGGATCGGCCTTAGGACCTTTACCTTATCCCGATATCGTTCGATTTTTTCAGTCTGTTATAAGCGCTGAAGTGAAAGAGCAAATCCATGCAGTTGCAGGAAGAGATCCTGATATTCTGATTGCATGTATCGGAGGTGGCTCCAACGCTATTGGATTTTTCCATCATTTTATCCCGAATCCAAAAGTCCAATTAATTGGAGTGGAAGGGGGNAGGACTGGGCATTTCTTCAGGAAAACATGCAGCACGTTTTGCAACAGGGCGACCTGGAGTATTCCACGAAGCTTGGCGTAATCATGGTCATAGCTGTTTCCTGTGTGAAATTGTTATCCGCTCNNAATTCCACACAACATACGAGCCGGAAGCNNAAANNNTAANGCCTGGNNNGCCTAATGNNNGAGCTAACTCANNTTAATTGNNTTGNNNTNACTGCCNNCTTTCCATNCGGNAANCNNNNCGGCNNNNNNNCTNNNTGAANCNGCCAA

>181

NNNNNNNNNNNNNGGGCAGCAGTGGTATCAACGCAGAGTACATGGGGTAGCATATATTTATGAAATGTTGTAATACTATAGCATTACAAAAAGGTGCGATATGAAAAATCAAGAGGAGTCTGGCTGGCAAGCTTTTCTGACATTATGCTCTAAAATGCAAAAAGAAAAGTTTTTACAAGACCTTTTTTCGCTGTTTTTGTCTTTTGGCGAACGTAAAGATGTCGCTTCTCGCTATCATATCATTCGAGCTCTTTTAGAAGGGGAGCTCACTCAAAGAGAGATAGCAGAGAAATACGGAGTCAGTATCGCACAAATTACCAGAGGATCTAATGCCCTTAAAGGATCAGATCCTCAATTTAAAGAGTTTTTACAAAAAGAGATCTGATCTTCTTTTGTAAAATACAAATAAGATTGAAAGTATTTGTATGCACGCGTTGTTAATGAACAAATATTCTGTTTTAGCAGTTTTGGTACGTAAGTATAGCTGCAGCATGCCATGCAAATCAGCTTTTCAAGCTGATTGCTTCCAAGATATTCAAAAATTCATCCTCTTACAGCGTGCCTGGCTTTCTTTTGAAAGCTGGCGCTTATCTACTTGGCGATAGGCCTAATTAAGAAGCCTTTTATTTGATTAAGAGATGTTCTTATAGAANTAAGAGCGACTTNNT

>182

NNNNNNNNNNNNNNNGGNAGCAGTGGTATCNACGCAGAGTACATGGGCTACTTGGCGATAGGCCTAATTAAGAAGCCTTTTATTTGATTAAGAGATGTTCTTATAGAAGTAAGAGCGTCTTTTTTGCGCAGGATTATTCTGTCGCCAGTTTTTTCTATGATTTTAACACTATAATTTTATGGAGAAAAGATGTTCAAACATAAACATCCTTTTGGGGGAGCGTTCCTTCCCGAAGAACTATTAGCCCCTATACAGAATCTAAAAGCGGAATGGGAGATTCTCAAAACTCAGCAAAGTTTTTTATCTGAACTAGATTGTATTTTGAAAAACTATGCGGGGAGACAAACTCCTCTGACTGAAGTTAAGAATTTTGCTCGAGCTATTGATGGCCCTAGAGTATTTCTTAAACGCGAAGATCTTTTGCATACAGGAGCACATAAACTGAATAATGCTCTTGGTCAGTGTTTGCTTGCTAAATATCTTGGGAAAACACGTGTTGTAGCTGAAACAGGTGCGGGGCAACATGGAGTAGCAACAGCAACAGCGTGTGCTTATCTAGGATTAGATTGTGTAGTATACATGGGAGCAAAAGATGTGGAACGACAGAAACCAAATGTAGAGAAAATGCGCTTTTTAGGTGCTGAAGTCGTTTCTGTAACAAAAGGATCTTGTGGACTCAAAGATGCAGTTAATCAAGCTCTACAAGATTGGGCAACAACACACTCATTTACTCACTATTGCTTAGGATCGGCCTTAGGACCTTTACCTTATCCCGATATCGTTCGATTTTTTCAGTCTGCTATAAGCGCTGAAGTGAAAGAGCAAATCCATGCAGTTGCAGGAAGAGATCCTGATATTCTGATTGCATGTATCGGAGGTGGCTCCAACGCTATTGGATTTTTCCATCATTTTATCCCGAATCCAAAAGTCCAATTAATTGGAGTGGAAGGGGGAGGACTGGGCNTTNNTTCAGGAAAACATGCCTTACNNTTTNGCAANAAANNNAANTGAAANNTTCCNNAAAANTNGNNNAA

>183

NNNNNNNNNNNNNNGCAGCAGTGGTATCAACGCAGAGTACATGGGGCCTTTTATTTGATTAAGAGATGTTCTTATAGAAGTAAGAGCGTCTTTTTTGCGCAGGATTATTCTGTCGCCAGTTTTTTCTATGATTTTAACACTATAATTTTATGGAGAAAAGATGTTCAAACATAAACATCCTTTTGGGGGAGCGTTCCTTCCCGAAGAACTATTAGCCCCTATACAGAATCTAAAAGCGGAATGGGAGATTCTCAAAACTCAGCAAAGTTTTTTATCTGAACTAGATTGTATTTTGAAAAACTATGCGGGGAGACAAACTCCTCTGACTGAAGTTAAGAATTTTGCTCGAGCTATTGATGGCCCTAGAGTATTTCTTAAACGCGAAGATCTTTTGCATACAGGAGCACATAAACTGAATAATGCTCTTGGTCAGTGTTTGCTTGCTAAATATCTTGGGAAAACACGTGTTGTAGCTGAAACAGGTGCGGGACAACATGGAGTAGCAACAGCAACAGCGTGTGCTTATCTAGGATTAGATTGTGTAGTATACATGGGAGCAAAAGATGTGGAACGACAGAAACCAAATGTAGAGAAAATGCGCTTTTTAGGTGCTGAAGTCGTTTCTGTAACAAAAGGATCTTGTGGACTCAAAGATGCAGTTAATCAAGCTCTACAAGATTGGGCAACAACACACTCATTTACTCACTATTGCTTAGGATCGGCCTTAGGACCTTTACCTTATCCCGATATCGTTCGATTTTTTCAGTCTGTTATAAGCGCTGAAGTGAAAGAGCAAATCCATGCAGTTGCAGGAAGAGATCCTGATATTCTGATTGCATGTATCGGAGGTGGCTCCAACGCTATTGGATTTTTCCATCATTTTATCCCGAATCCAAAAGTCCAATTAATTGGAGTGAAAGGGGGGAGGACTGGGCATTTCTTCAGGAAAACATGCAGCACGTTTTGCAACAGGGCGACCTGGAGTATTCCACGAAGCTTGGCGTAATCATGGTCATAGCTGTTTCCTGGGTGAAATTGTTATCCGCTCACAATTCCNCACAACATACGAGCCGGAAGCATAAAGTGTAAAGCCNGGGNTGCCTAATGAGTNNNCTAANNCNNNTTAATTGNNTTGNNNNCNNGNCCCGCTTTCCNNTCGGNAANCNNNNCCNGNCNNCNGNNTNAANNANNCCGCCNNCCGCCGGGGNAANGNCNTT

>185

NNNNNNNNNNNNGGGNAGCAGTGGTATCAACGCAGAGTACATGGGCTATGATTTTAACACTATAATTTTATGGAGAAAAGATGTTCAAACATAAACATCCTTTTGGGGGAGCGTTCCTTCCCGAAGAACTATTAGCCCCTATACAGAATCTAAAAGCGGAATGGGAGATTCTCAAAACTCAGCAAAGTTTTTTATCTGAACTAGATTGTATTTTGAAAAACTATGCGGGGAGACAAACTCCTCTGACTGAATTAAGAATTTTGCTCGAGCTATTGATGGCCCTAGAGTATTTCTTAAACGCGAAGATCTTTTGCATACAGGAGCACATAAACTGAATAATGCTCTTGGTCAGTGTTTGCTTGCTAAATATCTTGGGAAAACACGTGTTGTAGCTGAAACAGGTGCGGGACAACATGGAGTAGCAACAGCAACAGCGTGTGCTTATCTAGGATTAGATTGTGTAGTATACATGGGAGCNAAACATGTGGNNGACAGAAACCAAATGTAAGAAAATGCGCNNTTTANNTGCTGNAACNNNNGTNACANNGGATCTTGNGNNTCNNNTNNNNTTANCAGNNNNNANAATT

>186

NNNNNNNNNNNTAGGGCAGCAGTGGTATCAACGCAGAGTACATGGGTTGGCGATAGGCCTAATTAAGAAGCCTTTTATTTGATTAAGAGATGTTCTTATAGAAGTAAGAGCGTCTTTTTTGCGCAGGATTATTCTGTCGCCAGTTTTTTCTATGATTTTAACACTATAATTTTATGGAGAAAAGATGTTCAAACATAAACATCCTTTTGGGGGAGCGTTCCTTCCCGAAGAACTATTAGCCCCTATACAGAATCTAAAAGCGGAATGGGAGATTCTCAAAACTCAGCAAAGTTTTTTATCTGAACTAGATTGTATTTTGAAAAACTATGCGGGGAGACAAACTCCTCTGACTGAAGTTAAGAATTTTGCTCGAGCTATTGATGGCCCTAGAGTATTTCTTAAACGCGAAGATCTTTTGCATACAGGAGCACATAAACTGAATAATGCTCTTGGTCAGTGTTTGCTTGCTAAATATCTTGGGAAAACACGTGTTGTAGCTGAAACAGGTGCGGGACAACATGGAGTAGCAACAGCAACAGCGTGTGCTTATCTAGGATTAGATTGTGTAGTATACATGGGAGCAAAAGATGTGGAACGACAGAAACCAAATGTAGAGAAAATGCGCTTTNNAGGAGCTGAAGTCGTTTCTGAAACAAAAGGATCATGTGGACTCGAAGATGCNGNTAATCNNNCTCTACANNA

>188

NNNNNNNNNNNNNNNNGCAGCAGTGGTATCAACGCAGAGTACATGGGCTATGATTTTAACACTATAATTTTATGGAGAAAAGATGTTCAAACATAAACATCCTTTTGGGGGAGCGTTCCTTCCCGAAGAACTATTAGCCCCTATACAGAATCTAAAAGCGGAATGGGAGATTCTCAAAACTCAGCAAAGTTTTTTATCTGAACTAGATTGTATTTTGAAAAACTATGCGGGGAGACAAACTCCTCTGACTGAAGTTAAGAATTTTGCTCGAGCTATTGATGGCCCTAGAGTATTTCTTAAACGCGAAGATCTTTTGCATACAGGAGCACATAAACTGAATAATGCTCTTGGTCAGTGTTTGCTTGCTAAATATCTTGGGAAAACACGTGTTGTAGCTGAAACAGGTGCGGGACAACATGGAGTAGCAACAGCAACAGCGTGTGCTTATCTAGGATTAGATTGTGTAGTATACATGGGAGCAAAAGATGTGGAACGACAGAAACCAAATGTAGAGAAAATGCGCTTTTTAGGTGCTGAAGTCGTTTCTGTAACAAAAGGATCTTGTGGACTCAAAGATGCAGTTAATCAAGCTCTACAAGATTGGGCAACAACACACTCATTTACTCACTATTGCTTAGGATCGGCCTTAGGACCTTTACCTTATCCCGATATCGTTCGATTTTTTCAGTCTGTTATAAGCGCTGAAGTGAAAGAGCAAATCCATGCAGTTGCAGGAAGAGATCCTGATATTCTGATTGCATGTATCGGAGGTGGCTCCAACGCTATTGGATTTTTCCATCATTTTATCCCGAATCCAAAAGTCCAATTAATTGGAGTGGAANGGGGGAGGACTGGGCATTTCTTCAGGAAAACATGCAGCACGTTTTGCAACAGGGCGACCTGGAGTATTCCACGAAGCTTGGCGTAATCATGGTCATAGCTGTTTCCTGTGTGAAATTGTTATCCGCTCACAATTCCACACAACATACGAGCCGGAAGCATAAAGTGTAAAGCCNGGGGTGCCTAANGANNGAGCTAACTCANATTAATTGCGTTGCGCTCACTGCCCNNTTTCCAGTCGGGAAACCTGNCCTGCNANNTGCATTAATGNNNCGGCCAACNNNNGGGNANNGGCGGTTNNNNGATTGGGNNNNNTTCNNCTTCCNCNNNNNNTGANCCCCNTNNNNNNGGNNCTTNNCNNNNGCNNAGNC

>189

NNNNNNNNNNNNNAGGGCAGCAGTGGTATCAACGCAGAGTACATGGGGTAGCATATATTTATGAAATGTTGTAATACTATAGCATTACAAAAAGGTGCGATATGAAAAATCAAGAGGAGTCTGGCTGGCAAGCTTTTCTGACATTATGCTCTAAAATGCAAAAAGAAAAGTTTTTACAAGACCTTTTTTCGCTGTTTTTGTCTTTTGGCGAACGTAAAGATGTCGCTTCTCGCTATCATATCATTCGAGCTCTTTTAGAAGGGGAGCTCACTCAAAGAGAGATAGCAGAGAAATACGGAGTCAGTATCGCACAAATTACCAGAGGATCTAATGCCCTTAAAGGATCAGATCCTCAATTTAAAGAGTTTTTACAAAAAGAGATCTGATCTTCTTTTGTAAAATACAAATAAGATTGAAAGTATTTGTATGCACGCGTTGTTAATGAACAAATATTCTGTTTTAGCAGTTTTGGTACGTAAGTATAGCTGCAGCATGCCATGCAAATCAGCTTTTCAAGCTGATTGCTTCCAAGATATTCAAAAATTCATCCTCTTACAGCGTGCCTGGCTTTCTTTTGAAAGCTGGCGCTTATCTACTTGGCGATAGGCCTAATTAANAAGCCTTTTATTTGGANNACCANANGTTNCTTAAAAAANTAANANNGNNNNTNNNNNNNNCNNNA

>1811

NNNNNNNNNNNNNNNNGGGCAAGCAGTGGTATCAACGCAGAGTACATGGGATTACAAAAAGGTGCGATATGAAAAATCAAGAGGAGTCTGGCTGGCAAGCTTTTCTGACATTATGCTCTAAAATGCAAAAAGAAAAGTTTTTACAAGACCTTTTTTCGCTGTTTTTGTCTTTTGGCGAACGTAAAGATGTCGCTTCTCGCTATCATATCATTCGAGCTCTTTTAGAAGGGGAGCTCACTCAAAGAGAGATAGCAGAGAAATACGGAGTCAGTATCGCACAAATTACCAGAGGATCTAATGCCCTTAAAGGATCAGATCCTCAATTTAAAGAGTTTTTACAAAAAGAGATCTGATCTTCTTTTGTAAAATACAAATAAGATTGAAAGTATTTGTATGCACGCGTTGTTAATGAACAAATATTCTGTTTTAGCAGTTTTGGTACGTAAGTATAGCTGCAGCATGCCATGCAAATCAGCTTTTCAAGCTGATTGCTTCCAAGATATTCAAAAATTCATCCTCTTACAGCGTGCCTGGCTTTCTTTTGAAAGCTGGCGCTTATCTACTTGGCGATAGGCCTAATTAAGAAGCCTTTTATTTGATTAAGAGATGTTCTTATAGAAGTAAGAGCGTCTTTTTTGCGCAGGATTATTCTGTCGCCAGTTTTTTCTATGATTTTAACACTATAATTTTATGGAGAAAAGATGTTCAAACATAAACATCCTTTTGGGGGAGCGTTCCTTCCCGAAGAACTATTAGCCCCTATACAGAATCTAAAAGCGGAATGGGAGATTCTCAAAACTCAGCAAAGTTTTTTATCTGAACTAGATTGTATTTTGAAAAACTATGCGGGGAGACAAACTCCTCTGACTGAAGTTAAGAATTTTGCTCGAGCTATTGATGGCCCTAGAGTATTTCTTAAACGCGAAGATCTTTTGCATACAGGAGCACATAAACTGAATAATGCTCTTGGTCAGTGTTTGCTTGCTAAATATCTTGGGAAAACACGTGTTGTAGCTGAAACAGGTGCGGGACAACATGGAGTAGCAACAGCAACAGCNNGTGCTTATCTAGGATTAGATTGGGTAGTNTACATGGGAGCAAAAGANNTGGAACGACCGAAACCAAATNNANNGAAANTGNNNTTTTNAGGNGCTGAA

>1812

NNNNNNNNNNNNNNNNNGCAGCAGTGGTATCAACGCAGAGTACATGGGATTACAAAAAGGTGCGATATGAAAAATCAAGAGGAGTCTGGCTGGCAAGCTTTTCTGACATTATGCTCTAAAATGCAAAAAGAAAAGTTTTTACAAGACCTTTTTTCGCTGTTTTTGTCTTTTGGCGAACGTAAAGATGTCGCTTCTCGCTATCATATCATTCGAGCTCTTTTAGAAGGGGAGCTCACTCAAAGAGAGATAGCAGAGAAATACGGAGTCAGTATCGCACAAATTACCAGAGGATCTAATGCCCTTAAAGGATCAGATCCTCAATTTAAAGAGTTTTTACAAAAAGAGATCTGATCTTCTTTTGTAAAATACAAATAAGATTGAAAGTATTTGTATGCACGCGTTGTTAATGAACAAATATTCTGTTTTAGCAGTTTTGGTACGTAAGTATAGCTGCAGCATGCCATGCAAATCAGCTTTTCAAGCTGATTGCTTCCAAGATATTCAAAAATTCATCCTCTTACAGCGTGCCTGGCTTTCTTTTGAAAGCTGGCGCTTATCTACTTGGCGATAGGCCTAATTAAGAAGCCTTTTATTTGATTAAGAGATGTTCTTATAGAAGTAAGAGCGTCTTTTTTGCGCAGGATTATTCTGTCGCCAGTTTTTTCTATGATTTTAACACTATAATTTTATGGAGAAAAGATGTTCAAACATAAACATCCTTTTGGGGGAGCGTTCCTTCCCGAAGAACTATTAGCCCCTATACAGAATCTAAAAGCGGAATGGGAGATTCTCAAAACTCAGCAAAGTTTTTTATCTGAACTAGATTGTATTTTGAAAAACTATGCGGGGAGACAAACTCCTCTGACTGAAGTTAAGAATTTTGCTCGAGCTATTGATGGCCCTAGAGTATTTCTTAAACGCGAAGATCTTTTGCATACAGGAGCACATAAACTGAATAATGCTCTTGGTCAGTGTTTGCTTGCTAAATATCTTGGGGAAAACACGTGTTGTAGCTGAAACAGGTGCGGGACAACATGGAGTAGCAACAGCAACAGCNNNNGCTTATCTAGGATTAGATTGGNTAGNNTACATGGGNAGCAAANGANNNGGAACNNNNNAANCCAATG

>B1

NNNNNNNNNNNTAGGGCAGCAGTGGTATCAACGCAGAGTACATGGGGTATCAACGCAGAGTACATGGGATCCTTTTGGGGGAGCGTTCCTTCCCGAAGAACTATTAGCCCCTATACAGAATCTAAAAGCGGAATGGGAGATTCTCAAAACTCAGCAAAGTTTTTTATCTGAACTAGATTGTATTTTGAAAAACTATGCGGGGAGACAAACTCCTCTGACTGAAGTTAAGAATTTTGCTCGAGCTATTGATGGCCCTAGAGTATTTCTTAAACGCGAAGATCTTTTGCATACAGGAGCACATAAACTGAATAATGCTCTTGGTCAGTGTTTGCTTGCTAAATATCTTGGGAAAACACGTGTTGTAGCTGAAACAGGTGCGGGACAACATGGAGTAGCAACAGCAACAGCGTGTGCTTATCTAGGATTAGATTGTGTAGTATACATGGGAGCAAAAGATGTGGAACGACAGAAACCAAATGTAGAGAAAATGCGCTTTTTAGGTGCTGAAGTCGTTTCTGTAACAAAAGGATCTTGTGGACTCAAAGATGCAGTTAATCAAGCTCTACAAGATTGGGCAACAACACACTCATTTACTCACTATTGCTTAGGATCGGCCTTAGGACCTTTACCTTATCCCGATATCGTTCGATTTTTTCAGTCTGTTATAAGCGCTGAAGTGAAAGAGCAAATCCATGCAGTNGCAGGAAGAGATCCTGATATNCTGATTGCATGNATCNGAGGTGGCTCCAACGCCTTTGGNAATTNTTACATCATTTTATC

>B2

NNNNNNNNNNNNNGGGCAGCAGTGGTATCAACGCAGAGTACATGGGTATAATTTTATGGAGAAAAGATGTTCAAACATAAACATCCTTTTGGGGGAGCGTTCCTTCCCGAAGAACTATTAGCCCCTATACAGAATCTAAAAGCGGAATGGGAGATTCTCAAAACTCAGCAAAGTTTTTTATCTGAACTAGATTGTATTTTGAAAAACTATGCGGGGAGACAAACTCCTCTGACTGAAGTTAAGAATTTTGCTCGAGCTATTGATGGCCCTAGAGTATTTCTTAAACGCGAAGATATTTTGCATACAGGAGCACATAAACTGAATAATGCTCTTGGTCAGTGTTTGCTTGCTAAATATCTTGGGAAAACACGTGTTGTAGCTGAAACAGGTGCGGGACAACATGGAGTAGCAACAGCAACAGCGTGTGCTTATCTAGGATTAGATTGTGTAGTATACATGGGAGCAAAAGATGTGGAACGACAGAAACCAAATGTAGAGAAAATGCGCTTTTTAGGTGCTGAAGTCGTTTCTGTAACAAAAGGATCTTGTGGACTCAAAGATGCAGTTAATCAAGCTCTACAAGATTGGGCAACAACACACTCATTTACTCACTATTGCTTAGGATCGGCCTTAGGACCTTTACCTTATCCCGATATCGTTCGATTTTTTCAGTCTGTTATAAGCGCTGAAGTGAAAGAGCAAATCCATGCAGTTGCAGGAAGAGATCCTGATATTCTGATTGCATGTATCGGAGGTGGCTCCAACGCTATTGGATTTTTCCATCATTTTATCCCGAATCCAAAAGTCCAATTAATTGGAGTGGAAGGGGGAGGACTGGGCATTTCTTCAGGANAACATGCAGCACGTTTTGCAACAGGGCGACCTGGAGTATTCCACGAAGCTTGGCGTTANNCTGGNNNTTAGCTGTTTC

>B3

NNNNNNNNNNNNNGGGCAGCAGTGGTATCAACGCAGAGTACATGGGCTACTTGGCGATAGGCCTAATTAAGAAGCCTTTTATTTGATTAAGAGATGTTCTTATAGAAGTAAGAGCGTCTTTTTTGCGCAGGATTATTCTGTCGCCAGTTTTTTCTATGATTTTAACACTATAATTTTATGGAGAAAAGATGTTCAAACATAAACATCCTTTTGGGGGAGCGTTCCTTCCCGAAGAACTATTAGCCCCTATACAGAATCTAAAAGCGGAATGGGAGATTCTCAAAACTCAGCAAAGTTTTTTATCTGAACTAGATTGTATTTTGAAAAACTATGCGGGGAGACAAACTCCTCTGACTGAAGTTAAGAATTTTGCTCGAGCTATTGATGGCCCTAGAGTATTTCTTAAACGCGAAGATCTTTTGCATACAGGAGCACATAAACTGAATAATGCTCTTGGTCAGTGTTTGCTTGCTAAATATCTTGGGAAAACACGTGTTGTAGCTGAAACAGGTGCGGGACAACATGGAGTAGCAACAGCAACAGCGTGTGCTTATCTAGGATTAGATTGTGTAGTATACATGGGAGCAAAAGATGTGGAACGACAGAAACCAAATGTAGAGAAAATGCGCTTTTTAGGTGCTGAAGTCGTTTCTGTAACAAAAGGATCTTGTGGACTCAAAGATGCAGTTAATCAAGCTCTACAAGATTGGGCAACAACACACTCATTTACTCACTATTGCTTAGGATCGGCCTTAGGACCTTTACCTTATCCCGATATCGTTCGATTTTTTCAGTCTGTTATAAGCGCTGAAGTGAAAGAGCAAATCCATGCAGTTGCAGGAAGAGATCCTGATATTCTGATTGCATGTATCGGAGGTGGCTCCAACGCTATTGGATTTTTCCATCATTTTATCCCGAATCCAAAAGTCCANTTAATTGGAAGTGGAAAGGGGGAGGACCGGGTCATTTCCTCNGGNNNANNNNG

>B7

NNNNNNNNNNNNNNGGCAGCAGTGGTATCAACGCAGAGTACGTGGGTTAGCAGTTTTGGTACGTAAGTATAGCTGCAGCATGCCATGCAAATCAGCTTTTCAAGCTGATTGCTTCCAAGATATTCAAAAATTCATCCTCTTACAGCGTGCCTGGCTTTCTTTTGAAAGCTGGCGCTTATCTACTTGGCGATAGGCCTAATTAAGAAGCCTTTTATTTGATTAAGAGATGTTCTTATAGAAGTAAGAGCGTCTTTTTTGCGCAGGATTATTCTGTCGCCAGTTTTTTCTATGATTTTAACACTATAATTTTATGGAGAAAAGATGTTCAAACATAAACATCCTTTTGGGGGAGCGTTCCTTCCCGAAGAACTATTAGCCCCTATACAGAATCTAAAAGCGGAATGGGAGATTCTCAAAACTCAGCAAAGTTTTTTATCTGAACTAGATTGTATTTTGAAAAACTATGCGGGGAGACAAACTCCTCTGACTGAAGTTAAGAATTTTGCTCGAGCTATTGATGGCCCTAGAGTATTTCTTAAACGCGAAGATCTTTTGCATACAGGAGCACATAAACTGAATAATGCTCTTGGTCAGTGTTTGCTTGCTAAATATCTTGGGAAAACACGTGTTGTAGCTGAAACAGGTGCGGGACAACATGGAGTAGCAACAGCAACAGCGTGTGCTTATCTAGGATTAGATTGTGTAGTATACATGGGAGCAAAAGATGTGGAACGACAGAAACCAAATGTAGAGAAAATGCGCTTTTTAGGTGCTGAAGTCGTTTCTGTAACAAAAGGATCTTGTGGACTCAAAGATGCAGTTAATCAAGCTCTACAAGATTGGGCAACAACACACTCATTTACTCACTATTGCTTAGGATCGGCCTTAGGACCTTTACCTTATCCCGATATCGTTCGATTTTTTCAGTCTGTTATAAGCGCTGAAGTGAAAGAGCAAATCCATGCAGTTGCAGGAAGAGATCCTGATATTCTGATTGCATGTATCGGAGGTGGCTCCAACGCTATTGGATTTTTCCATCATTTTATCCCGAATCCAAAGTCCANTAATTGGANGGNAAANGGGGNAGGANNNGGGCATTTCTTCAGGAAAANNTGCAGCACGTTTTGCAACAGGNCGACCTGGAANNTTCCCNGAAGNTT

>W12

NNNNNNNTNNNNNNNNNGGCAGCAGTGGTATCAACGCAGAGTACATGGGGATTACGCCAAGCTTAGCTCGTAACGCCTCTTCATCGGTGGCTGATTACGCCAAGCTTAGCTCGTAACGCCTCTTCATCGGTGGCTCTAATACGACTCACTATAGGGCAAGCAGTGGTATCAACGCAGAGTACATGGGGATTACGCCAAGCTTAGCTCGTAACGCCTCTTCATCGGTGGCTGATTACGCCAAGCTTAGCTCGTAACGCCTCTTCATCGGTGGCTCTAATACGACTCACTATAGGGCAAGCAGTGGTATCAACGCAGAGTACATGGGGATTACACCAAGCTTAGCTCGTAACGCCTCTTCNTCGGTGGCTGATTACGCCAAGCTTANCTCGTAACGCCTCTTCATCGGTGGCTCTAATAAGACTCACTATAGGGGAAGCANTGGTATCAACACAGAGTACATGGGTACCTTAGGATCTAATGGCCTTAAAAGATCACATCCTCTAGTTACANAATGNGCGCANN

January 5 2018

>121

NNNNNNNNNNNNANGGCAGCAGTGGTATCAACGCAGAGTACATGGGCTACTTGGCGATAGGCCTAATTAAGAAGCCTTTTATTTGATTAAGAGATGTTCTTATAGAAGTAAGAGCGTCTTTTTTGCGCAGGATTATTCTGTCGCCAGTTTTTTCTATGATTTTAACACTATAATTTTATGGAGAAAAGATGTTCAAACATAAACATCCTTTTGGGGGAGCGTTCCTTCCCGAAGAACTATTAGCCCCTATACAGAATCTAAAAGCGGAATGGGAGATTCTCAAAACTCAGCAAAGTTTTTTATCTGAACTAGATTGTATTTTGAAAAACTATGCGGGGAGACAAACTCCTCTGACTGAAGTTAAGAATTTTGCTCGAGCTATTGATGGCCCTAGAGTATTTCTTAAACGCGAAGATCTTTTGCATACAGGAGCACATAAACTGAATAATGCTCTTGGTCAGTGTTTGCTTGCTAAATATCTTGGGAAAACACGTGTTGTAGCTGAAACAGGTGCGGGACAACATGGAGTAGCAACAGCAACAGCGTGTGCTTATCTAGGATTAGATTGTGTAGTATACATGGGAGCAAAAGATGTGGAACGACAGAAACCAAATGTAGAGAAAATGCGCTTTTTAGGTGCTGAAGTCGTTTCTGTAACAAAAGGATCTTGTGGACTCAAAGATGCAGTTAATCAAGCTCTACAAGATTGGGCAACAACACACTCATTTACTCACTATTGCTTAGGATCGGCCTTAGGACCTTTACCTTATCCCGATATCGTTCGATTTTTTCAGTCTGTTATAAGCGCTGAAGTGAAAGAGCAAATCCATGCAGTTGCAGGAAGAGATCCTGATATTCTGATTGCATGTATCGGAGGTGGCTCCAACGCTATTGGATTTTTCCATCATTTTATCCCGAATCCAAAAGTCCAATTAATTGGGAGTGGAAAGGGGGAAGGANTGGGCATTTCTTCNGGAAAACATGCAGCACGTTTTGCAACAGGGCGACCTGGANNATTCNNCGAAGCTTGGCCGAAATCATGGNNNNNNCCTGTTTCCCGNNGNNAAATNGNTTNNCCGNNNNCNNTT

>122

NNNNNNNNNNNNNTNGGGNAGCAGTGGTATCAACGCAGAGTACATGGGCTATGATTTTAACACTATAATTTTATGGAGAAAAGATGTTCAAACATAAACATCCTTTTGGGGGAGCGTTCCTTCCCGAAGAACTATTAGCCCCTATACAGAATCTAAAAGCGGAATGGGAGATTCTCAAAACTCAGCAAAGTTTTTTTATCTGAACTAGATTGTATTTTGAAAAACTATGCGGGGAGACAAACTCCTCTGACTGAAGTTAAGAATTTTGCTCGAGCTATTGATGGCCCTAGAGTATTTCTTAAACGCGAAGATCTTTTGCATACAGGAGCACATAAACTGAATAATGCTCTTGGTCAGTGTTTGCTTGCTAAATATCTTGGGAAAACACGTGTTGTAGCTGAAACAGGTGCGGGACAACATGGAGTAGCAACAGCAACAGCGTGTGCTTATCTAGGATTAGATTGTGTAGTATACATGGGAGCAAAAGATGTGGAACGACAGAAACCAAATGTAGAGAAAATGCGCTTTTTAGGTGCTGAAGTCGTTTCTGTAACAAAAGGATCTTGTGGACTCAAAGATGCAGTTAATCAAGCTCTACAAGATTGGGCAACAACACACTCATTTACTCACTATTGCTTAGGATCGGCCTTAGGACCTTTACCTTATCCCGATATCGTTCGATTTTTTCAGTCTGTTATAAGCGCTGAAGTGAAAGAGCAAATCCATGCAGTTGCAGGAAGAGATCCTGATATTCTGATTGCATGTATCGGAGGTGGCTCCAACGCTATTGGATTTTTCCATCATTTTATCCCGAATCCAAAAGTCCAATTAATTGGAGCGGAAGGGGGAGGACTGGGCATTTCTTCAGGAAAACATGCAGCACGTTTTGCAACAGGGCGACCTGGAGTATTCCACGAAGCTTGGCGTAATCATGGTCATAGCTGTTTCCTGTGTGAAATTGTTATCCGCTCACAATTCCACACAACATACGAGCCGGAAGCATAAAGTGTAAAGCCTGGGGTGCCTAATGAGTGAGCTAACTCACATTAATTGCGTTGCGCTCACTGCCCGCTTTCCAGTCGGGAAACCTGTCNNGCCAC

>129

NNNNNNNNNNNNTNNNNNAGCAGTGGTATCAACGCAGAGTACATGGGCTACTTGGCGATAGGCCTAATTAAGAAGCCTTTTATTTGATTAAGAGATGTTCTTATAGAAGTAAGAGCGTCTTTTTTGCGCAGGATTATTCTGTCGCCAGTTTTTTCTATGATTTTAACACTATAATTTTATGGAGAAAAGATGTTCAAACATAAACATCCTTTTGGGGGAGCGTTCCTTCCCGAAGAACTATTAGCCCCTATACAGAATCTAAAAGCGGAATGGGAGATTCTCAAAACTCAGCAAAGTTTTTTATCTGAACTAGATTGTATTTTGAAAAACTATGCGGGGAGACAAACTCCTCTGACTGAAGTTAAGAATTTTGCTCGAGCTATTGATGGCCCTAGAGTATTTCTTAAACGCGAAGATCTTTTGCATACAGGAGCACATAAACTGAATAATGCTCTTGGTCAGTGTTTGCTTGCTAAATATCTTGGGAAAACACGTGTTGTAGCTGAAACAGGTGCGGGACAACATGGAGTAGCAACAGCAACAGCGTGTGCTTATCTAGGATTAGATTGTGTAGTATACATGGGAGCAAAAGATGTGGAACGACAGAAACCAAATGTAGAGAAAATGCGCTTTTTAGGTGCTGAAGTCGTTTCTGTAACAAAAGGATCTTGTGGACTCAAAGATGCAGTTAATCAAGCTCTACAAGATTGGGCAACAACACACTCATTTACTCACTATTGCTTAGGATCGGCCTTAGGACCTTTACATTATCCCGATATCGTTCGATTTTTTCAGTCTGTTATAAGCGCTGAAGTGAAAGAGCAAATCCATGCAGTTGCAGGAAGAGATCCTGATATTCTGATTGCATGTATCGGAGGTGGCTCCAACGCTATTGGATTTTTCCATCATTTTATCCCGAATCCAAAAGTCCAATTAATTGGAGTGGAAGGGGGNAGGACTGGGCATTTCTTCAGGAAAACATGCAGCACGTTTTGCAACAGGGCGACCTGGAATATTCCACGAAGCTTGGCGTAATCATGGTCATAGCTGTTTCCNGNNNNAAATTGTTATCCGCTCACAATTCCNNANAANNNNCGAGCCGGAANCNNAAAG

>185

NNNNNNNNNNNNNNTAGGGCAGCAGTGGTATCAACGCAGAGTACATGGGTCATCCTCTTACAGCGTGCCTGGCTTTCTTTTGAAAGCTGGCGCTTATCTACTTGGCGATAGGCCTAATTAAGAAGCCTTTTATTTGATTAAGAGATGTTCTTATAGAAGTAAGAGCGTCTTTTTTGCGCAGGATTATTCTGTCGCCAGTTTTTTCTATGATTTTAACACTATAATTTTATGGAGAAAAGATGTTCAAACATAAACATCCTTTTGGGGGAGCGTTCCTTCCCGAAGAACTATTAGCCCCTATACAGAATCTAAAAGCGGAATGGGAGATTCTCAAAACTCAGCAAAGTTTTTTATCTGAACTAGATTGTATTTTGAAAAACTATGCGGGGAGACAAACTCCTCTGACTGAAGTTAAGAATTTTGCTCGAGCTATTGATGGCCCTAGAGTATTTCTTAAACGCGAAGATCTTTTGCATACAGGAGCACATAAACTGAATAATGCTCTTGGTCAGTGTTTGCTTGCTAAATATCTTGGGAAAACACGTGTTGTAGCTGAAACAGGTGCGGGACAACATGGAGTAGCAACAGCAACAGCGTGTGCTTATCTAGGATTAGATTGTGTAGTATACATGGGAGCAAAAGATGTGGGACGACAGAAACCAAATGTAGAGAAAATGCGCTTTTTAGGTGCTGAAGTCGTTTCTGTAACAAAAGGATCTTGTGGACTCAAAGATGCAGTTAATCAAGCTCTACAAGATTGGGCAACAACACACTCATTTACTCACTATTGCTTAGGATCGGCCTTAGGACCTTTACCTTATCCCGATATCGTTCGATTTTTTCAGTCTGTTATAAGCGCTGAAGTGAAAGAGCAAATCCATGCAGTTGCAGGAAGAGATCCTGATATTCTGATTGCATGTATCGGAGGTGGCTCCAACGCTATTGGATTTTTCCATCATTTTATCCCGAATCCAAAAGTCCAATTAATTGGAGTGGAAGGGGGAGGACTGGGCATTTCTTCAGGAAAACATGCAGCACGTTTTGCAACAGGGCGACCTGGAGTATTCCACGAAGCTTGGNCGTAATCCTGGGCNNANGCTGTTT

>189

NNNNNNNNNNNNNNNATNGGGCAGCAGTGGTATCAACGCAGAGTACATGGGCTACTTGGCGATAGGCCTAATTAAGAAGCCTTTTATTTGATTAAGAGATGTTCTTATAGAAGTAAGAGCGTCTTTTTTGCGCAGGATTATTCTGTCGCCAGTTTTTTCTATGATTTTAACACTATAATTTTATGGAGAAAAGATGTTCAAACATAAACATCCTTTTGGGGGAGCGTTCCTTCCCGAAGAACTATTAGCCCCTATACAGAATCTAAAAGCGGAATGGGAGATTCTCAAAACTCAGCAAAGTTTTTTATCTGAACTAGATTGTATTTTGAAAAACTATGCGGGGAGACAAACTCCTCTGACTGAAGTTAAGAATTTTGCTCGAGCTATTGATGGCCCTAGAGTATTTCTTAAACGCGAAGATCTTTTGCATACAGGAGCACATAAACTGAATAATGCTCTTGGTCAGTGTTTGCTTGCTAAATATCTTGGGAAAACACGTGTTGTAGCTGAAACAGGTGCGGGACAACATGGAGTAGCAACAGCAACAGCGTGTGCTTATCTAGGATTAGATTGTGTAGTATACATGGGAGCAAAAGATGTGGAACGACAGAAACCAAATGTAGAGAAAATGCGCTTTTTAGGTGCTGAAGTCGTTTCTGTAACAAAAGGATCTTGTGGACTCAAAGATGCAGTTAATCAAGCTCTACAAGATTGGGCAACAACACACTCATTTACTCACTATTGCTTAGGATCGGCCTTAGGACCTTTACCTTATCCCGATATCGTTCGATTTTTTCAGTCTGTTATAAGCGCTGAAGTGAAAGAGCAAATCCATGCAGTTGCAGGAAGAGATCCTGATATTCTGATTGCATGTATCGGAGGTGGCTCCAACGCTATTGGATTTTTCCATCATTTTATCCCGAATCCAAAAGTCCAATTAATTGGANTGGAAAGGGGGGAGGACTGGNNNNTNTCTTNNGGNAAANNTGNNAGGNNNTTTTTNNNNNA

>BPD1

NNNNNNNNNNNNNNNNNNAGCAGTGGTATCAACGCAGAGTACATGGGTCTGTCGCCAGTTTTTTCTATGATTTTAACACTATAATTTTATGGAGAAAAGATGTTCAAACATAAACATCCTTTTGGGGGAGCGTTCCTTCCCGAAGAACTATTAGCCCCTATACAGAATCTAAAAGCGGAATGGGAGATTCTCAAAACTCAGCAAAGTTTTTTATCTGAACTAGATTGTATTTTGAAAAACTATGCGGGGAGACAAACTCCTCTGACTGAAGTTAAGAATTTTGCTCGAGCTATTGATGGCCCTAGAGTATTTCTTAAACGCGAAGATCTTTTGCATACAGGAGCACATAAACTGAATAATGCTCTTGGTCAGTGTTTGCTTGCTAAATATCTTGGGAAAACACGTGTTGTAGCTGAAACAGGTGCGGGACAACATGGAGTAGCAACAGCAACAGCGTGTGCTTATCTAGGATTAGATTGTGTAGTATACATGGGAGCAAAAGATGTGGAACGACAGAAACCAAATGTAGAGAAAATGCGCTTTTTAGGTGCTGAAGTCGTTTCTGTAACAAAAGGATCTTGTGGACTCAAAGATGCAGTTAATCAAGCTCTACAAGATTGGGCAACAACACACTCATTTACTCACTATTGCTTAGGATCGGCCTTAGGACCTTTACCTTATCCCGATATCGTTCGATTTTTTCAGTCTGTTATAAGCGCTGAAGTGAAAGAGCAAATCCATGCAGTTGCAGGAAGAGATCCTGATATTCTGATTGCATGTATCGGAGGTGGCTCCAACGCTATTGGATTTTTCCATCATTTTATCCCGAATCCAAAAGTCCAATTAATTGGAGTGGAAGGGGGGAAGGACTGGGGCATTTCTTCAGGAAAACATGCAGCACGTTTTGCAACAGGGCGACCTGGAGTATTCCACGAAGCTTGGCGTAATCATGGTCATAGCTGTTTCCTGTGTGAAATTNTTATCCGCTCACAATTCCACACAACATACGAGCCCGGAAGCATAAAGTGTAAAGCCTGGGGNNGCCTAATGNNTTGAGCTAACTCCAATTAATNGGNNTNGNNNTCCCTGGCC

>BPD2

NNNNNNNNNNNNNNNTAGGGCAGCAGTGGTATCAACGCAGAGTACATGGGCTATGATTTTAACACTATAATTTTATGGAGAAAAGATGTTCAAACATAAACATCCTTTTGGGGGAGCGTTCCTTCCCGAAGAACTATTAGCCCCTATACAGAATCTAAAAGCGGAATGGGAGATTCTCAAAACTCAGCAAAGTTTTTTATCTGAACTAGATTGTATTTTGAAAAACTATGCGGGGAGACAAACTCCTCTGACTGAAGTTAAGAATTTTGCTCGAGCTATTGATGGCCCTAGAGTATTTCTTAAACGCGAAGATCTTTTGCATACAGGAGCACATAAACTGAATAATGCTCTTGGTCAGTGTTTGCTTGCTAAATATCTTGGGAAAACACGTGTTGTAGCTGAAACAGGTGCGGGACAACATGGAGTAGCAACAGCAACAGCGTGTGCTTATCTAGGATTAGATTGTGTAGTATACATGGGAGCAAAAGATGTGGAACGACAGAAACCAAATGTAGAGAAAATGCGCTTTTTAGGTGCTGAAGTCNNTTCTGTAACAAAAGGATCTTGTGGACTCCAAAGATGCAGTTAATCAAGCTCTAAAN

>BPD3

NNNNNNNNNNNNTAGGGCAGCAGTGGTNTCAACGCAGAGTACATGGGCTACTTGGCGATAGGCCTAATTAAGAAGCCTTTTATTTGATTAAGAGATGTTCTTATAGAAGTAAGAGCGTCTTTTTTGCGCAGGATTATTCTGTCGCCAGTTTTTTCTATGATTTTAACACTATAATTTTATGGAGAAAAGATGTTCAAACATAAACATCCTTTTGGGGGAGCGTTCCTTCCCGAAGAACTATTAGCCCCTATACAGAATCTAAAAGCGGAATGGGAGATTCTCAAAACTCAGCAAAGTTTTTTATCTGAACTAGATTGTATTTTGAAAAACTATGCGGGGAGACAAACTCCTCTGACTGAAGTTAAGAATTTTGCTCGAGCTATTGATGGCCCTAGAGTATTTCTTAAACGCGAAGATCTTTTGCATACAGGAGCACATAAACTGAATAATGCTCTTGGTCAGTGTTTGCTTGCTAAATATCTTGGGAAAACACGTGTTGTAGCTGAAACAGGTGCGGGACAACATGGAGTAGCAACAGCAACAGCGTGTGCTTATCTAGGATTAGATTGTGTAGTATACATGGGAGCAAAAGATGTGGAACGACAGAAACCAAATGTAGAGAAAATGCGCTTTTTAGGTGCTGAAGTCGTTTCTGTAACAAAAGGATCTTGTGGACTCAAAGATGCAGTTAATCAAGCTCTACAAGATTGGGCAACAACACACTCATTTACTCACTATTGCTTAGGATCGGCCTTAGGACCTTTACCTTATCCCGATATCGTTCGATTTTTTCAGTCTGTTATAAGCGCTGAAGTGAAAGAGCAAATCCATGCAGTTGCAGGAAGAGATCCTGATATTCTGATTGCATGTATCGGAGGTGGCTCCAACGCTATTGGATTTTTCCATCATTTTATCCCGAATCCAAAAGTCCAATTAATTGGAGTGGAAGGGGGGAGGACTGGGCATTTCTTCAGGAAAACATGCAGCACGTTTTGCAACAGGGCGACCTGGAGTATTCCACGAAGCTTGGCGTAATCATGGTCATAGCTGTTTCCNGNNNGAAATTGTTATCCG

>BPD4

NNNNNNNNNNNNNGGGCAGCAGTGGTNTCAACGCAGAGTACATGGGCTTGGCGATAGGCCTAATTAAGAAGCCTTTTATTTGATTAAGAGATGTTCTTATAGAAGTAAGAGCGTCTTTTTTGCGCAGGATTATTCTGTCGCCAGTTTTTTCTATGATTTTAACACTATAATTTTATGGAGAAAAGATGTTCAAACATAAACATCCTTTTGGGGGAGCGTTCCTTCCCGAAGAACTATTAGCCCCTATACAGAATCTAAAAGCGGAATGGGAGATTCTCAAAACTCAGCAAAGTTTTTTATCTGAACTAGATTGTATTTTGAAAAACTATGCGGGGAGACAAACTCCTCTGACTGAAGTTAAGAATTTTGCTCGAGCTATTGATGGCCCTAGAGTATTTCTTAAACGCGAAGATCTTTTGCATACAGGAGCACATAAACTGAATAATGCTCTTGGTCAGTGTTTGCTTGCTAAATATCTTGGGAAAACACGTGTTGTAGCTGAAACAGGTGCGGGACAACATGGAGTAGCAACAGCAACAGCGTGTGCTTATCTAGGATTAGATTGTGTAGTATACATGGGAGCAAAAGATGTGGAACGACAGAAACCAAATGTAGAGAAAATGCGCTTTTTAGGTGCTGAAGTCGTTTCTGTAACAAAAGGATCTTGTGGACTCAAAGATGCAGTTAATCAAGCTCTACAAGATTGGGCAACAACACACTCATTTACTCACTATTGCTTAGGATCGGCCTTAGGACCTTTACCTTATCCCGATATCGTTCGATTTTTTCAGTCTGTTATAAGCGCTGAAGTGAAAGAGCAAATCCATGCAGTTGCAGGAAGAGATCCTGATATTCTGATTGCATGTATCGGAGGTGGCTCCAACGCTATTGGATTTTTCCATCATTTTATCCCGAATCCAAAAGTCCAATTAATTGGAGTGGAAAGGGGAAGGACTGGGCATTTCTTCAGGAAAACATGCAGCACGTTTTGCAACAGGGCGACCTGGAGTATTCCACGAAGCTTGNNGTAATCATGGTCATAGCTGTT

>BPD5

NNNNNNNNNNNAGGGCAGCAGTGGTATCAACGCAGAGTACATGGGCTATGATTTTAACACTATAATTTTATGGAGAAAAGATGTTCAAACATAAACATCCTTTTGGGGGAGCGTTCCTTCCCGAAGAACTATTAGCCCCTATACAGAATCTAAAAGCGGAATGGGAGATTCTCAAAACTCAGCAAAGTTTTTTATCTGAACTAGATTGTATTTTGAAAAACTATGCGGGGAGACAAACTCCTCTGACTGAAGTTAAGAATTTTGCTCGAGCTATTGATGGCCCTAGAGTATTTCTTAAACGCGAAGATCTTTTGCATACAGGAGCACATAAACTGAATAATGCTCTTGGTCAGTGTTTGCTTGCTAAATATCTTGGGAAAACACGTGTTGTAGCTGAAACAGGTGCGGGACAACATGGAGTAGCAACAGCAACAGCGTGTGCTTATCTAGGATTAGATTGTGTAGTATACATGGGAGCAAAAGATGTGGAACGACAGAAACCAAATGTAGAGAAAATGCGCTTTTTAGGTGCTGAAGTCGTTTCTGTAACAAAAGGATCTTGTGGACTCAAAGATGCAGTTAATCAAGCTCTACAAGATTGGGCAACAACACACTCATTTACTCACTATTGCTTAGGATCGGCCTTAGGACCTTTACCTTATCCCGATATCGTTCGATTTTTTCAGTCTGTTATAAGCGCTGAAGTGAAAGAGCAAATCCATGCAGTTGCAGGAAGAGATCCTGATATTCTGATTGCATGTATCGGAGGTGGCTCCAACGCTATTGGATTTTTCCATCATTTTATCCCGAATCCAAAAGTCCAATTAATTGGAGTGGAAGGGGGAGGACTGGGCATTTCTTCNGGAAAANNTGCNNCACGTTTTGCAANNGGGCGAACTGGAATATTCCACGAAACTTGGCGTAATCATGGTCATNGCTGTTTCCTGTGNNAAATTGTTTTCCNNNACCAATTCCCAAA

>TRP1

NNNNNNNNNNNNNNNNGGGNNGCAGTGGTATCAACGCAGAGTACATGGGTGGCTTTCTTTTGAAAGCTGGCGCTTATCTACTTGGCGATAGGCCTAATTAAGAAGCCTTTTATTTGATTAAGAGATGTTCTTATAGAAGTAAGAGCGTCTTTTTTGCGCAGGATTATTCTGTCGCCAGTTTTTTCTATGATTTTAACACTATAATTTTATGGAGAAAAGATGTTCAAACATAAACATCCTTTTGGGGGAGCGTTCCTTCCCGAAGAACTATTAGCCCCTATACAGAATCTAAAAGCGGAATGGGAGATTCTCAAAACTCAGCAAAGTTTTTTATCTGAACTAGATTGTATTTTGAAAAACTATGCGGGGAGACAAACTCCTCTGACTGAAGTTAAGAATTTTGCTCGAGCTATTGATGGCCCTAGAGTATTTCTTAAACGCGAAGATCTTTTGCATACAGGAGCACATAAACTGAATAATGCTCTTGGTCAGTGTTTGCTTGCTAAATATCTTGGGAAAACACGTGTTGTAGCTGAAACAGGTGCGGGACAACATGGAGTAGCAACAGCAACAGCGTGTGCTTATCTAGGATTAGATTGTGTAGTATACATGGGAGCAAAAGATGTGGAACGACAGAAACCAAATGTAGAGAAAATGCGCTTTTTAGGTGCTGAAGTCGTTTCTGTAACAAAAGGATCTTGTGGACTCAAAGATGCAGTTAATCAAGCTCTACAAGATTGGGCAACAACACACTCATTTACTCACTATTGCTTAGGATCGGCCTTAGGACCTTTACCTTATCCCGATATCGTTCGATTTTTTCAGTCTGTTATAAGCGCTGAAGTGAAAGAGCAAATCCATGCAGTTGCAGGAAGAGATCCTGATATTCTGATTGCANGTATCGGAGGTGGCTCCAACGCTATTGGAATTTTTCCATCATTTTATCCCGAATCCAAAAGTCCAATTAATTGGAATGGGAAGGGGGGAGGANTGGGGCATTTNNTTCNGGAAAANNNNNCGGNNCGTTTTGNNACCAGGNNGACCTGGAATATTCCNNGAANGNTNG

>TRP2

NNNNNNNNNNNNNNNNNNNNNNGGGCAGCAGTGGTATCAACGCAGAGTACATGGGCAAAAAGGTGCGATATGAAAAATCAAGAGGAGTCTGGCTGGCAAGCTTTTCTGACATTATGCTCTAAAATGCAAAAAGAAAAGTTTTTACAAGACCTTTTTTCGCTGTTTTTGTCTTTTGGCGAACGTAAAGATGTCGCTTCTCGCTATCATATCATTCGAGCTCTTTTAGAAGGGGAGCTCACTCAAAGAGAGATAGCAGAGAAATACGGAGTCAGTATCGCACAAATTACCAGAGGATCTAATGCCCTTAAAGGATCAGATCCTCAATTTAAAGAGTTTTTACAAAAAGAGATCTGATCTTCTTTTGTAAAATACAAATAAGATTGAAAGTATTTGTATGCACGCGTTGTTAATGAACAAATATTCTGTTTTAGCAGTTTTGGTACGTAAGTATAGCTGCAGCATGCCATGCAAATCAGCTTTTCAAGCTGATTGCTTCCAAGATATTCAAAAATTCATCCTCTTACAGCGTGCCTGGCTTTCTTTTGAAAGCTGGCGCTTATCTACTTGGCGATAGGCCTAATTAAGAAGCCTTTTATTTGATTAAGAGATGTTCTTATAGAAGTAAGAGCGTCTTTTTTGCGCAGGATTATTCTGTCGCCAGTTTTTTCTATGATTTTAACACTATAATTTTATGGAGAAAAGATGTTCAAACATAAACATCCTTTTGGGGGAGCGTTCCTTCCCGAAGAACTATTAGCCCCTATACAGAATCTAAAAGCGGAATGGGAGATTCTCAAAACTCAGCAAAGTTTTTTATCTGAACTAGATTGTATTTTGAAAAACTATGCGGGGGAGACAAACTCCTCTGACTGAAGTTAAGAATTTTGCTCGAGCTATTGATGGCCCTAGAGTATTTCTTAAACGCGAAGATCTTTTGCATACAGGAGCACATAAACTGAATAATGCTCTTGGTCAGTGTTTGCTTGCTAAATATCTTGGGAAAACANGTGTTGTAGCTGAAACAGGTGCGGGACAACATGGAGTAGCAACAGCAACAGCGNGNGCTTAT

>TRP3

NNNNNNNNNNNNNNNNNNNGGGCAGCAGTGGTATCAACGCAGAGTACATGGGATTACAAAAAGGTGCGATATGAAAAATCAAGNGGAGTCTGGCTGGCAAGCTTTTCTGACATTATGCTCTAAAATGCAAAAAGAAAAGTTTTTACAAGACCTTTTTTCGCTGTTTTTGTCTTTTGGCGAACGTAAAGATGTCGCTTCTCGCTATCATATCATTCGAGCTCTTTTAGAAGGGGAGCTCACTCAAAGAGAGATAGCAGAGAAATACGGAGTCAGTATCGCACAAATTACCAGAGGATCTAATGCCCTTAAAGGATCAGATCCTCAATTTAAAGAGTTTTTACAAAAAGAGATCTGATCTTCTTTTGTAAAATACAAATAAGATTGAAAGTATTTGTATGCACGCGTTGTTAATGAACAAATATTCTGTTTTAGCAGTTTTGGTACGTAAGTATAGCTGCAGCATGCCATGCAAATCAGCTTTTCAAGCTGATTGCTTCCAAGATATTCAAAAATTCATCCTCTTACAGCGTGCCTGGCTTTCTTTTGAAAGCTGGCGCTTATCTACTTGGCGATAGGCCTAATTAAGAAGCCTTTTATTTGATTAAGAGATGTTCTTATAGAAGTAAGAGCGTCTTTTTTGCGCAGGATTATTCTGTCGCCAGTTTTTTCTATGATTTTAACACTATAATTTTATGGAGAAAAGATGTTCAAACATAAACATCCTTTTGGGGGAGCGTTCCTTCCCGAAGAACTATTAGCCCCTATACAGAATCTAAAAGCGGAATGGGAGATTCTCAAAACTCAGCAAAGTTTTTTATCTGAACTAGATTGTATTTTGAAAAACTATGCGGGGAGACAAACTCCTCTGACTGAAGTTAAGAATTTTGCTCGAGCTATTGATGGCCCTAGAGTATTTCTTAAACGCGAAGATCTTTTGCATACAGGAGCACATAAACTGAATAATGCTCTTGGTCAGTGTTTGCTTGCTAAATATCTTGGGAAAACACGTGTTGTAGCTGAAACAGNNGCGGGACAACATGGAGTAGCAACAGCAACAG

>TRP4

NNNNNNNNNNNNNNNNNTAGGGCAGCAGTGGTATCAACGCAGAGTACATGGGCCAAAAAGGTGCGATATGAAAAATCAAGAGGAGTCTGGCTGGCAAGCTTTTCTGACATTATGCTCTAAAATGCAAAAAGAAAAGTTTTTACAAGACCTTTTTTCGCTGTTTTTGTCTTTTGGCGAACGTAAAGATGTCGCTTCTCGCTATCATATCATTCGAGCTCTTTTAGAAGGGGAGCTCACTCAAAGAGAGATAGCAGAGAAATACGGAGTCAGTATCGCACAAATTACCAGAGGATCTAATGCCCTTAAAGGATCAGATCCTCAATTTAAAGAGTTTTTACAAAAAGAGATCTGATCTTCTTTTGTAAAATACAAATAAGATTGAAAGTATTTGTATGCACGCGTTGTTAATGAACAAATATTCTGTTTTAGCAGTTTTGGTACGTAAGTATAGCTGCAGCATGCCATGCAAATCAGCTTTTCAAGCTGATTGCTTCCAAGATATTCAAAAATTCATCCTCTTACAGCGTGCCTGGCTTTCTTTTGAAAGCTGGCGCTTATCTACTTGGCGATAGGCCTAATTAAGAAGCCTTTTATTTGATTAAGAGATGTTCTTATAGAAGTAAGAGCGTCTTTTTTGCGCAGGATTATTCTGTCGCCAGTTTTTTCTATGATTTTAACACTATAATTTTATGGAGAAAAGATGTTCAAACATAAACATCCTTTTGGGGGAGCGTTCCTTCCCGAAGAACTATTAGCCCCTATACAGAATCTAAAAGCGGAATGGGAGATTCTCAAAACTCAGCAAAGTTTTTTATCTGAACTAGATTGTATTTTGAAAAACTATGCGGGGAGACAAACTCCTCTGACTGAAGTTAAGAATTTTGCTCGAGCTATTGATGGCCCTAGAGTATTTCTTAAACGCGAAGATCTTTTGCATACAGGAGCACATAAACTGAATAATGCTCTTGGTCAGGGTTTGCTTGCTAAATATCTTGGGAAAACCCNTGTTGTAGCTGAAANNGGNGCGGGACAANNGGGANNANNAAANNAA

>TRP5

NNNNNNNNNNNNNNNNNNGGGCAGCAGTGGTATCAACGCAGAGTACATGGGTAAAGGATCAGATCCTCAATTTAAAGAGTTTTTACAAAAAGAGATCTGATCTTCTTTTGTAAAATACAAATAAGATTGAAAGTATTTGTATGCACGCGTTGTTAATGAACAAATATTCTGTTTTAGCAGTTTTGGTACGTAAGTATAGCTGCAGCATGCCATGCAAATCAGCTTTTCAAGCTGATTGCTTCCAAGATATTCAAAAATTCATCCTCTTACAGCGTGCCCGGCTTTCTTTTGAAAGCTGGCGCTTATCTACTTGGCGATAGGCCTAATTAAGAAGCCTTTTATTTGATTAAGAGATGTTCTTATAGAAGTAAGAGCGTCTTTTTTGCGCAGGATTATTCTGTCGCCAGTTTTTTCTATGATTTTAACACTATAATTTTATGGAGAAAAGATGTTCAAACATAAACATCCTTTTGGGGGAGCGTTCCTTCCCGAAGAACTATTAGCCCCTATACAGAATCTAAAAGCGGAATGGGAGATTCTCAAAACTCAGCAAAGTTTTTTATCTGAACTAGATTGTATTTTGAAAAACTATGCGGGGAGACAAACTCCTCTGACTGAAGTTAAGAATTTTGCTCGAGCTATTGATGGCCCTAGAGTATTTCTTAAACGCGAAGATCTTTTGCATACAGGAGCACATAAACTGAATAATGCTCTTGGTCAGTGTTTGCTTGCTAAATATCTTGGGAAAACACGTGTTGTAGCTGAAACAGGTGCGGGACAACATGGAGTAGCAACAGCAACAGCGTGTGCTTATCTAGGATTAGATTGTGTAGTATACATGGGAGCAAAAGATGTGGAACGACAGAAACCAAATGTAGAGAAAATGCGCTTTTTAGGTGCTGAAGTCGTTTCTGTAACAAAAGGATCTTGTGGACTCAAAGATGCAGTTAATCAAGCTCTACAAGATTGGGCAACAACACACTCATTTACTCACTATTGCTTAGGATCGGCCTTAGGACCTTTACCTTATCCCGATATCGTTCGATTTTTTCAG

>TRP7

NNNNNNNNNNNNNNNNNNNNNNAGCAGTGGTATCAACGCAGAGTACATGGGGTTTTTACAAAAAGAGATCTGATCTTCTTTTGTAAAATACAAATAAGATTGAAAGTATTTGTGTGCACGCGTTGTTAATGAACAAATATTCTGTTTTAGCAGTTTTGGTACGTAAGTATAGCTGCAGCATGCCATGCAAATCAGCTTTTCAAGCTGATTGCTTCCAAGATATTCAAAAATTCATCCTCTTACAGCGTGCCTGGCTTTCTTTTGAAAGCTGGCGCTTATCTACTTGGCGATAGGCCTAATTAAGAAGCCTTTTATTTGATTAAGAGATGTTCTTATAGAAGTAAGAGCGTCTTTTTTGCGCAGGATTATTCTGTCGCCAGTTTTTTCTATGATTTTAACACTATAATTTTATGGAGAAAAGATGTTCAAACATAAACATCCTTTTGGGGGAGCGTTCCTTCCCGAAGAACTATTAGCCCCTATACAGAATCTAAAAGCGGAATGGGAGATTCTCAAAACTCAGCAAAGTTTTTTATCTGAACTAGATTGTATTTTGAAAAACTATGCGGGGAGACAAACTCCTCTGACTGAAGTTAAGAATTTTGCTCGAGCTATTGATGGCCCTAGAGTATTTCTTAAACGCGAAGATCTTTTGCATACAGGAGCACATAAACTGAATAATGCTCTTGGTCAGTGTTTTGCTTGCTAAATATCTTGGGAAAAACACGTGTTTGTANCTGAAAACAGGTGCGGGGACANNNNNNAGTAGCAACAGCAACAGCGTGTGCNTTATCTAGGGATTAGATTGTGTAGTATACNTGGGAGCAAANNTNNTGNAACGACAAAAACCANNGNNNAGAAATNCNCNTTTTTAGGTGCTGAAGTCNTTTCCTGTACAAAAGGATCTTGTGGGANN

>TRP8

NNNNNNNNNNNNNNNNNAGGGCAGCAGTGGTATCACGCAGAGTACATGGGCTATGATTTTAACACTATAATTTTATGGAGAAAAGATGTTCAAACATAAACATCCTTTTGGGGGAGCGTTCCTTCCCGAAGAACTATTAGCCCCTATACAGAATCTAAAAGCGGAATGGGAGATTCTCAAAACTCAGCAAAGTTTTTTATCTGAACTAGATTGTATTTTGAAAAACTATGCGGGGAGACAAACTCCTCTGACTGAAGTTAAGAATTTTGCTCGAGCTATTGATGGCCCTAGAGTATTTCTTAAACGCGAAGATCTTTTGCATACAGGAGCACATAAACTGAATAATGCTCTTGGTCAGTGTTTGCTTGCTAAATATCTTGGGAAAACACGTGTTGTAGCTGAAACAGGTGCGGGACAACATGGAGTAGCAACAGCAACAGCGTGTGCTTATCTAGGATTAGATTGTGTAGTATACATGGGAGCAAAAGATGTGGAACGACAGAAACCAAATGTAGAGAAAATGCGCTTTTTAGGTGCTGAAGTCGTTTCTGTAACAAAAGGATCTTGTGGACTCAAAGATGCAGTTAATCAAGCTCTACAAGATTGGGCAACAACACACTCATTTACTCACTATTGCTTAGGATCGGCCTTAGGACCTTTACCTTATCCCGATATCGTTCGATTTTTTCAGTCTGTTATAAGCGCTGAAGTGAAAGAGCAAATCCATGCAGTTGCAGGAAGAGATCCTGATATTCTGATTGCATGTATCGGAGGTGGCTCCAACGCTATTGGATTTTTCCATCATTTTATCCCGAATCCAAAAGTCCAATTAATTGGAGTGGAAAGGGGGAGGACTGGGNATTTCTTCAGGAAAACATGCAGCACGTTTTGCAACGGGGCGACCTGGNAGTATTCCACNAAGCTTGGCGTAATCATGGTCATAGCTTGTTTCCTGNGNNAAATTGTTNTCCGCTCNCAATTCCNNNNANNNNACGAGCCGGAAGNNTAAANNGNTAANCNTNNNG

>TRP9

NNNNNNNNNNNNNNNGGGCAGCAGTGGTATCAACGCAGAGTACATGGGATTACAAAAAGGTGCGATATGAAAAATCAAGAGGAGTCTGGCTGGCAAGCTTTTCTGACATTATGCTCTAAAATGCAAAAAGAAAAGTTTTTACAAGACCTTTTTTCGCTGTTTTTGTCTTTTGGCGAACGTAAAGATGTCGCTTCTCGCTATCATATCATTCGAGCTCTTTTAGAAGGGGAGCTCACTCAAAGAGAGATAGCAGAGAAATACGGAGTCAGTATCGCACAAATTACCAGAGGATCTAATGCCCTTAAAGGATCAGATCCTCAATTTAAAGAGTTTTTACAAAAAGAGATCTGATCTTCTTTTGTAAAATACAAATAAGATTGAAAGTATTTGTATGCACGCGTTGTTAATGAACAAATATTCTGTTTTAGCAGTTTTGGTACGTAAGTATAGCTGCAGCATGCCATGCAAATCAGCTTTTCAAGCTGATTGCTTCCAAGATATTCAAAAATTCATCCTCTTACAGCGTGCCTGGCTTTCTTTTGAAAGCTGGCGCTTATCTACTTGGCGATAGGCCTAATTAAGAAGCCTTTTATTTGATTAAGAGATGTTCTTATAGAAGTAAGAGCGTCTTTTTTGCGCAGGATTATTCTGTCGCCAGTTTTTTCTATGATTTTAACACTATAATTTTATGGAGAAAAGATGTTCAAACATAAACATCCTTTTGGGGGAGCGTTCCTTCCCGAAGAACTATTAGCCCCTATACAGAATCTAAAAGCGGAATGGGAGATTCTCAAAACTCAGCAAAGTTTTTTATCTGAACTAGATTGTATTTTGAAAAACTATGCGGGGGAGACAAACTCCTCTGACTGAAGTTAAGAATTTTGCTCGAGCTATTGATGGCCCTAGAGTATTTCTTAAACGCGAAGATCTTTTGCATACAGGAGCACATAAACTGAATAATGCTCTTGGGTCAGGNTTTGCTTGNTAAATATCTTGGGAAAACANNGGNTGNTANNTGAAANNNG

>TRP10

NNNNNNNNNNNNNNNGGCAGCAGTGGTATCAACGCAGAGTACATGGGCAAAAAGGTGCGATATGAAAAATCAAGAGGAGTCTGGCTGGCAAGCTTTTCTGACATTATGCTCTAAAATGCAAAAAGAAAAGTTTTTACAAGACCTTTTTTCGCTGTTTTTGTCTTTTGGCGAACGTAAAGATGTCGCTTCTCGCTATCATATCATTCGAGCTCTTTTAGAAGGGGAGCTCACTCAAAGAGAGATAGCAGAGAAATACGGAGTCAGTATCGCACAAATTACCAGAGGATCTAATGCCCTTAAAGGATCAGATCCTCAATTTAAAGAGTTTTTACAAAAAGAGATCTGATCTTCTTTTGTAAAATACAAATAAGATTGAAAGTATTTGTATGCACGCGTTGTTAATGAACAAATATTCTGTTTTAGCAGTTTTGGTACGTAAGTATAGCTGCAGCATGCCATGCAAATCAGCTTTTCAAGCTGATTGCTTCCAAGATATTCAAAAATTCATCCTCTTACAGCGTGCCTGGCTTTCTTTTGAAAGCTGGCGCTTATCTACTTGGCGATAGGCCTAATTAAGAAGCCTTTTATTTGATTAAGAGATGTTCTTATAGAAGTAAGAGCGTCTTTTTTGCGCAGGATTATTCTGTCGCCAGTTTTTTCTATGATTTTAACACTATAATTTTATGGAGAAAAGATGTTCAAACATAAACATCCTTTTGGGGGAGCGTTCCTTCCCGAAGAACTATTAGCCCCTATACAGAATCTAAAAGCGGAATGGGAGATTCTCAAAACTCAGCAAAGTTTTTTATCTGAACTAGATTGTATTTTGAAAAACTATGCGGGGAGACAAACTCCTCTGACTGAAGTTAAGAATTTTGCTCGAGCTATTGATGGCCCTAGAGTATTTCTTAAACGCGAAGATCTTTTGCATACAGGAGCACATAAACTGAATAATGCTCTTGGTCAGTGTTTGCTTGCTAAATATCTTGGGAAAACACGTGTTGTAGCTGAAACAGGTGNNGGA

>TRP11

NNNNNNNNNNNNNNNNNNNNNNNNGGCAGCAGTGGTATCAACGCAGATTACATGGGGTTAGCAGTTTTGGTACGTAAGTATAGCTGCAGCATGCCATGCAAATCAGCTTTTCAAGCTGATTGCTTCCAAGATATTCAAAAATTCATCCTCTTACAGCGTGCCTGGCTTTCTTTTGAAAGCTGGCGCTTATCTACTTGGCGATAGGCCTAATTAAGAAGCCTTTTATTTGATTAAGAGATGTTCTTATAGAAGTAAGAGCGTCTTTTTTGCGCAGGATTATTCTGTCGCCAGTTTTTTCTATGATTTTAACACTATAATTTTATGGAGAAAAGATGTTCAAACATAAACATCCTTTTGGGGGAGCGTTCCTTCCCGAAGAACTATTAGCCCCTATACAGAATCTAAAAGCGGAATGGGAGATTCTCAAAACTCAGCAAAGTTTTTTATCTGAACTAGATTGTATTTTGAAAAACTATGCGGGGAGACAAACTCCTCTGACTGAAGTTAAGAATTTTGCTCGAGCTATTGATGGCCCTAGAGTATTTCTTAAACGCGAAGATCTTTTGCATACAGGAGCACATAAACTGAATAATGCTCTTGGTCAGTGTTTGCTTGCTAAATATCTTGGGAAAACACGTGTTGTAGCTGAAACAGGTGCGGGACAACATGGAGTAGCAACAGCAACAGCGTGTGCTTATCTAGGATTAGATTGTGTAGTATACATGGGAGCAAAAGATGTGGAACGACAGAAACCAAATGTAGAGAAAATGCGCTTTTTAGGTGCTGAAGTCGTTTCTGTAACAAAAGGATCTTGTGGACTCAAAGATGCAGTTAATCAAGCTCTACAAGATTGGGCAACAACACACTCATTTACTCACTATTGCTTAGGATCGGCCTTAGGACCTTTACCTTATCCCGATATCGTTCGATTTTTTCAGTCTGTTATAAGCGCTGAAGTGAAAGAGCAAATCCATGCAGTTGCAGGAAGAAATCCTGAAATTCTGATTGCATGTATCGGAAGTGGCNCCAAC

>TRP12

NNNNNNNNNNNNNNNNNNAGGGCAGCAGTGGTATCAACGCAGAGTACATGGGCAAAAAGGTGCGATATGAAAAATCAAGAGGAGTCTGGCTGGCAAGCTTTTCTGACATTATGCTCTAAAATGCAAAAAGAAAAGTTTTTACAAGACCTTTTTTCGCTGTTTTTGTCTTTTGGCGAACGTAAAGATGTCGCTTCTCGCTATCATATCATTCGAGCTCTTTTAGAAGGGGAGCTCACTCAAAGAGAGATAGCAGAGAAATACGGAGTCAGTATCGCACAAATTACCAGAGGATCTAATGCCCTTAAAGGATCAGATCCTCAATTTAAAGAGTTTTTACAAAAAGAGATCTGATCTTCTTTTGTAAAATACAAATAAGATTGAAAGTATTTGTATGCACGCGTTGTTAATGAACAAATATTCTGTTTTAGCAGTTTTGGTACGTAAGTATAGCTGCAGCATGCCATGCAAATCAGCTTTTCAAGCTGATTGCTTCCAAGATATTCAAAAATTCATCCTCTTACAGCGTGCCTGGCTTTCTTTTGAAAGCTGGCGCTTATCTACTTGGCGATAGGCCTAATTAAGAAGCCTTTTATTTGATTAAGAGATGTTCTTATAGAAGTAAGAGCGTCTTTTTTGCGCAGGATTATTCTGTCGCCAGTTTTTTCTATGATTTTAACACTATAATTTTATGGAGAAAAGATGTTCAAACATAAACATCCTTTTGGGGGAGCGTTCCTTCCCGAAGAACTATTAGCCCCTATACAGAATCTAAAAGCGGAATGGGAGATTCTCAAAACTCAGCAAAGTTTTTTATCTGAACTAGATTGTATTTTGAAAAACTATGCGGGGAGACAAACTCCTCTGACTGAAGTTAAGAATTTTGCTCGAGCTATTGATGGCCCTAGAGTATTTCTTAAACGNNAAGATCTTTTGCATACAGGANNACATAAACTGAATAATGCNNTNGGTCAGTGTTTGCTTGCTAAANATCTNGGGGAAANNNNNNGTTGTANTTGAAANA

>TRP13

NNNNNNNNNNNNNNTNNNNNNGGGCAGCAGTGGTATCAACGCAGAGTACATGGGAGGATCAGATCCTCAATTTAAAGAGTTTTTACAAAAAGAGATCTGATCTTCTTTTGTAAAATACAAATAAGATTGAAAGTATTTGTATGCACGCGTTGTTAATGAACAAATATTCTGTTTTAGCAGTTTTGGTACGTAAGTATAGCTGCAGCATGCCATGCAAATCAGCTTTTCAAGCTGATTGCTTCCAAGATATTCAAAAATTCATCCTCTTACAGCGTGCCTGGCTTTCTTTTGAAAGCTGGCGCTTATCTACTTGGCGATAGGCCTAATTAAGAAGCCTTTTATTTGATTAAGAGATGTTCTTATAGAAGTAAGAGCGTCTTTTTTGCGCAGGATTATTCTGTCGCCAGTTTTTTCTATGATTTTAACACTATAATTTTATGGAGAAAAGATGTTCAAACATAAACATCCTTTTGGGGGAGCGTTCCTTCCCGAAGAACTATTAGCCCCTATACAGAATCTAAAAGCGGAATGGGAGATTCTCAAAACTCAGCAAAGTTTTTTATCTGAACTAGATTGTATTTTGAAAAACTATGCGGGGAGACAAACTCCTCTGACTGAAGTTAAGAATTTTGCTCGAGCTATTGATGGCCCTAGAGTATTTCTTAAACGCGAAGATCTTTTGCATACAGGAGCACATAAACTGAATAATGCTCTTGGTCAGTGTTTGCTTGCTAAATATCTTGGGAAAACACGTGTTGTAGCTGAAACAGGTGCGGGACAACATGGAGTAGCAACAGCAACAGCGTGTGCTTATCTAGGATTAGATTGTGTAGTATACATGGGAGCAAAAGATGTGGAACGACAGAAACCAAATGTAGAGAAAATGCGCTTTTTAGGTGCTGAAGTCGTTTCTGTAACAAAAGGATCTTGTGGACTCAAAGATGCAGTTAATCAAGCTCTACAAGATTGGGCAACAACACACTCATTTACTCACTATTGCTTAGGATCGGCCTTAGGA
