# Supplementary material for: A bipartite iron-dependent transcriptional regulation of the tryptophan salvage pathway in Chlamydia trachomatis
Source: eLife. 2019 Apr 2;8:e42295. doi: 10.7554/eLife.42295 (PMC6504234; doi:10.7554/eLife.42295)
Supplement: Supplementary file 4. [file elife-42295-supp4.docx]

February 16 2018

>250-1

NNNNNNNNNNNNNNNANGTAGATGTCGCTTCTCTCTATCATATCATTCGAGCTCTTTTAGAAGGGGAGCTCACTCAAAGAGAGATAGCAGAGAAATACGGAGTCAGTATCGCACAAATTACCAGAGGATCTAATGCCCTTAAAGGATCAGATCCTCAATGAAAAAAAAAAAAAAAAAAAAAAAAAAAAAAGTACTCTGCGTTGATACCACTGCTTGCCCTATAGTGAGTCGTATTAGGAATTCACTGGCCGTCGTTTTACAACGTCGTGACTGGGAAAACCCTGGCGTTACCCAACTTAATCGCCTTGCAGCACATCCCCCTTTCGCCAGCTGGCGTAATAGCGAAAAGGCCCGCACCGATCGTCCTTCCCAACAGTTGCGCAGCATGGGNGGNGAATGGNACCTGTTGCNGTATTCNTGNTNNNNAGCNNNTG

511662

>250-2

NNNNNNNNNNNNNANGTAAGNTGTCGCTTCTCGCTATCATATCATTCGAGCTCTTTTAGAAGGGGAGCTCACTCAAAGAGAGATAGCAGAGAAATACGGAGTCAGTATCGCACAAATTACCAGAGGATCTAATGCCCTTAAAGGATCAGATCCTCAATTAAAAAAAAAAAAAAAAAAAAAAAAAAAAGTACTCTGCGTTGATACCACTGCTTGCCCTATAGTGAGTCGTATTAGGAATTCACTGGCCGTCGTTTTACAACGTCGTGACTGGGAAAACCCTGGCGTTACCCAACTTAATCGCCTTGCAGCACATCCCCCTTTCGCCAGCTGGCGTAATAGCGAAGAGGCCCGCACCGATCGCCCTTCCCAACAGTTGCGCAGCCTGAATGGCGAATGGCGCCTGATGCGGTATTTTCTCCTTACGCATCTGTGCGGTATTTCACACCGCATATGGTGCACTCTCAGTACAATCTGCTCTGATGCCGCATAGTTAAGCCAGCCCCGACACCCGCCAACACCCGCTGACGCGCCCTGACGGGCTTGTCTGCTCCCGGCATCCGCTTACAGACAAGCTGTGACCGTCTCCGGGAGCTGCATGTGTCAGAGGTTTTCACCGTCATCACCGAAACGCGCGAGACGAAAGGGCCNNGNGANACCGCCTATTTTTATAGGTTAATGNCNGGATANNAATGGGTTTCNTANACGTCNGNT

511663

>250-4

NNNNNNNNNNNNNANGTANGATGTCGCTTCTCGCTATCATATCATTCGAGCTCTTTTAGAAGGGGAGCTCACTCAAAGAGAGATAGCAGAGAAATACGGAGTCAGTATCGCACAAATTACCAGAGGATCTAATGCCCTTAAAGGATCAGATCCTCAAAAAAAAAAGAAAAAAAAAAAAAAAAAAAAAAAAAAAAAAAAGTACTCTGCGTTGATACCACTGCTTGCCCTATAGTGAGTCGTATTAGGAATTCACTGGCCGTCGTTTTACAACGTCGTGACTGGGAAAACCCTGGCGTTACCCAACTTAATCGCCTTGCAGCACATCCCCCTTTCGCCAGCTGGCGTAATAGCGAAAAGGCCCGCACCGATCGCCCTTCCCAACAGTTGCGCAGCCTGAATGGCGAATGGCGCCTGATGCGGTATTTTCTCCTTACGCATCTGTGCGGTATTTCCCACCGCATATGGTGCACTCTCAGTACAATCTGCTCTGATGCCGCATAGTTAAGCCAGCCCCGACAACCGCCAACACCCGCTGACGGGCCCGGANGGGNGNGNCTGGCTTCCGCTTACGGGATANNGTGTNACCGNCNCC

511661

>250-5

NNNNNNNNNNNNNNGTAAGNTGTCGCTTCTCGCTATCATATCATTCGAGCTCTTTTAGAAGGGGAGCTCACTCAAAGAGAGATAGCAGAGAAATACGGAGTCAGTATCGCACAAATTACCAGAGGATCTAATGCCCTTAAAGGATCAGATCCTCAATTTAAAAAAAAAAAAAAAAAAAAAAAAAAAAAAAGTACTCTGCGTTGATACCACTGCTTGCCCTATAGTGAGTCGTATTAGGAATTCACTGGCCGTCGTTTTACAACGTCGTGACTGGGAAAACCCTGGCGTTACCCAACTTAATCGCCTTGCAGCACATCCCCCTTTCGCCAGCTGGCGTAATAGCGAAGAGGCCCGCACCGATCGCCCTTCCCAACAGTTGCGCAGCCTGAATGGCGAATGGCGCCTGATGCGGTATTTTCTCCTTACGCATCTGTGCGGTATTTCACACCGCATATGGTGCACTCTCAGTACAATCTGCTCTGATGCCGCATAGTTAAGCCAGCCCCGACACCCGCCAACACCCGCTGACGCGCCCTGACGGGCTTGTCTGCTCCCGGCATCCGCTTACAGACAAGCTGTGACCGTCTCCGGGAGCTGCATGTGTCAGAGGTTTTCACCGTCATCACCGAAACGCGCGAGACGAAAGGGCCTCGTGATACGCCTATTTTTATAGGTTAATGTCATGATAATAATGGTTTCTTAGACGTCAGGTGGCACTTTTCGGGGAAATGTGCGCGGAACCCCTATTTGTTTATTTTTCTAAATACATTCAAATATGTATCCGCTCATGAGACAATAACCCTGATAAATGCTTCAATAATATTGAAAAAGGAAGAGTATGAGTATTCAACATTTCCGTGTCGCCCTTATTCCCTTTTTTGCGGCATTTTGCCTTCCTGTTTTTGCTCACCCAGAAACGCTGGTGAAAGTAAAAGATGCTGAAGATCAGTTGGGTGCACGANNGGGTTACATCGAACTGGATCTCAACAGCGGTAAGATCCTTGAAAGTTTTCGCCCCGAANAANNGTTTTCCAATGATGANCNNTTTTAAAGTTNNGCTAANNGGNNNGNNNTNNNCCCGTNTTGACGCCGGGCAAAANCAANNCGGTCNCCNCAANNCCTATTNTCAAANNNACTTGGTTNNNNNCTCCNNNNTCCNNGAAANNNNNNTTACGNNNGGNNNNNNNNTAAAAAAANNNNNCNNNNNNNCNNAANCCNGNNNNNNAANCC

511667

>250-6

GNNNNNNNNNACGNAGNTGTCGCTTCTCGCTATCATATCATTCGAGCTCTTTTAGAAGGGGAGCTCACTCAAAGAGAGATAGCAGAGAAATACGGAGTCAGTATCGCACAAATTACCAGAGGATCTAATGCCCTTAAAGGATCAGATCCTCAATTTGAAAAAAAAAAAAAAAAAAAAAAAAAAAAAAAAAAAGTACTCTGCGTTGATACCACTGCTTGCCCTATAGTGAGTCGTATTAGGAATTCACTGGCCGTCGTTTTACAACGTCGTGACTGGGAAAACCCTGGCGTTACCCAACTTAATCGCCTTGCAGCACATCCCCCTTTCGCCAGCTGGCGTAATAGCGAAGAGGCCCGCACCGATCGCCCTTCCCAACAGTTGCGCAGCCTGAATGGCGAATGGCGCCTGATGCGGTATTTTCTCCTTACGCATCTGTGCGGTATTTCACACCGCATATGGTGCACTCTCAGTACAATCTGCTCTGATGCCGCATAGTTAAGCCAGCCCCGACACCCGCCAACACCCGCTGACGCGCCCTGACGGGCTTGTCTGCTCCCGGCATCCGCTTACAGACAAGCTGTGACCGTCTCCGGGAGCTGCATGTGTCAGAGGTTTTCACCGTCATCACCGAAACGCGCGAGACGAAAGGGCCTCGTGATACGCCTATTTTTATAGGTTAATGTCATGATAATAATGGTTTCTTAGACGTCAGGTGGCACTTTTCGGGGAAATGTGCGCGGAACCCCTATTTGTTTATTTTTCTAAATACATTCAAATATGTATCCGCTCATGAGACAATAACCCTGATAAATGCTTCAATAATATTGAAAAAGGAAGAGTATGAGTATTCAACATTTCCGTGTCGCCCTTATTCCCTTTTTTGCGGCATTTTGCCTTCCTGTTTTTGCTCACCCAGAAACGCTGGTGAAAGTAAAAGATGCTGAAGATCAGTTGGGTGCACGAGTGGGTTACATCGAACTGGATCTCAACAGCGGTAAGATCCTTGAAANTTTTCCCCCNGAAAAACGTTTTCCAATGATGAGCACTTTTAAAGTTCTGCTATNTGGNNNGGNNTTATCCCGTATTGACGCCGGGCAANANCAACTCGGTNNNNGCATACCNTATTCNNAAANTGACTTGGTTGAGTANNNNCCNNTNNNNAAAAACCTCTTACNAAGGC

511664

>250-7

NNNNNNNNNNGACGTAAGATGTCGCTTCTCGCTATCATATCATTCGAGCTCTTTTAGAAGGGGAGCTCACTCAAAGAGAGATAGCAGAGAAATACGGAGTCAGTATCGCACAAATTACCAGAGGATCTAATGCCCTTAAAGGATCAGATCCTCAATTAAAAAAAAACAAAAAAAAAAAAAAAAAAAAAAAAAAAAAAGTACTCTGCGTTGATACCACTGCTTGCCCTATAGTGAGTCGTATTAGGAATTCACTGGCCGTCGTTTTACAACGTCGTGACTGGGAAAACCCTGGCGTTACCCAACTTAATCGCCTTGCAGCACATCCCCCTTTCGCCAGCTGGCGTAATAGCGAAGAGGCCCGCACCGATCGCCCTTCCCAACAGTTGCGCAGCCTGAATGGCGAATGGCGCCTGATGCGGTATTTTCTCCTTACGCATCTGTGCGGTATTTCACACCGCATATGGTGCACTCTCAGTACAATCTGCTCTGATGCCGCATAGTTAAGCCAGCCCCGACACCCGCCAACACCCGCTGACGCGCCCTGACGGGCTTGTCTGCTCCCGGCATCCGCTTACAGACAAGCTGTGACCGTCTCCGGGAGCTGCATGTGTCANAGGTTTTNACCGTCATCNCCGAAACGTNNNAAGACGAAAGGGNATNNNNGATACGCCTATTTNNNNNGGNNTAANNNNNNG

511663

>400-1

NNNNNNNNNNNNANGTAAGATGTCGCTTCTCGCTATCATATCATTCGAGCTCTTTTAGAAGGGGAGCTCACTCAAAGAGAGATAGCAGAGAAATACGGAGTCAGTATCGCACAAATTACCAGAGGATCTAATGCCCTTAAAGGATCAGATCCTCAATTTAAAGAGTTTTTACAAAAAGAGATCTGATCTTCTTTTGTAAAATACAAATAAGATTGAAAGTATTTGTATGCACGCGTTGTTAATGAACAAATATTCTGTTTTAGCAGTTTTGGTACGTAAGTATANCTGCAGCATGCCATGCAAATCAGCTTTTCAAGCGGATTGCTTCCTNGGAAAAGAGAAGCGAAAAAAAAANAAGGGACNCGGNGGGNGTACCNNGGNTTGCNGNAGAGTGAGGGNGANGNGGTNATCANTGGTNTGCTCTGTATTGGNGTGTGANGGNAANAACANGNTGCNTGNGNGCACGATCTNAN

>400-2

NNNNNNNNNNNNNNNANGNNNNATGTCGCTTCTCGCTATCATATCATTCGAGCTCTTTTAGAAGGGGAGCTCACTCAAAGAGAGATAGCAGAGAAATACGGAGTCAGTATCGCACAAATTACCAGAGGATCTAATGCCCTTAAAGGATCAGATCCTCAATTTAAAGAGTTTTTACAAAAAGAGATCTGATCTTCTTTTGTAAAATACAAATAAATTGAAAGTATTTGTATGCACGCGTTGTTAATGANNANTATTCTGTTTTAGCAGTTTTGGTACGTAAGTATAGCTGCAGCATGCCATAGAAAAAAAAAAAAAAAAAANGAAAAAAAAAANGTACTCTGCGTNNATACCACTGCTTGCCNNATAGTGAGTCGTATTAGGAATTNACTGGNNGTCGTTNNACAACGCGTGACTGGGAAAACCCTGCNNTNACNNAACTTANTNNCCTTGNTCNCATCCCCCTTTCGCCAGCTGGCGTAATAACGAAGAGGCCCGCACCGATCGCCCTTCCCAACANNTGNCACTCTGANGGNGANTGGNCNTGATGCGGANGTNCCNTAACATNNNGGGTANTACACCGGNNANNNGNNTNNNAAA

>400-4

NNNNNNNNNNGANGTAAGATGTCGCTTCTCGCTATCATATCATTCGAGCTCTTTTAGAAGGGGAGCTCACTCAAAGAGAGATAGCAGAGAAATACGGAGTCAGTATCGCACAAATTACCAGAGGATCTAATGCCCTTAAAGGATCAGATCCTCAATTTAAAGAGTTTTTACAAAAAGAGATCTGATCTTCTTTTGTAAAATACAAATAAGATTGAAAGTATTTGTATGCACGCGTTGTTAATGAACAAATATTCTGTTTTAGCAGTTTTGGTACGTAAGTATAGCTGCAGCATGCCATGCAAATCAGCTTTTCAAGCTGATTGCTTCCAAGATCAAAAAAAAAAAAAAAAAAAAAAAAAGAAGTACTCTGCGTTGATACCACTGCTTGCCCTATAGGGAGTCGTATTAGGAATTCACNGGCCGTGNTTTTACGACGTCATGGACTGGGAAAACCCTNNNGTTACCCACTTAATCGCCTTGCAGCACATCCCCCTTTCGCCNGCTGGGGTAATAGCGAAAAAGCGCCCNCGNATCGCCCTGCCCACCTCTTTGCGNNNGCAATGGTNAANGGNGAGCGCGAATGAANTGANNGCGAGNAGNGACATGCTNTNNGTNNGCCAAGNNNGGNGTAATCTCTGGTCANAGNNGTTTCNNNNNNN

>400-5

NNNNNNNNNNANGTAAGATGTCGCTTCTCGCTATCATATCATTCGAGCTCTTTTAGAAGGGGAGCTCACTCAAAGAGAGATAGCAGAGAAATACGGAGTCAGTATCGCACAAATTACCAGAGGATCTAATGCCCTTAAAGGATCAGATCCTCAATTTAAAGAGTTTTTACAAAAAGAGATCTGATCTTCTTTTGTAAAATACAAATAAGATTGAAAGTATTTGTATGCACGCGTTGTTAATGAACAATATTCTGTTTTAGCATTTTGGTACGTAAGTATAGCTGNNCATGCCTGCAAATCAGCTTTTCAGCTGATTGCCAAAAAANAAAANAAAAGAAAAAAAAANGTACTNGGNNTGNNNCCNCTGCTNNCNNATAGTNNNTCGTNNNAGGANNCACTGGCCGNNTTNNTACANNTNGGACTGGGNNAACCCTGGCGTTACCCNACTTAATNNCCTTGCAGCACATCCCCCTTTCNCNCTGGCGNNATNNNGAAANGCNGANNNANNGCCCNTCNAANNGTGNNCNCCTGANGTNNNTGNNNTGANGNGNGNTNTNNGCTNGANNCNNNNNGNNAANNTNNNGNNGGANNCCTNCGNCNCA

>400-6

NNNNNNNNNNNNCNANGNNGATGTCGCTTCTCGCTATCATATCATTCGAGCTCTTTTAGAAGGGGAGCTCACTCAAAGANAGATAGCAGAGAAATACGGAGTCAGTATCGCACAAATTACCAGAGGATCTAATGCCCTTAAAGGATCAGATCCTCAATTTAAAGAGTTTTTACAAAAAGAGATCTGATCTTCTTTTGTAAAATACAAATAAGATTGAAAGTATTTGTATGCACGCGTTGTTAATGAACAAATATTCTGTTTTAGCAGTTTTGGTACGTAAGTATAGCTGCAGCATGCCATGCAAAAAAAAAAAAAAAAAAAAAAAAAAAAAAGTACTCTGCGTTGATACCACTGCTTGCCCTATAGTGAGTCGTATTAGGAATTCACTGGCCGTCGTTTTACAACGTCGTGACTGGGAAAACCCTGGCGTTACCCAACTTAATCGCCTTGCAGCACATCCCCCTTTCGCCAGCTGGCGTAATAGCGAAGAGGCCCGCACCGATCGCCCTTCCCAACAGTTGCGCAGCCTGAATGGCGAATGGCGCCTGATGCGGTATTTTCTCCTTACGCATCTGTGCGGTATTTCACACCGCATATGGTGCACTCTCAGTACAATCTGCTCTGATGCCGNATANTTAAACCAGCCCCGACNCCCGCCAACNCCCGCTGANGCGNCCTGACGGGCTTGGTCTGCTCTNTGNATCNGNTTNANNACNAN

511809

>400-10

GNNNNNNNNNNANGNNGATGTCGCTTCTCGCTATCATATCATTCGAGCTCTTTTAGAAGGGGAGCTCACTCAAAGAGAGATAGCAGAGAAATACGGAGTCAGTATCGCACAAATTACCAGAGGATCTAATGCCCTTAAAGGATCAGATCCTCAATTTAAAGAGTTTTTACAAAAAGAGATCTGATCTTCTTTTGTAAAATACAAATAAGATTGAAAGTATTTGTATGCACGCGTTGTTAATGAACAAATATTCTGTTTTAGCAGTTTTGGTACGTAAGTATAGCTAAAAAAAAAAAAAAAAAAAAAAAAAAAAAGTACTCTGCGTTGATACCACTGCTTGCCCTATAGTGAGTCGTATTAGGAATTCACTGGCCGTCGTTTTACAACGTCGTGACTGGGAAAACCCTGGCGTTACCCAACTTAATCGCCTTGCAGCACATCCCCCTTTCGCCAGCTGGCGTAATAGCGAAGAGGCCCGCACCGATCGCCCTTCCCAACAGTTGCGCAGCCTGAATGGCGAATGGCGCCTGATGCGGTATTTTCTCCTTACGCATCTGTGCGGTATTTCACACCGCATATGGTGCACTCTCAGTACAATCTGCTCTGATGCCGCATAGTTAAGCCAGCCCCGACACCCGCCAACACCCGCTGACGCGCCCTGACGGGCTTGTCTGCTCCCGGCATCCGCTTACAGACAAGCTGTGACCGTCTCCGGGAGCTGCATGTGTCAGAGGTTTTCACCGTCATCACCGAAACGCGCGAGACGAAAGGGCCTCGTGATACGCCTATTTTTATAGGTTAATGTCATGATAATAATGGGTTTCTTAGACGTCAGGTGGCACTTTTTCGGGGAAATGTGCGCGGNAACCCCANTTTGGTNNTTNNTCCNAANNNNNTCAAANANGTATCNGNNNCATGAGAACANAACCCCGGNNAAANNCCTCCAAANNNATGGGAAAAAGGAAGAGGAAGGAGGATTCNANANTTTTCCTGGNNNNCCNNTTATNNCCTTTTTTNGGCGGANTTTTGGCCNNNNNNGGTTTTTGGNNCCNCNNNNAANNNCNGGGGNNAANNNNAAAAANNNNG

511792

>450-1

GNNNNNNNNNNNNANGNNGATGTCGCTTCTCGCTATCATATCATTCGAGCTCTTTTAGAAGGGGAGCTCACTCAAAGANAGATAGCAGAGAAATACGGAGTCAGTATCGCACAAATTACCAGAGGATCTAATGCCCTTAAAGGATCAGATCCTCAATTTAAAGAGTTTTTACAAAAAGAGATCTGATCTTCTTTTGTAAAATACAAATAAGATTGAAAGTATTTGTATGCACGCGTTGTTAATGAACAAATATTCTGTTTTAGCAGTTTTGGTACGTAAGTATAGCTGCAGCATGCCATGCAAATCAGCTTTTCAAGCTGATTGCTTCCAAGATATTCAAAAATTCATCCTCTTACAGCGCGCCTGGCTTCGNTTTGAAAGCNGAATTTACNNNAGGAGAAATGAACAAAGTGCNNGGGGTTCCNNCTTNNGAGGTGCTCTTTTTTGAGNNGAATTTCGTATTCTCGGNNNGNNN

>450-2
NNNNNNNNNNNNNNGNNNGATGTCGCTTCTCGCTATCATATCATTCGAGCTCTTTTAGAAGGGGAGCTCACTCAAAGNNAGATAGCAGAGAAATACGGAGTCAGTATCGCACAAATTACCAGAGGATCTAATGCCCTTAAAGGATCAGATCCTCAATTTAAAGAGTTTTTACAAAAAGAGATCTGATCTTCTTTTGTAAAATACAAATAAGATTGAAAGTATTTGTATGCACGCGTTGTTAATGAACAAATATTCTGTTNTANCAGTTTTGGTTCGGTTGNGGAGCTGGTGCATGTCGTGGAAGGCANGTTTATCGCGNGAATTTGGTGNGAGGTTGTANGGAGATTTAAAATTCATCGCCTCGCTTCGNGCNTGGATGGCTTTTTNTNAGTGGGGGGGAANAACAAAAGGAAAGGGGCGNNANANNNNGNANNT

>450-3

NNNNNNNNNNGACGTAAGATGTCGCTTCTCGCTATCATATCATTCGAGCTCTTTTAGAAGGGGAGCTCACTCAAAGAGAGATAGCAGAGAAATACGGAGTCAGTATCGCACAAATTACCAGAGGATCTAATGCCCTTAAAGGATCAGATCCTCAATTTAAAGAGTTTTTACAAAAAGAGATCTGATCTTCTTTTGTAAAATACAAATAAGATTGAAAGTATTTGTATGCACGCGTTGTTAATGAACAAATATTCTGTTTTAGCAGTTTTGGTACGTAAGTATAGCTGCAGCAAAAAAAAAAAAAAAAAAAAAAAAAAAAAAAAAAAAAAAAAAAAAAAAAAAAAAAAAAAAAAAAAAAAAAAAAAAAAAAAAAAAAAAAAAAAAAAAAAAANATTAAAAAAAAAAAAAAAAAAAAAAAAAGTTCCCCGGGGTTAACCCCCGGGTTGCCCCAAAGGGGGGCCAAAAAAGAAATTCCGGGGCCCTTTTTTTAAACCGGGGGGGGGGGAAAACCCGGGGGTTCCCCCAATTTNNNCCCTTGGGANANNCCCCCCTTTTNNCCGGGGGGGNAAAAAAAAAAANGGCCCCCCCNNNNCCCCNNTCCNAAANNTTNNCCCCCCANAAGGGNAAGGGGCCCCGGGNGNGTTTTTTTNNCNTANCNCTTGGGGGGGGTTTTNCCCCCCNNAGGGGGGGCCCNNNNNNAANNNNNGGNTNNGGGNCNNNANNNTNAAANNNNCCCCAANCCCCNNAAANNCNNNNNAANNCCCCNNGGNGGGTTNNNNTTCCCCNGGNNNCCCTTNAAAAAAANNNNNGNNCNCTCCNNGGGGANNGNNNGNGNNAAAGNNTTTTCCCNNNNTCNCCCNNAAANNNNNGNNANAAAAGGGGGCCCCNNGNACCCCNTNTNTTTNNNNGNGNNNNNNNNNNGNNNNNNNGGGGNTNNNNNNANCGCNNGGNGGNNCNNNNGNGNNNNNNTGGTGGNGNGNANNNNNNCNNNTNNNNNNNTNNNNNNNANNNNNTNNNNANANNNNNNCGNNNCGATNAGNANNNNNNNNNNNTCNNANNNA

>450-5

NNNNNNNNNCNNNGTAAGATGTCGCTTCTCGCTATCATATCATTCGAGCTCTTTTAGAAGGGGAGCTCACTCAAAGAGAGATAGCAGAGAAATACGGAGTCAGTATCGCACAAATTACCAGAGGATCTAATGCCCTTAAAGGATCAGATCCTCAATTTAAAGAGTTTTTACAAAAAGAGATCTGATCTTCTTTTGTAAAATACAAATAAGATTGAAAGTATTTGTATGCACGCGTTGTTAATGAACAAATATTCTGTTTTAGCAGTTTTGGTACGTAAGTATAGCTGCAGCATGCCATGCAAATCAGCTTTTCAAGCTGATTGCTTCCAAGATATTCAAAAATTCATCCTCTTACAGCGTGCCTGGCTTTCTTTTGAAAGCAGAAAAAAAAAAAAAAAAAAAAAAAAAAGTACTCTGCGTTGATACCACTGCTTGCCCTATAGTGAGTCGTATTAGGAATTCACTGGCCGTCGTTTTACAACGTCGTGACTGGGAAAACCCTGGCGTTACCCAACTTAATCGCCTTGCAGCACATCCCCCTTTCGCCAGCTGGCGTAATAGCGAAGAGGCCCGCACCGATCGCCCTTCCCAACAGTTGCGCAGCCTGAATGGCGAATGGCGCCTGATGCGGTATTTTCTCCTTACGCATCTGTGCGGTATTTCACACCGCATATGGTGCACTCTCAGTACAATCTGCTCTGATGCCGCATAGTTAAGCCAGCCCCGACACCCGCCAACACCCGCTGACGCGCCCTGACGGGCTTGTCTGCTCCCGGCATCCGCTTACAGACAANCTGTGGACCGTCTCNGNNNNC

>450-6

NNNNNNNNNNNNANGNAGNTGTCGCTTCTCGCTATCATATCATTCGAGCTCTTTTAGAAGGGGAGCTCACTCAAAGAGAGATAGCAGAGAAATACGGAGTCAGTATCGCACAAATTACCAGAGGATCTAATGCCCTTAAAGGATCAGATCCTCAATTTAAAGAGTTTTTACAAAAAGAGATCTGATCTTCTTTTGTAAAATACAAATAAGATTGAAAGTATTTGTATGCACGCGTTGTTAATGAACAAATATTCTGTTTTAGCAGTTTTGGTACGTAAGTATAGCTGCAGCATGCCATGCAAATCAGCTTTTCAAGCTGATTGCTTCCAAGATATTCAAAAATTCATCCTCTTACAGCGTGCCTGGCTTTCTTTTGAAAGCTGGCGCTTAAAAAAAAAAAAAAAAAAAAAAAAAAAAAGTACTCTGCGTTGATACCACTGCTTGCCCTATAGTGAGTCGTATTAGGAATTCACTGGCCGTCGTTTTACAACGTCGTGACTGGGAAAACCCTGGCGTTACCCAACTTAATCGCCTTGCAGCACATCCCCCTTTCGCCNNNTGGCGTAATAGCGANNNGGCCCGCGCCGATCGCCCGTNCNANNAGTTGCGCACNCTGNNTGNNCAATGGCGNCCTGATGCGGNNNNNTCGTCTTTNNGANATCTGTGCCGGCATTTCACACNGNNNNNGGGGGCACTTCTCANNNACAGNNCT

>450-11

NNNNNNNNNNNNNNNGTAAGATGTCGCTTCTCGCTATCATATCATTCGAGCTCTTTTAGAAGGGGAGCTCACTCAAAGAGAGATAGCAGAGAAATACGGAGTCAGTATCGCACAAATTACCAGAGGATCTAATGCCCTTAAAGGATCAGATCCTCAATTTAAAGAGTTTTTACAAAAAGAGATCTGATCTTCTTTTGTAAATACAAATAAGATTGAAAGATTTGTATGCACGCGTTGTTAATGAACAATATTCTGTTTTAGCAGTTTTGGTCGTAAGNNAGCTGCACATGCCTGGCANTCGNTTTTCAAGCGGANGCTTCCAGNTATTNAAANTTCATCCTCTTAAGCGTGCTGGCTTTCTCCAGANNAGAAGANNGAANGNANAANNGACTCTGCGTNNTACCACGCTGGNNTANATGGTCGTATAGGAATTGCTGNNCNNGTTTANAACGNCTGANGGGAAAACCTGGCGTTACCCACTAATCGCTTGNNCAATCNNCNTNNNNACTGCTATAGG

>550-1

GNNNNNNNNNNNACGNANANGTCGCTTCTCGCTATCATATCATTCGAGCTCTTTTAGAAGGGGAGCTCACTCAAAGAGAGATAGCAGAGAAATACGGAGTCAGTATCGCACAAATTACCAGAGGATCTAATGCCCTTAAAGGATCAGATCCTCAATTTAAAGAGTTTTTACAAAAAGAGATCTGATCTTCTTTTGTAAAATACAAATAAGATTGAAAGTATTTGTATGCACGCGTTGTTAATGAACAAATATTCTGTTTTAGCAGTTTTGGTACGTAAGTATAGCTGCAGCATGCCATGCAAATCAGCTTTTCAAGCTGATTGCTTCCAAGATATTCAAAAATTCATCCTCTTACAGCGTGCCTGGCTTTCTTTTGAAAGCTGGCGCTTATCTACTTGGCGATAGGCCTAATTAAGAAGCCTTTTATTTGATTAAGAGATGTTCTTATAGAAGTAAGAGCGTCTTTTTTGCGCAGGATTATTCTGTAAAAAAAAAAAAAAAAAAAAAAAAAAAAAAAAGTACTCTGCGTTGATACCACTGCTTGCCCTATAGTGAGTCGTATTAGGAATTCACTGGCCGTCGTTTTACAACGTCGTGACTGGGAAAACCCTGGCGTTACCCAACTTAATCGCCTTTGCNNNACATCCCNNTTTCGCCNGCTGGNGNAATAGCGAAGAGNNCCGCACCGATNGCNCTTTCAAACAGTNGNGTANCNGNANNGGNNNNNGGGNNCGGNAGGNGGG

>550-2

NNNNNNNNNNCGANGNAGATGTCGCTTCTCGCTATCATATCATTCGAGCTCTTTTAGAAGGGGAGCTCACTCAAAGAGAGATAGCAGAGAAATACGGAGTCAGTATCGCACAAATTACCAGAGGATCTAATGCCCTTAAAGGATCAGATCCTCAATTTAAAGAGTTTTTACAAAAAGAGATCTGATCTTCTTTTGTAAAATACAAATAAGATTGAAAGTATTTGTATGCCGCGTTGTTAATGAACAATATTCTGTTTAGCAGTTTTGGTACGTAAGTANAGCTGCAGCATGCCATGCAAATCAGNTTTTCAAGCNGATTGCTTCCAAGNTATTCAAAAGTTCATCCTCTTAGANCGTGCCTGGCTTTCTTTTGANNGCTNCGCTTATCTNCNNGGNNNTNGNCNAATTANAAGNNTTNTNTTGNTAAGANNNNTCTATNANNNNGACNGNCTTNTTNCGNNGATATCNNCGNNNTTTNNCANNNNTNNAANNNAGAATNGATGACGAACTNTGGACCNGNANACNNNNTNGNNGCNTNNNNNCCNNNNNNNNNNG

>550-4

NNNNNNNNNNNNANGNNNATGTCGCTTCTCGCTATCATATCATTCGAGCTCTTTTAGAAGGGGAGCTCACTCAAAGAGAGATAGCAGAGAAATACGGAGTCAGTATCGCACAAATTACCAGAGGATCTAATGCCCTTAAAGGATCAGATCCTCAATTTAAAGAGTTTTTACAAAAAGAGATCTGATCTTCTTTTGTGAAATACAAATAAGATTGAAAGTATTTGTATGCACGCGTTGTTAATGAACAAATATTCTGTTTTAGCAGTTTTGGTACGTAAGTATAGCTGCAGCATGCCATGCAAATCAGCTTTTCAAGCTGATTGCTTCCAAGNTATTCAAAAATTCATCCTCTTACAGCGTGCCTGGCTTTCTTTTGAGAGCTGGCGCTTATCTACTTGGCGATAGGCCTAATTANGAAGCCTTTTATTTGATTAAGAGATGTTCTTATAGAAGTAAGAGCGTCTTTAAAAAAAAAAAAAAAAAAAAAAAAAAAAAAAANGTACTCTGCGTTGATACCACTGCTTGCCCTATAGTGAGTCGTATTAGGAATTCACTGGCNGTCGTTTTACAACGTNNTTGACTGGGAAAAACCCTGGNGGTTACCNCNNCTTAATCGNCCTTGCCAGCACATTCCCCCCTTTCCCCCANCCTGGNNNTNNNTAGNCGGAANNGGGCACAGCCACNGGATCCGCCNNTTNCCNNANNNNNNTGGGNNNNNNGCNNNGNAAG

>550-6

NNNNNNNNNNNNGACGNNNATGTCGCTTCTCGCTATCATATCATTCGAGCTCTTTTAGAAGGGGAGCTCACTCAAAGAGAGATAGCAGAGAAATACGGAGTCAGTATCGCACAAATTACCAGAGGATCTAATGCCCTTAAAGGATCAGATCCTCAATTTAAAGAGTTTTTACAAAAAGAGATCTGATCTTCTTTTGTAAAATACAAATAAGATTGAAAGTATTTGTATGCACGCGTTGTTAATGAACAAATATTCTGTTTTAGCAGTTTTGGTACGTAAGTATAGCTGCAGCATGCCATGCAAATCAGCTTTTCAAGCTGATTGCTTCCAAGATATTCAAAAATTCATCCTCTTACAGCGTGCCTGGCTTTCTTTTGAAAGCTGGCGCTTATCTACTTGGCGATAGGCCTAATTAAGAAGCCTTTTATTTGATTAAGAGATGTTCTTATAGAAGTAAGAGCGTCTTTTTTTGCGCAGGATTATTCTGTCGCCAGTTGAAAAAAAAAAAAAAAAAAAAAAAAAAAAAAGTACTCTGCGTTGATACCACTGCTTGCCCTATAGTGAGTCGTATTAGGAATTCACTGGCCGTCGTTTTACAACGTCGTGACTGGGAAAACCCTGGCGTTACCCAACTTAATCGCCTTGCAGCACATCGCCCTTTCGCCAGCTGGCGTAATAGCGAAGAGNCCNGCACNNATCGCCCTTCCCAACAGTTGCGCAGCCTGAATGGCGAATGGCGGCCTGATGCGGTATTTTGCTCCTNNCGCATNNGNGNCGGNATTTCACCCNGCNTATGGGGGCCCTCTTCGGTACAATCTG

>550-10

GNNNNNNNNNNNANGTAAGATGTCGCTTCTCGCTATCATATCATTCGAGCTCTTTTAGAAGGGGAGCTCACTCAAAGAGAGATAGCAGAGAAATACGGAGTCAGTATCGCACAAATTACCAGAGGATCTAATGCCCTTAAAGGATCAGATCCTCAATTTAAAGAGTTTTTACAAAAAGAGATCTGATCTTCTTTTGTAAAATACAAATAAGATTGAAAGTATTTGTATGCACGCGTTGTTAATGAACAAATATTCTGTTTTAGCAGTTTTGGTACGTAAGTATAGCTGCAGCATGCCATGCAAATCAGCTTTTCAAGCTGATTGCTTCCAAGATATTCAAAANTTCATCCTCTTACAGCGTGCCTGGCTTTCTTTTGANNGCTGGCGCTTATCTACGTGGCGATAGGCCTAATTAAGAAGCCTTGTATTTGATTAAGAGATGTTCTTATAGAAGTAAGAGCGTCTTTTTTGCGCAGGATTATTCTAAAAAAAAAAAAAAAAAAANGAAAAAAAAAAAAAAANTAATAAAGAAAAAAAAANGAAAAAAAAAAAAAAAGTACTCTGCGTTGATACCACTGCTTGCCCNATAGGGAGTCGNNTNNGNAATTCACTGGGCGNGNTTTGACANNNGCGTGACTGGGAAAACCCTGGGNGTTGCCCAACTTAATCGCCTTGCAGCACATCCCCCCTTTCGCCAGCNGGGNGNAANANCGAAAAAGGCCCCGNNCCGAATCGCCCTTTCNAAAANNNNGCCCNANNNNNNNNG

>550-11

NNNNNNNNNNNNNNNNANGTAAGATGTCGCTTCTCGCTATCATATCATTCGAGCTCTTTTAGAAGGGGAGCTCACTCAAAGAGAGATAGCAGAGAAATACGGAGTCAGTATCGCACAAATTACCAGAGGATCTAATGCCCTTAAAGGATCAGATCCTCAATTTAAAGAGTTTTTACAAAAAGAGATCTGATCTTCTTTTGTAAAATACAAATAAGATTGAAAGTATTTGTATGCACGCGTTGTTAATGAACAAATATTCTGTTTTAGCAGTTTTGGTACGTAAGTATAGCTGCAGCATGCCATGCAAATCAGCTTTTCAAGCGGATTGCTTCCANGATATTCAAAAGTTCATCCTCTTACNNCGTGCCGGGCTTTGTTTTGAGGGCTGGCGCTGATCTACGTGGCGANAGGCCTAATTAGNAAGCCTTGTATGNGNNTAANAGATGTTCTTANGTNNTANGAGCGTCTTTTTTGCGCAGGATTATTCTGTCGCCAGTTTTNTCTGCCNAAAANAAAAAGGAAAAAAAAAAAGAAAAGTGCTCTGCGTTGATACCACTGCCTGCCCCATAGTGAGTCGNATTANGAATTCACNGGCCGTCGTTTNACAACGNCTTGATTGGGAAAAACCCNGGCGTTAACCGCNNNNNTNGCCTTGNNGAANTTACCATTNNNGNNNGNNAGCGTANTATACTANATGTGNTNNNNAAGACC

>3000-2

NNNNNNNNNNNNANGTAAGATGTCGCTTCTCGCTATCATATCATTCGAGCTCTTTTAGAAGGGGAGCTCACTCAAAGAGAGATAGCAGAGAAATACGGAGTCAGTATCGCACAAATTACCAGAGGATCTAATGCCCTTAAAGGATCAGATCCTCAATTTAAAGAGTTTTTACAAAAAGAGATCTGATCTTCTTTTGTAAAATACAAATAAGATTGAAAGTATTTGTATGCACGCGTTGTTAATGAACAAATATTCTGTTTTAGCAGTTTTGGTACGTAAGTATAGCTGCAGCATGCCATGCAAATCAGCTTTTCAAGCTGATTGCTTCCAAGATATTCAAAAATTCATCCTCTTACAGCGTGCCTGGCTTTCTTTTGAAAGCTGGCGCTTATCTACTTGGCGATAGGCCTAATTAAGAAGCCTTTTATTTGATTAAGAGATGTTCTTATAGAAGTAAGAGCGTCTTTTTTGCGCAGGATTATTCTGTCGCCAGTTTTTTCTATGATTTTAACACTATAATTTTATGGAGAAAAGATGTTCAAACATAAACATCCTTTTGGGGGAGCGTTCCTTCCCGAAGAACTATTAGCCCCTATACAGAATCTAAAAGCGGAATGGGAGATTCTCAAAACTCAGCAAAGTTTTTTATCTGAACTAGATTGTATTTTGAAAAACTATGCGGGGAGACAAACTCCTCTGACTGAAGTTAAGAATTTTGCTCGAGCTATTGATGGCCCTAGAGTATTTCTTAAACGCGAAGATCTTTTGCATACAGGAGCACATAAACTGAATAATGCTCTTGGTCAGTGTTTGCTTGCTAAATATCTTGGGAAAACACGTGTTGTAGCTGAAACAGGTGCGGGACAACATGGAGTAGCAACAGCAACAGCGTGTGCTTATCTAGGATTAGATTGTGTAGTATACATGGGGAGCAAAAGATGTGGAACGACAGAAACCAAATGTAGAGAAAATGCGCTTTTTAGGTGCTGAAGTCGTTTNNGTAACAAAAGGATCTTNNGGNNNTCAANNATGCAGTTAATCAAGCTCTACANGATGGGGCAACAACCNNNNCATTTACTCCNTNTNGGCTTAGGATCGGCCTTAGGACCTTTACCTTATCCCGANNNCGTTCNAATTTTTCAGTCGNTTNNAANNNNNNNNNNGNAANNNNNNAATCCNNNNANNNGGNNGNANNNNNNNNNGGAATTNNNGATNNNNNNNNTCC

>3000-7

NNNNNNNNNNNNACGTAAGATGTCGCTTCTCGCTATCATATCATTCGAGCTCTTTTAGAAGGGGAGCTCACTCAAAGAGAGATAGCAGAGAAATACGGAGTCAGTATCGCACAAATTACCAGAGGATCTAATGCCCTTAAAGGATCAGATCCTCAATTTAAAGAGTTTTTACAAAAAGAGATCTGATCTTCTTTTGTAAAATACAAATAAGATTGAAAGTATTTGTATGCACGCGTTGTTAATGAACAAATATTCTGTTTTAGCAGTTTTGGTACGTAAGTATAGCTGCAGCATGCCATGCAAATCAGCTTTTCAAGCTGATTGCTTCCAAGATATTCAAAAATTCATCCTCTTACAGCGTGCCTGGCTTTCTTTTGAAAGCTGGCGCTTATCTACTTGGCGATAGGCCTAATTAAGAAGCCTTTTATTTGATTAAGAGATGTTCTTATAGAAGTAAGAGCGTCTTTTTTGCGCAGGATTATTCTGTCGCCAGTTTTTTCTATGATTTTAACACTATAATTTTATGGAGAAAAGATGTTCAAACATAAACATCCTTTTGGGGGAGCGTTCCTTCCCGAAGAACTATTAGCCCCTATACAGAATCTAAAAGCGGAATGGGAGATTCTCAAAACTCAGCAAAGTTTTTTATCGAACTAGATTGTATTTTGAAAAACTATGCGGGGAGACAAACTCCTCTGACTGAAGTTAAGAATTTTGCTCGAGCTATTGATGGCCCTAGAGTATTTCTTAAACGCGAAGATCTTTTGCATACAGGAGCACATAAACTGAATAATGCTCTTGGTCAGTGTTTGCTTGCTAAATATCTTGGGAAAACACGTGTTGTAGCTGAAACAGGTGCGGGACAACATGGAGTAGCAACAGCAACAGCGTGTGCTTATCTAGGATTAGATTGTGTAGTATACATGGGGAGCAAAAGATGTGGAACGACAGAAACCAAATGTAGAGAAAATGCGCTTTTTAGGTGCTGAAGTCGTTTCTGTAACAAAAGGATCTTGGGGGACTCAAAGATGCAGTTAATCAAGCTNNACAAGATGGGNCAACAACCNACTCATTTACTCACTATTNNNTTAGGATCGGCNTTNAGGACCTTTACCTTATCCCGNNNNCGTTNNATTTTTNCNGNCGNTTNNAANNGNTGAANNGGAANNNCAANNCCNNGC

>3000-8

NNNNNNNNNNNNGTAAGATGTCGCTTCTCGCTATCATATCATTCGAGCTCTTTTAGAAGGGGAGCTCACTCAAAGAGAGATAGCAGAGAAATACGGAGTCAGTATCGCACAAATTACCAGAGGATCTAATGCCCTTAAAGGATCACAAAAAAAAAAAAAAAAAAAAAAAAAAGTACTCTGCGTTGATACCACTGCTTGCCCTATAGTGAGTCGTATTAGGAATTCACTGGCCGTCGTTTTACAACGTCGTGACTGGGAAAACCCTGGCGTTACCCAACTTAATCGCCTTGCAGCACATCCCCCTTTCGCCAGCTGGCGTAATAGCGAAGAGGCCCGCACCGATCGCCCTTCCCAACAGTTGCGCAGCCTGAATGGCGAATGGCGCCTGATGCGGTATTTTCTCCTTACGCATCTGTGCGGTATTTCACACCGCATATGGTGCACTCTCAGTACAATCTGCTCTGATGCCGCATAGTTAAGCCAGCCCCGACACCCGCCAACACCCGCTGACGCGCCCTGACGGGCTTGTCTGCTCCCGGCATCCGCTTACAGACAAGCTGTGACCGTCTCCGGGAGCTGCATGTGTCAGAGGTTTTCACCGTCATCACCGAAACGCGCGAGACGAAAGGGCCTCGTGATACGCCTATTTTTATAGGTTAATGTCATGATAATAATGGTTTCTTAGACGTCAGGTGGCACTTTTCGGGGAAATGTGCGCGGAACCCCTATTTGTTTATTTTTCTAAATACATTCAAATATGTATCCGCTCATGAGACAATAACCCTGATAAATGCTTCAATAATATTGAAAAAGGAAGAGTATGAGTATTCAACATTTCCGTGTCGCCCTTATTCCCTTTTTTGCGGCATTTTGCCTTCCTGTTTTTGCTCACCCAGAAACGCTGGTGAAAGTAAAAGATGCTGAAGATCAGTTGGGTGCACGAGTGGGTTACATCGAACTGGATCTCAACAGCGGTAAGATCCTTGAAAGTTTTCGCCCCGAANAACGTTTTCCAATGATGAACACTTTTAAAGTTCTGCTATGTGGGNNNGGTATTATCCCGTATTGANNCNGGGCANNANCAACTCGNTNNCCGCATAACCTATTNNNAAAATGACTTGGTTGAATANNNACCNNNNNNNNAAANNCNNNTTACGNNTGGCTTGNNNNTAANAAANTTNNGNAAGGTCC

511652

>3000-18

NNNNNNNNGNNNACGTAAGNTGTCGCTTCTCGCTATCATATCATTCGAGCTCTTTTAGAAGGGGAGCTCACTCAAAGAGAGATAGCAGAGAAATACGGAGTCAGTATCGCACAAATTACCAGAGGATCTAATGCCCTTAAAGGATCAGATCCTCAATTTAAAGAGTTTTTACAAAAAGAGATCTGATCTTCTTTTGTAAAATACAAATAAGATTGAAAGTATTTGTATGCACGCGTTGTTAATGAACAAATATTCTGTTTTAGCAGTTTTGGTACGTAAGTATAGCTGCAGCATGCCATGCAAATCAGCTTTTCAAGCTGATTGCTTCCAAGATATTCAAAAATTCATCCTCTTACAGCGTGCCTGGCTTTCTTTTGAAAGCTGGCGCTTATCTACTTGGCGATAGGCCTAATTAAGAAGCCTTTTATTTGATTAAGAGATGTTCTTATAGAAGTAAGAGCGTCTTTTTTGCGCAGGATTATTCTGTCGCCAGTTTTTTCTATGATTTTAACACTATAATTTTATGGAGAAAAGATGTTCAAACATAAACATCCTTTTGGGGGAGCGTTCCTTCCCGAAGAACTATTAGCCCCTATACAGAATCTAAAAGCGGAATGGGAGATTCTCAAAACTCAGCAAAGTTTTTTATCTGAACTAGATTGTATTTTGAAAAACTATGCGGGGAGACAAACTCCTCTGACTGAAGTTAAGAATTTTGCTCGAGCTATTGATGGCCCTAGAGTATTTCTTAAACGCGAAGATCTTTTGCATACAGGAGCACATAAACTGAATAATGCTCTTGGTCAGTGTTTGCTTGCTAAATATCTTGGGAAAACACGTGTTGTAGCTGAAACAGGTGCGGGACAACATGGAGTAGCAACAGCAACAGCGTGTGCTTATCTAGGATTAGATTGTGTAGTATACATGGGGAGCAAAAGATGTGGAACGACAGAAACCAAATGTAGAGAAAATGCGCTTTTTAGGTGCTGAANTCGTTTCTGTAACAAAAGGATCTTGTGGACTCAAANNGNCAGTTAATCAAGNTCTACAAGATGGGNCAACAACANACTCATTTACTCNCTATTGCTTAGGATCGGCCTTAGGACCTTTACCTTATCCCGAANNCGTTCGATTTTTNCAGTCGGTTAAANNNNNTGAANNGAAAGNNNAATCCNTNNNNTTNGCGGNAANGAATCCNGAANTTCC

>3000-30

NNNNNNNNNNNANGTAAGATGTCGCTTCTCGCTATCATATCATTCGAGCTCTTTTAGAAGGGGAGCTCACTCAAAGAGAGATAGCAGAGAAATACGGAGTCAGTATCGCACAAATTACCAGAGGATCTAATGCCCTTAAAGGATCAGATCCTCAATTTAAAGAGTTTTTACAAAAAGAGATCTGATCTTCTTTTGTAAAATACAAATAAGATTGAAAGTATTTGTATGCACGCGTTGTTAATGAACAAATATTCTGTTTTAGCAGTTTTGGTACGTAAGTATAGCTGCAGCATGCCATGCAAATCAGCTTTTCAAGCTGATTGCTTCCAAGATATTCAAAAATTCATCCTCTTACAGCGTGCCTGGCTTTCTTTTGAAAGCTGGCGCTTATCTACTTGGCGATAGGCCTAATTAAGAAGCCTTTTATTTGATTAAGAGATGTTCTTATAGAAGTAAGAGCGTCTTTTTTGCGCAGGATTATTCTGTCGCCAGTTTTTTCTATGATTTTAACACTATAATTTTATGGAGAAAAGATGTTCAAACATAAACATCCTTTTGGGGGAGCGTTCCTTCCCGAAGAACTATTAGCCCCTATACAGAATCTAAAAGCGGAATGGGAGATTCTCAAAACTCAGCAAAGTTTTTTATCTGAGCTAGATTGTATTTTGAAAAACTATGCGGGGAGACAAACTCCTCTGACTGAAGTTAAGAATTTTGCTCGAGCTATTGATGGCCCTAGAGTATTTCTTAAACGCGAAGATCTTTTGCATACAGGAGCACATAAACTGAATAATGCTCTTGGTCAGTGTTTGCTTGCTAAATATCTTGGGAAAACACGTGTTGTAGCTGAAACAGGTGCGGGACAACATGGAGTAGCAACAGCAACAGCGTGTGCTTATCTAGGATTAGATTGTGTAGTATACATGGGAGCAAAAGATGTGGAACGACAGAAACCAAATGTAAAGAAAATGCGCTTTTTAGGTGCTGAANTCGTTTCTGTAACAAAAGGANCTTNNGNACTCAAAGATGCAGTTAATCAAGNTCTACAAGATTGGGCAACAACNNNNTCATTTACTNNNTATTGCTTAGGATCGGCCTTAGGACCTTTACCTTATCCCGAANNCGTTCGATTTTTTCAGTCGGTTNAANNNNNTGAANNGAAAANNNAANNCCNNGNNNTTNNNGGAANNANTNNNGANNNNNNNNTNNNNNNNNNCNAANG

>250-1 reverse complement

CANNNGCTNNNNANCANGAATACNGCAACAGGTNCCATTCNCCNCCCATGCTGCGCAACT

GTTGGGAAGGACGATCGGTGCGGGCCTTTTCGCTATTACGCCAGCTGGCGAAAGGGGGAT

GTGCTGCAAGGCGATTAAGTTGGGTAACGCCAGGGTTTTCCCAGTCACGACGTTGTAAAA

CGACGGCCAGTGAATTCCTAATACGACTCACTATAGGGCAAGCAGTGGTATCAACGCAGA

GTACTTTTTTTTTTTTTTTTTTTTTTTTTTTTTTCATTGAGGATCTGATCCTTTAAGGGC

ATTAGATCCTCTGGTAATTTGTGCGATACTGACTCCGTATTTCTCTGCTATCTCTCTTTG

AGTGAGCTCCCCTTCTAAAAGAGCTCGAATGATATGATAGAGAGAAGCGACATCTACNTN

NNNNNNNNNNNNNN

>250-2 reverse complement

ANCNGACGTNTANGAAACCCATTNNTATCCNGNCATTAACCTATAAAAATAGGCGGTNTC

NCNNGGCCCTTTCGTCTCGCGCGTTTCGGTGATGACGGTGAAAACCTCTGACACATGCAG

CTCCCGGAGACGGTCACAGCTTGTCTGTAAGCGGATGCCGGGAGCAGACAAGCCCGTCAG

GGCGCGTCAGCGGGTGTTGGCGGGTGTCGGGGCTGGCTTAACTATGCGGCATCAGAGCAG

ATTGTACTGAGAGTGCACCATATGCGGTGTGAAATACCGCACAGATGCGTAAGGAGAAAA

TACCGCATCAGGCGCCATTCGCCATTCAGGCTGCGCAACTGTTGGGAAGGGCGATCGGTG

CGGGCCTCTTCGCTATTACGCCAGCTGGCGAAAGGGGGATGTGCTGCAAGGCGATTAAGT

TGGGTAACGCCAGGGTTTTCCCAGTCACGACGTTGTAAAACGACGGCCAGTGAATTCCTA

ATACGACTCACTATAGGGCAAGCAGTGGTATCAACGCAGAGTACTTTTTTTTTTTTTTTT

TTTTTTTTTTTTAATTGAGGATCTGATCCTTTAAGGGCATTAGATCCTCTGGTAATTTGT

GCGATACTGACTCCGTATTTCTCTGCTATCTCTCTTTGAGTGAGCTCCCCTTCTAAAAGA

GCTCGAATGATATGATAGCGAGAAGCGACANCTTACNTNNNNNNNNNNNNN

>250-4 reverse complement

GGNGNCGGTNACACNNTATCCCGTAAGCGGAAGCCAGNCNCNCCCNTCCGGGCCCGTCAG

CGGGTGTTGGCGGTTGTCGGGGCTGGCTTAACTATGCGGCATCAGAGCAGATTGTACTGA

GAGTGCACCATATGCGGTGGGAAATACCGCACAGATGCGTAAGGAGAAAATACCGCATCA

GGCGCCATTCGCCATTCAGGCTGCGCAACTGTTGGGAAGGGCGATCGGTGCGGGCCTTTT

CGCTATTACGCCAGCTGGCGAAAGGGGGATGTGCTGCAAGGCGATTAAGTTGGGTAACGC

CAGGGTTTTCCCAGTCACGACGTTGTAAAACGACGGCCAGTGAATTCCTAATACGACTCA

CTATAGGGCAAGCAGTGGTATCAACGCAGAGTACTTTTTTTTTTTTTTTTTTTTTTTTTT

TTTTTTCTTTTTTTTTTGAGGATCTGATCCTTTAAGGGCATTAGATCCTCTGGTAATTTG

TGCGATACTGACTCCGTATTTCTCTGCTATCTCTCTTTGAGTGAGCTCCCCTTCTAAAAG

AGCTCGAATGATATGATAGCGAGAAGCGACATCNTACNTNNNNNNNNNNNNN

>250-5 reverse complement

GGNTTNNNNNNCNGGNTTNNGNNNNNNNGNNNNNTTTTTTTANNNNNNNNCCNNNCGTAA

NNNNNNTTTCNNGGANNNNGGAGNNNNNAACCAAGTNNNTTTGANAATAGGNNTTGNGGN

GACCGNNTTGNTTTTGCCCGGCGTCAANACGGGNNNANNNCNNNCCNNTTAGCNNAACTT

TAAAANNGNTCATCATTGGAAAACNNTTNTTCGGGGCGAAAACTTTCAAGGATCTTACCG

CTGTTGAGATCCAGTTCGATGTAACCCNNTCGTGCACCCAACTGATCTTCAGCATCTTTT

ACTTTCACCAGCGTTTCTGGGTGAGCAAAAACAGGAAGGCAAAATGCCGCAAAAAAGGGA

ATAAGGGCGACACGGAAATGTTGAATACTCATACTCTTCCTTTTTCAATATTATTGAAGC

ATTTATCAGGGTTATTGTCTCATGAGCGGATACATATTTGAATGTATTTAGAAAAATAAA

CAAATAGGGGTTCCGCGCACATTTCCCCGAAAAGTGCCACCTGACGTCTAAGAAACCATT

ATTATCATGACATTAACCTATAAAAATAGGCGTATCACGAGGCCCTTTCGTCTCGCGCGT

TTCGGTGATGACGGTGAAAACCTCTGACACATGCAGCTCCCGGAGACGGTCACAGCTTGT

CTGTAAGCGGATGCCGGGAGCAGACAAGCCCGTCAGGGCGCGTCAGCGGGTGTTGGCGGG

TGTCGGGGCTGGCTTAACTATGCGGCATCAGAGCAGATTGTACTGAGAGTGCACCATATG

CGGTGTGAAATACCGCACAGATGCGTAAGGAGAAAATACCGCATCAGGCGCCATTCGCCA

TTCAGGCTGCGCAACTGTTGGGAAGGGCGATCGGTGCGGGCCTCTTCGCTATTACGCCAG

CTGGCGAAAGGGGGATGTGCTGCAAGGCGATTAAGTTGGGTAACGCCAGGGTTTTCCCAG

TCACGACGTTGTAAAACGACGGCCAGTGAATTCCTAATACGACTCACTATAGGGCAAGCA

GTGGTATCAACGCAGAGTACTTTTTTTTTTTTTTTTTTTTTTTTTTTTTTTAAATTGAGG

ATCTGATCCTTTAAGGGCATTAGATCCTCTGGTAATTTGTGCGATACTGACTCCGTATTT

CTCTGCTATCTCTCTTTGAGTGAGCTCCCCTTCTAAAAGAGCTCGAATGATATGATAGCG

AGAAGCGACANCTTACNNNNNNNNNNNNNN

>250-6 reverse complement

GCCTTNGTAAGAGGTTTTTNNNNANNGGNNNNTACTCAACCAAGTCANTTTNNGAATANG

GTATGCNNNNACCGAGTTGNTNTTGCCCGGCGTCAATACGGGATAANNCCNNNCCANATA

GCAGAACTTTAAAAGTGCTCATCATTGGAAAACGTTTTTCNGGGGGAAAANTTTCAAGGA

TCTTACCGCTGTTGAGATCCAGTTCGATGTAACCCACTCGTGCACCCAACTGATCTTCAG

CATCTTTTACTTTCACCAGCGTTTCTGGGTGAGCAAAAACAGGAAGGCAAAATGCCGCAA

AAAAGGGAATAAGGGCGACACGGAAATGTTGAATACTCATACTCTTCCTTTTTCAATATT

ATTGAAGCATTTATCAGGGTTATTGTCTCATGAGCGGATACATATTTGAATGTATTTAGA

AAAATAAACAAATAGGGGTTCCGCGCACATTTCCCCGAAAAGTGCCACCTGACGTCTAAG

AAACCATTATTATCATGACATTAACCTATAAAAATAGGCGTATCACGAGGCCCTTTCGTC

TCGCGCGTTTCGGTGATGACGGTGAAAACCTCTGACACATGCAGCTCCCGGAGACGGTCA

CAGCTTGTCTGTAAGCGGATGCCGGGAGCAGACAAGCCCGTCAGGGCGCGTCAGCGGGTG

TTGGCGGGTGTCGGGGCTGGCTTAACTATGCGGCATCAGAGCAGATTGTACTGAGAGTGC

ACCATATGCGGTGTGAAATACCGCACAGATGCGTAAGGAGAAAATACCGCATCAGGCGCC

ATTCGCCATTCAGGCTGCGCAACTGTTGGGAAGGGCGATCGGTGCGGGCCTCTTCGCTAT

TACGCCAGCTGGCGAAAGGGGGATGTGCTGCAAGGCGATTAAGTTGGGTAACGCCAGGGT

TTTCCCAGTCACGACGTTGTAAAACGACGGCCAGTGAATTCCTAATACGACTCACTATAG

GGCAAGCAGTGGTATCAACGCAGAGTACTTTTTTTTTTTTTTTTTTTTTTTTTTTTTTTT

TTTCAAATTGAGGATCTGATCCTTTAAGGGCATTAGATCCTCTGGTAATTTGTGCGATAC

TGACTCCGTATTTCTCTGCTATCTCTCTTTGAGTGAGCTCCCCTTCTAAAAGAGCTCGAA

TGATATGATAGCGAGAAGCGACANCTNCGTNNNNNNNNNC

>250-7 reverse complement

CNNNNNNTTANNCCNNNNNAAATAGGCGTATCNNNNATNCCCTTTCGTCTTNNNACGTTT

CGGNGATGACGGTNAAAACCTNTGACACATGCAGCTCCCGGAGACGGTCACAGCTTGTCT

GTAAGCGGATGCCGGGAGCAGACAAGCCCGTCAGGGCGCGTCAGCGGGTGTTGGCGGGTG

TCGGGGCTGGCTTAACTATGCGGCATCAGAGCAGATTGTACTGAGAGTGCACCATATGCG

GTGTGAAATACCGCACAGATGCGTAAGGAGAAAATACCGCATCAGGCGCCATTCGCCATT

CAGGCTGCGCAACTGTTGGGAAGGGCGATCGGTGCGGGCCTCTTCGCTATTACGCCAGCT

GGCGAAAGGGGGATGTGCTGCAAGGCGATTAAGTTGGGTAACGCCAGGGTTTTCCCAGTC

ACGACGTTGTAAAACGACGGCCAGTGAATTCCTAATACGACTCACTATAGGGCAAGCAGT

GGTATCAACGCAGAGTACTTTTTTTTTTTTTTTTTTTTTTTTTTTTTTGTTTTTTTTTAA

TTGAGGATCTGATCCTTTAAGGGCATTAGATCCTCTGGTAATTTGTGCGATACTGACTCC

GTATTTCTCTGCTATCTCTCTTTGAGTGAGCTCCCCTTCTAAAAGAGCTCGAATGATATG

ATAGCGAGAAGCGACATCTTACGTCNNNNNNNNNN

>400-1 reverse complement

NTNAGATCGTGCNCNCANGCANCNTGTTNTTNCCNTCACACNCCAATACAGAGCANACCA

NTGATNACCNCNTCNCCCTCACTCTNCNGCAANCCNNGGTACNCCCNCCGNGTCCCTTNT

TTTTTTTTCGCTTCTCTTTTCCNAGGAAGCAATCCGCTTGAAAAGCTGATTTGCATGGCA

TGCTGCAGNTATACTTACGTACCAAAACTGCTAAAACAGAATATTTGTTCATTAACAACG

CGTGCATACAAATACTTTCAATCTTATTTGTATTTTACAAAAGAAGATCAGATCTCTTTT

TGTAAAAACTCTTTAAATTGAGGATCTGATCCTTTAAGGGCATTAGATCCTCTGGTAATT

TGTGCGATACTGACTCCGTATTTCTCTGCTATCTCTCTTTGAGTGAGCTCCCCTTCTAAA

AGAGCTCGAATGATATGATAGCGAGAAGCGACATCTTACNTNNNNNNNNNNNN

>400-2 reverse complement

TTTNNNANNCNNNTNNCCGGTGTANTACCCNNNATGTTANGGNACNTCCGCATCANGNCC

ANTCNCCNTCAGAGTGNCANNTGTTGGGAAGGGCGATCGGTGCGGGCCTCTTCGTTATTA

CGCCAGCTGGCGAAAGGGGGATGNGANCAAGGNNANTAAGTTNNGTNANNGCAGGGTTTT

CCCAGTCACGCGTTGTNNAACGACNNCCAGTNAATTCCTAATACGACTCACTATNNGGCA

AGCAGTGGTATNNACGCAGAGTACNTTTTTTTTTTCNTTTTTTTTTTTTTTTTTTCTATG

GCATGCTGCAGCTATACTTACGTACCAAAACTGCTAAAACAGAATANTNNTCATTAACAA

CGCGTGCATACAAATACTTTCAATTTATTTGTATTTTACAAAAGAAGATCAGATCTCTTT

TTGTAAAAACTCTTTAAATTGAGGATCTGATCCTTTAAGGGCATTAGATCCTCTGGTAAT

TTGTGCGATACTGACTCCGTATTTCTCTGCTATCTCTCTTTGAGTGAGCTCCCCTTCTAA

AAGAGCTCGAATGATATGATAGCGAGAAGCGACATNNNNCNTNNNNNNNNNNNNNNN

>400-4 reverse complement

NNNNNNNGAAACNNCTNTGACCAGAGATTACNCCNNNCTTGGCNNACNNANAGCATGTCN

CTNCTCGCNNTCANTTCATTCGCGCTCNCCNTTNACCATTGCNNNCGCAAAGAGGTGGGC

AGGGCGATNCGNGGGCGCTTTTTCGCTATTACCCCAGCNGGCGAAAGGGGGATGTGCTGC

AAGGCGATTAAGTGGGTAACNNNAGGGTTTTCCCAGTCCATGACGTCGTAAAANCACGGC

CNGTGAATTCCTAATACGACTCCCTATAGGGCAAGCAGTGGTATCAACGCAGAGTACTTC

TTTTTTTTTTTTTTTTTTTTTTTTTGATCTTGGAAGCAATCAGCTTGAAAAGCTGATTTG

CATGGCATGCTGCAGCTATACTTACGTACCAAAACTGCTAAAACAGAATATTTGTTCATT

AACAACGCGTGCATACAAATACTTTCAATCTTATTTGTATTTTACAAAAGAAGATCAGAT

CTCTTTTTGTAAAAACTCTTTAAATTGAGGATCTGATCCTTTAAGGGCATTAGATCCTCT

GGTAATTTGTGCGATACTGACTCCGTATTTCTCTGCTATCTCTCTTTGAGTGAGCTCCCC

TTCTAAAAGAGCTCGAATGATATGATAGCGAGAAGCGACATCTTACNTCNNNNNNNNNN

>400-5 reverse complement

TGNGNCGNAGGNNTCCNNCNNNANNTTNNCNNNNNGNNTCNAGCNNANANCNCNCNTCAN

NNCANNNACNTCAGGNGNNCACNNTTNGANGGGCNNTNNNTCNGCNTTTCNNNATNNCGC

CAGNGNGAAAGGGGGATGTGCTGCAAGGNNATTAAGTNGGGTAACGCCAGGGTTNNCCCA

GTCCNANNTGTANNAANNCGGCCAGTGNNTCCTNNNACGANNNACTATNNGNNAGCAGNG

GNNNCANNCCNAGTACNTTTTTTTTTCTTTTNTTTTNTTTTTTGGCAATCAGCTGAAAAG

CTGATTTGCAGGCATGNNCAGCTATACTTACGTACCAAAATGCTAAAACAGAATATTGTT

CATTAACAACGCGTGCATACAAATACTTTCAATCTTATTTGTATTTTACAAAAGAAGATC

AGATCTCTTTTTGTAAAAACTCTTTAAATTGAGGATCTGATCCTTTAAGGGCATTAGATC

CTCTGGTAATTTGTGCGATACTGACTCCGTATTTCTCTGCTATCTCTCTTTGAGTGAGCT

CCCCTTCTAAAAGAGCTCGAATGATATGATAGCGAGAAGCGACATCTTACNTNNNNNNNN

NN

>400-6 reverse complement

NTNGTNNTNAANCNGATNCANAGAGCAGACCAAGCCCGTCAGGNCGCNTCAGCGGGNGTT

GGCGGGNGTCGGGGCTGGTTTAANTATNCGGCATCAGAGCAGATTGTACTGAGAGTGCAC

CATATGCGGTGTGAAATACCGCACAGATGCGTAAGGAGAAAATACCGCATCAGGCGCCAT

TCGCCATTCAGGCTGCGCAACTGTTGGGAAGGGCGATCGGTGCGGGCCTCTTCGCTATTA

CGCCAGCTGGCGAAAGGGGGATGTGCTGCAAGGCGATTAAGTTGGGTAACGCCAGGGTTT

TCCCAGTCACGACGTTGTAAAACGACGGCCAGTGAATTCCTAATACGACTCACTATAGGG

CAAGCAGTGGTATCAACGCAGAGTACTTTTTTTTTTTTTTTTTTTTTTTTTTTTTTGCAT

GGCATGCTGCAGCTATACTTACGTACCAAAACTGCTAAAACAGAATATTTGTTCATTAAC

AACGCGTGCATACAAATACTTTCAATCTTATTTGTATTTTACAAAAGAAGATCAGATCTC

TTTTTGTAAAAACTCTTTAAATTGAGGATCTGATCCTTTAAGGGCATTAGATCCTCTGGT

AATTTGTGCGATACTGACTCCGTATTTCTCTGCTATCTNTCTTTGAGTGAGCTCCCCTTC

TAAAAGAGCTCGAATGATATGATAGCGAGAAGCGACATCNNCNTNGNNNNNNNNNNNN

>400-10 reverse complement

CNNNNTTTTTNNNNTTNNCCCCNGNNNTTNNNNGNGGNNCCAAAAACCNNNNNNGGCCAA

AANTCCGCCNAAAAAAGGNNATAANNGGNNNNCCAGGAAAANTNTNGAATCCTCCTTCCT

CTTCCTTTTTCCCATNNNTTTGGAGGNNTTTNNCCGGGGTTNTGTTCTCATGNNNCNGAT

ACNTNTTTGANNNNNTTNGGANNAANNACCAAANTGGGGTTNCCGCGCACATTTCCCCGA

AAAAGTGCCACCTGACGTCTAAGAAACCCATTATTATCATGACATTAACCTATAAAAATA

GGCGTATCACGAGGCCCTTTCGTCTCGCGCGTTTCGGTGATGACGGTGAAAACCTCTGAC

ACATGCAGCTCCCGGAGACGGTCACAGCTTGTCTGTAAGCGGATGCCGGGAGCAGACAAG

CCCGTCAGGGCGCGTCAGCGGGTGTTGGCGGGTGTCGGGGCTGGCTTAACTATGCGGCAT

CAGAGCAGATTGTACTGAGAGTGCACCATATGCGGTGTGAAATACCGCACAGATGCGTAA

GGAGAAAATACCGCATCAGGCGCCATTCGCCATTCAGGCTGCGCAACTGTTGGGAAGGGC

GATCGGTGCGGGCCTCTTCGCTATTACGCCAGCTGGCGAAAGGGGGATGTGCTGCAAGGC

GATTAAGTTGGGTAACGCCAGGGTTTTCCCAGTCACGACGTTGTAAAACGACGGCCAGTG

AATTCCTAATACGACTCACTATAGGGCAAGCAGTGGTATCAACGCAGAGTACTTTTTTTT

TTTTTTTTTTTTTTTTTTTTTAGCTATACTTACGTACCAAAACTGCTAAAACAGAATATT

TGTTCATTAACAACGCGTGCATACAAATACTTTCAATCTTATTTGTATTTTACAAAAGAA

GATCAGATCTCTTTTTGTAAAAACTCTTTAAATTGAGGATCTGATCCTTTAAGGGCATTA

GATCCTCTGGTAATTTGTGCGATACTGACTCCGTATTTCTCTGCTATCTCTCTTTGAGTG

AGCTCCCCTTCTAAAAGAGCTCGAATGATATGATAGCGAGAAGCGACATCNNCNTNNNNN

NNNNNC

>450-1 reverse complement

NNNCNNNCCGAGAATACGAAATTCNNCTCAAAAAAGAGCACCTCNNAAGNNGGAACCCCN

NGCACTTTGTTCATTTCTCCTNNNGTAAATTCNGCTTTCAAANCGAAGCCAGGCGCGCTG

TAAGAGGATGAATTTTTGAATATCTTGGAAGCAATCAGCTTGAAAAGCTGATTTGCATGG

CATGCTGCAGCTATACTTACGTACCAAAACTGCTAAAACAGAATATTTGTTCATTAACAA

CGCGTGCATACAAATACTTTCAATCTTATTTGTATTTTACAAAAGAAGATCAGATCTCTT

TTTGTAAAAACTCTTTAAATTGAGGATCTGATCCTTTAAGGGCATTAGATCCTCTGGTAA

TTTGTGCGATACTGACTCCGTATTTCTCTGCTATCTNTCTTTGAGTGAGCTCCCCTTCTA

AAAGAGCTCGAATGATATGATAGCGAGAAGCGACATCNNCNTNNNNNNNNNNNNC

>450-2 reverse complement

ANNTNCNNNNTNTNNCGCCCCTTTCCTTTTGTTNTTCCCCCCCACTNANAAAAAGCCATC

CANGCNCGAAGCGAGGCGATGAATTTTAAATCTCCNTACAACCTCNCACCAAATTCNCGC

GATAAACNTGCCTTCCACGACATGCACCAGCTCCNCAACCGAACCAAAACTGNTANAACA

GAATATTTGTTCATTAACAACGCGTGCATACAAATACTTTCAATCTTATTTGTATTTTAC

AAAAGAAGATCAGATCTCTTTTTGTAAAAACTCTTTAAATTGAGGATCTGATCCTTTAAG

GGCATTAGATCCTCTGGTAATTTGTGCGATACTGACTCCGTATTTCTCTGCTATCTNNCT

TTGAGTGAGCTCCCCTTCTAAAAGAGCTCGAATGATATGATAGCGAGAAGCGACATCNNN

CNNNNNNNNNNNNNN

>450-3 reverse complement

TNNNTNNGANNNNNNNNNNNTNCTNATCGNNNCGNNNNNNTNTNNNNANNNNNTNNNNNN

NANNNNNNNANNNGNNNNNNTNCNCNCCACCANNNNNNCNCNNNNGNNCCNCCNNGCGNT

NNNNNNANCCCCNNNNNNNCNNNNNNNNNNCNCNNNNAAANANANGGGGTNCNNGGGGCC

CCCTTTTNTNNCNNNNNTTTNNGGGNGANNNNGGGAAAANNCTTTNNCNCNNNCNNTCCC

CNNGGAGNGNNCNNNNNTTTTTTTNAAGGGNNNCCNGGGGAANNNNAACCCNCCNNGGGG

NNTTNNNNNGNNTTTNNGGGGNTTGGGGNNNNTTTNANNNTNNNGNCCCNNANCCNNNNN

TTNNNNNNGGGCCCCCCCTNNGGGGGGNAAAACCCCCCCCAAGNGNTANGNNAAAAAAAC

NCNCCCGGGGCCCCTTNCCCTTNTGGGGGGNNAANNTTTNGGANNGGGGNNNNGGGGGGG

CCNTTTTTTTTTTTNCCCCCCCGGNNAAAAGGGGGGNNTNTCCCAAGGGNNNAAATTGGG

GGAACCCCCGGGTTTTCCCCCCCCCCCGGTTTAAAAAAAGGGCCCCGGAATTTCTTTTTT

GGCCCCCCTTTGGGGCAACCCGGGGGTTAACCCCGGGGAACTTTTTTTTTTTTTTTTTTT

TTTTTTAATNTTTTTTTTTTTTTTTTTTTTTTTTTTTTTTTTTTTTTTTTTTTTTTTTTT

TTTTTTTTTTTTTTTTTTTTTTTTTTTTTTTTTTTTTTTTTTTTTTTTTTGCTGCAGCTA

TACTTACGTACCAAAACTGCTAAAACAGAATATTTGTTCATTAACAACGCGTGCATACAA

ATACTTTCAATCTTATTTGTATTTTACAAAAGAAGATCAGATCTCTTTTTGTAAAAACTC

TTTAAATTGAGGATCTGATCCTTTAAGGGCATTAGATCCTCTGGTAATTTGTGCGATACT

GACTCCGTATTTCTCTGCTATCTCTCTTTGAGTGAGCTCCCCTTCTAAAAGAGCTCGAAT

GATATGATAGCGAGAAGCGACATCTTACGTCNNNNNNNNNN

>450-5 reverse complement

GNNNNCNGAGACGGTCCACAGNTTGTCTGTAAGCGGATGCCGGGAGCAGACAAGCCCGTC

AGGGCGCGTCAGCGGGTGTTGGCGGGTGTCGGGGCTGGCTTAACTATGCGGCATCAGAGC

AGATTGTACTGAGAGTGCACCATATGCGGTGTGAAATACCGCACAGATGCGTAAGGAGAA

AATACCGCATCAGGCGCCATTCGCCATTCAGGCTGCGCAACTGTTGGGAAGGGCGATCGG

TGCGGGCCTCTTCGCTATTACGCCAGCTGGCGAAAGGGGGATGTGCTGCAAGGCGATTAA

GTTGGGTAACGCCAGGGTTTTCCCAGTCACGACGTTGTAAAACGACGGCCAGTGAATTCC

TAATACGACTCACTATAGGGCAAGCAGTGGTATCAACGCAGAGTACTTTTTTTTTTTTTT

TTTTTTTTTTTTCTGCTTTCAAAAGAAAGCCAGGCACGCTGTAAGAGGATGAATTTTTGA

ATATCTTGGAAGCAATCAGCTTGAAAAGCTGATTTGCATGGCATGCTGCAGCTATACTTA

CGTACCAAAACTGCTAAAACAGAATATTTGTTCATTAACAACGCGTGCATACAAATACTT

TCAATCTTATTTGTATTTTACAAAAGAAGATCAGATCTCTTTTTGTAAAAACTCTTTAAA

TTGAGGATCTGATCCTTTAAGGGCATTAGATCCTCTGGTAATTTGTGCGATACTGACTCC

GTATTTCTCTGCTATCTCTCTTTGAGTGAGCTCCCCTTCTAAAAGAGCTCGAATGATATG

ATAGCGAGAAGCGACATCTTACNNNGNNNNNNNNN

>450-6 reverse complement

AGNNCTGTNNNTGAGAAGTGCCCCCNNNNNCNGTGTGAAATGCCGGCACAGATNTCNNAA

AGACGANNNNNCCGCATCAGGNCGCCATTGNNCANNCAGNGTGCGCAACTNNTNGNACGG

GCGATCGGCGCGGGCCNNNTCGCTATTACGCCANNNGGCGAAAGGGGGATGTGCTGCAAG

GCGATTAAGTTGGGTAACGCCAGGGTTTTCCCAGTCACGACGTTGTAAAACGACGGCCAG

TGAATTCCTAATACGACTCACTATAGGGCAAGCAGTGGTATCAACGCAGAGTACTTTTTT

TTTTTTTTTTTTTTTTTTTTTTTAAGCGCCAGCTTTCAAAAGAAAGCCAGGCACGCTGTA

AGAGGATGAATTTTTGAATATCTTGGAAGCAATCAGCTTGAAAAGCTGATTTGCATGGCA

TGCTGCAGCTATACTTACGTACCAAAACTGCTAAAACAGAATATTTGTTCATTAACAACG

CGTGCATACAAATACTTTCAATCTTATTTGTATTTTACAAAAGAAGATCAGATCTCTTTT

TGTAAAAACTCTTTAAATTGAGGATCTGATCCTTTAAGGGCATTAGATCCTCTGGTAATT

TGTGCGATACTGACTCCGTATTTCTCTGCTATCTCTCTTTGAGTGAGCTCCCCTTCTAAA

AGAGCTCGAATGATATGATAGCGAGAAGCGACANCTNCNTNNNNNNNNNNNN

>450-11 reverse complement

CCTATAGCAGTNNNNANGNNGATTGNNCAAGCGATTAGTGGGTAACGCCAGGTTTTCCCN

TCAGNCGTTNTAAACNNGNNCAGCAATTCCTATACGACCATNTANNCCAGCGTGGTANNA

CGCAGAGTCNNTTNTNCNTTCNNTCTTCTNNTCTGGAGAAAGCCAGCACGCTTAAGAGGA

TGAANTTTNAATANCTGGAAGCNTCCGCTTGAAAANCGANTGCCAGGCATGTGCAGCTNN

CTTACGACCAAAACTGCTAAAACAGAATATTGTTCATTAACAACGCGTGCATACAAATCT

TTCAATCTTATTTGTATTTACAAAAGAAGATCAGATCTCTTTTTGTAAAAACTCTTTAAA

TTGAGGATCTGATCCTTTAAGGGCATTAGATCCTCTGGTAATTTGTGCGATACTGACTCC

GTATTTCTCTGCTATCTCTCTTTGAGTGAGCTCCCCTTCTAAAAGAGCTCGAATGATATG

ATAGCGAGAAGCGACATCTTACNNNNNNNNNNNNNNN

>550-1 reverse complement

CCCNCCTNCCGNNCCCNNNNNCCNNTNCNGNTACNCNACTGTTTGAAAGNGCNATCGGTG

CGGNNCTCTTCGCTATTNCNCCAGCNGGCGAAANNGGGATGTNNNGCAAAGGCGATTAAG

TTGGGTAACGCCAGGGTTTTCCCAGTCACGACGTTGTAAAACGACGGCCAGTGAATTCCT

AATACGACTCACTATAGGGCAAGCAGTGGTATCAACGCAGAGTACTTTTTTTTTTTTTTT

TTTTTTTTTTTTTTTTTACAGAATAATCCTGCGCAAAAAAGACGCTCTTACTTCTATAAG

AACATCTCTTAATCAAATAAAAGGCTTCTTAATTAGGCCTATCGCCAAGTAGATAAGCGC

CAGCTTTCAAAAGAAAGCCAGGCACGCTGTAAGAGGATGAATTTTTGAATATCTTGGAAG

CAATCAGCTTGAAAAGCTGATTTGCATGGCATGCTGCAGCTATACTTACGTACCAAAACT

GCTAAAACAGAATATTTGTTCATTAACAACGCGTGCATACAAATACTTTCAATCTTATTT

GTATTTTACAAAAGAAGATCAGATCTCTTTTTGTAAAAACTCTTTAAATTGAGGATCTGA

TCCTTTAAGGGCATTAGATCCTCTGGTAATTTGTGCGATACTGACTCCGTATTTCTCTGC

TATCTCTCTTTGAGTGAGCTCCCCTTCTAAAAGAGCTCGAATGATATGATAGCGAGAAGC

GACNTNTNCGTNNNNNNNNNNNC

>550-2 reverse complement

CNNNNNNNNNNGGNNNNNANGCNNCNANNNNGTNTNCNGGTCCANAGTTCGTCATCNATT

CTNNNTTNNANNNNTGNNAAANNNCGNNGATATCNNCGNAANAAGNCNGTCNNNNTNATA

GANNNNTCTTANCAANANAANNCTTNTAATTNGNCNANNNCCNNGNAGATAAGCGNAGCN

NTCAAAAGAAAGCCAGGCACGNTCTAAGAGGATGAACTTTTGAATANCTTGGAAGCAATC

NGCTTGAAAANCTGATTTGCATGGCATGCTGCAGCTNTACTTACGTACCAAAACTGCTAA

ACAGAATATTGTTCATTAACAACGCGGCATACAAATACTTTCAATCTTATTTGTATTTTA

CAAAAGAAGATCAGATCTCTTTTTGTAAAAACTCTTTAAATTGAGGATCTGATCCTTTAA

GGGCATTAGATCCTCTGGTAATTTGTGCGATACTGACTCCGTATTTCTCTGCTATCTCTC

TTTGAGTGAGCTCCCCTTCTAAAAGAGCTCGAATGATATGATAGCGAGAAGCGACATCTN

CNTCGNNNNNNNNNN

>550-4 reverse complement

CTTNCNNNGCNNNNNNCCCANNNNNNTNNGGNAANNGGCGGATCCNGTGGCTGTGCCCNN

TTCCGNCTANNNANNNCCAGGNTGGGGGAAAGGGGGGAATGTGCTGGCAAGGNCGATTAA

GNNGNGGTAACCNCCAGGGTTTTTCCCAGTCAANNACGTTGTAAAACGACNGCCAGTGAA

TTCCTAATACGACTCACTATAGGGCAAGCAGTGGTATCAACGCAGAGTACNTTTTTTTTT

TTTTTTTTTTTTTTTTTTTTTTTAAAGACGCTCTTACTTCTATAAGAACATCTCTTAATC

AAATAAAAGGCTTCNTAATTAGGCCTATCGCCAAGTAGATAAGCGCCAGCTCTCAAAAGA

AAGCCAGGCACGCTGTAAGAGGATGAATTTTTGAATANCTTGGAAGCAATCAGCTTGAAA

AGCTGATTTGCATGGCATGCTGCAGCTATACTTACGTACCAAAACTGCTAAAACAGAATA

TTTGTTCATTAACAACGCGTGCATACAAATACTTTCAATCTTATTTGTATTTCACAAAAG

AAGATCAGATCTCTTTTTGTAAAAACTCTTTAAATTGAGGATCTGATCCTTTAAGGGCAT

TAGATCCTCTGGTAATTTGTGCGATACTGACTCCGTATTTCTCTGCTATCTCTCTTTGAG

TGAGCTCCCCTTCTAAAAGAGCTCGAATGATATGATAGCGAGAAGCGACATNNNCNTNNN

NNNNNNNNN

>550-6 reverse complement

CAGATTGTACCGAAGAGGGCCCCCATANGCNGGGTGAAATNCCGNCNCNNATGCGNNAGG

AGCAAAATACCGCATCAGGCCGCCATTCGCCATTCAGGCTGCGCAACTGTTGGGAAGGGC

GATNNGTGCNGGNCTCTTCGCTATTACGCCAGCTGGCGAAAGGGCGATGTGCTGCAAGGC

GATTAAGTTGGGTAACGCCAGGGTTTTCCCAGTCACGACGTTGTAAAACGACGGCCAGTG

AATTCCTAATACGACTCACTATAGGGCAAGCAGTGGTATCAACGCAGAGTACTTTTTTTT

TTTTTTTTTTTTTTTTTTTTTTCAACTGGCGACAGAATAATCCTGCGCAAAAAAAGACGC

TCTTACTTCTATAAGAACATCTCTTAATCAAATAAAAGGCTTCTTAATTAGGCCTATCGC

CAAGTAGATAAGCGCCAGCTTTCAAAAGAAAGCCAGGCACGCTGTAAGAGGATGAATTTT

TGAATATCTTGGAAGCAATCAGCTTGAAAAGCTGATTTGCATGGCATGCTGCAGCTATAC

TTACGTACCAAAACTGCTAAAACAGAATATTTGTTCATTAACAACGCGTGCATACAAATA

CTTTCAATCTTATTTGTATTTTACAAAAGAAGATCAGATCTCTTTTTGTAAAAACTCTTT

AAATTGAGGATCTGATCCTTTAAGGGCATTAGATCCTCTGGTAATTTGTGCGATACTGAC

TCCGTATTTCTCTGCTATCTCTCTTTGAGTGAGCTCCCCTTCTAAAAGAGCTCGAATGAT

ATGATAGCGAGAAGCGACATNNNCGTCNNNNNNNNNNNN

>550-10 reverse complement

CNNNNNNNNTNGGGCNNNNTTTTNGAAAGGGCGATTCGGNNCGGGGCCTTTTTCGNTNTT

NCNCCCNGCTGGCGAAAGGGGGGATGTGCTGCAAGGCGATTAAGTTGGGCAACNCCCAGG

GTTTTCCCAGTCACGCNNNTGTCAAANCNCGCCCAGTGAATTNCNNANNCGACTCCCTAT

NGGGCAAGCAGTGGTATCAACGCAGAGTACTTTTTTTTTTTTTTTCNTTTTTTTTTCTTT

ATTANTTTTTTTTTTTTTTTCNTTTTTTTTTTTTTTTTTTTAGAATAATCCTGCGCAAAA

AAGACGCTCTTACTTCTATAAGAACATCTCTTAATCAAATACAAGGCTTCTTAATTAGGC

CTATCGCCACGTAGATAAGCGCCAGCNNTCAAAAGAAAGCCAGGCACGCTGTAAGAGGAT

GAANTTTTGAATATCTTGGAAGCAATCAGCTTGAAAAGCTGATTTGCATGGCATGCTGCA

GCTATACTTACGTACCAAAACTGCTAAAACAGAATATTTGTTCATTAACAACGCGTGCAT

ACAAATACTTTCAATCTTATTTGTATTTTACAAAAGAAGATCAGATCTCTTTTTGTAAAA

ACTCTTTAAATTGAGGATCTGATCCTTTAAGGGCATTAGATCCTCTGGTAATTTGTGCGA

TACTGACTCCGTATTTCTCTGCTATCTCTCTTTGAGTGAGCTCCCCTTCTAAAAGAGCTC

GAATGATATGATAGCGAGAAGCGACATCTTACNTNNNNNNNNNNNC

>550-11 reverse complement

GGTCTTNNNNANCACATNTAGTATANTACGCTNNCNNNCNNNAATGGTAANTTCNNCAAG

GCNANNNNNGCGGTTAACGCCNGGGTTTTTCCCAATCAAGNCGTTGTNAAACGACGGCCN

GTGAATTCNTAATNCGACTCACTATGGGGCAGGCAGTGGTATCAACGCAGAGCACTTTTC

TTTTTTTTTTTCCTTTTTNTTTTNGGCAGANAAAACTGGCGACAGAATAATCCTGCGCAA

AAAAGACGCTCNTANNACNTAAGAACATCTNTTANNCNCATACAAGGCTTNCTAATTAGG

CCTNTCGCCACGTAGATCAGCGCCAGCCCTCAAAACAAAGCCCGGCACGNNGTAAGAGGA

TGAACTTTTGAATATCNTGGAAGCAATCCGCTTGAAAAGCTGATTTGCATGGCATGCTGC

AGCTATACTTACGTACCAAAACTGCTAAAACAGAATATTTGTTCATTAACAACGCGTGCA

TACAAATACTTTCAATCTTATTTGTATTTTACAAAAGAAGATCAGATCTCTTTTTGTAAA

AACTCTTTAAATTGAGGATCTGATCCTTTAAGGGCATTAGATCCTCTGGTAATTTGTGCG

ATACTGACTCCGTATTTCTCTGCTATCTCTCTTTGAGTGAGCTCCCCTTCTAAAAGAGCT

CGAATGATATGATAGCGAGAAGCGACATCTTACNTNNNNNNNNNNNNNNNN

April 4 2018

>p250-2

GNANNNNNNGCGANGTAAGATGTCGCTTCTCGCTATCATATCATTCGAGCTCTTTTAGAAGGGGAGCTCACTCAAAGAGAGATAGCAGAGAAATACGGAGTCAGTATCGCACAAATTACCAGAGGATCTAATGCCCTTAAAGGATCAGATCCTCAATTTAAAAAAAAAAAAAAAAAAAAAAAAAAAAAAAAAGTACTCTGCGTTGATACCACTGCTTGCCCTATAGTGAGTCGTATTAGGAATTCACTGGCCGTCGTTTTACAACGTCGTGACTGGGAAAACCCTGGCGTTACCCAACTTAATCGCCTTGCAGCACATCCCCCTTTCGCCAGCTGGCGTAATAGCGAAGAGGCCCGCACCGATCGCCCTTCCCAACAGTTGCGCAGCCTGAATGGCGAATGGCGCCTGATGCGGTATTTTCTCCTTACGCATCTGTGCGGTATTTCACACCGCATATGGTGCACTCTCAGTACAATCTGCTCTGATGCCGCATAGTTAAGCCAGCCCCGACACCCGCCAACACCCGCTGACGCGCCCTGACGGGCTTGTCTGCTCCCGGCATCCGCTTACAGACAAGCTGTGACCGTCTCCGGGAGCTGCATGTGTCAGAGGTTTTCACCGTCATCACCGAAACGCGCGAGACGAAAGGGCCTCGTGATACGCCTATTTTTATAGGTTAATGTCATGATAATAATGGTTTCTTAGACGTCAGGTGGCACTTTTCGGGGAAATGTGCGCGGAACCCCTATTTGTTTATTTTTCTAAATACATTCAAATATGTATCCGCTCATGAGACAATAACCCTGATAAATGCTTCAATAATATTGAAAAAGGAAGAGTATGAGTATTCAACATTTCCGTGTCGCCCTTATTCCCTTTTTTGCGGCATTTTGCCTTCCTGTTTTTGCTCACCCAGAAACGCTGGTGAAAGTAAAAGATGCTGAAGATCAGTTGGGTGCACGAGTGGGTTACATCGAACTGGATCTCAACAGCGGTAAGATCCTTGAGAGTTTTCGCCCCGAAGAACGTTTTCCAATGATGAGCACTTTTAAAGTTCTGCTATGTGGNNCGGTATTATCCCGTATTGACGCCGGGCAAGAACAACTCGGTCGCCGCNNNCCTATTCNCANAATGACTTGGTTGAATACNCCCNNGTCNNNNAAANNCATCTTACGGATGGCNGGNNGNAAANNAATNNGCAGNGNNGCCNNAACC

>p250-3

NNNNNCNNNNANGNNGATGTCGCTTCTCGCTATCATATCATTCGAGCTCTTTTAGAAGGGGAGCTCACTCAAAGAGAGATAGCAGAGAAATACGGAGTCAGTATCGCACAAATTACCAGAGGATCTAATGCCCTTAAAGGATCAGATCCTCAATCCAAAAAAAAAAAAAAAAAAAAAAAAAAAAAAGTACTCTGCGTTGATACCACTGCTTGCCCTATAGTGAGTCGTATTAGGAATTCACTGGCCGTCGTTTTACAACGTCGTGACTGGGAAAACCCTGGCGTTACCCAACTTAATCGCCTTGCAGCACATCCCCCTTTCGCCAGCTGGCGTAATAGCGAAGAGGCCCGCACCGATCGCCCTTCCCAACAGTTGCGCAGCCTGAATGGCGAATGGCGCCTGATGCGGTATTTTCTCCTTACGCATCTGTGCGGTATTTCACACCGCATATGGTGCACTCTCAGTACAATCTGCTCTGATGCCGCATAGTTAAGCCAGCCCCGACACCCGCCAACACCCGCTGACGCGCCCTGACGGGCTTGTCTGCTCCCGGCATCCGCTTACAGACAAGCTGTGACCGTCTCCGGGAGCTGCATGTGTCAGAGGTTTTCACCGTCATCACCGAAACGCGCGAGACGAAAGGGCCTCGTGATACGCCTATTTTTATAGGTTAATGTCATGATAATAATGGTTTCTTAGACGTCAGGTGGCACTTTTCGGGGAAATGTGCGCGGAACCCCTATTTGTTTATTTTTCTAAATACATTCAAATATGTATCCGCTCATGAGACAATAACCCTGANAAAGGCTTCAAGNNNNNNNANA

>p250-4

NNNNNNNNNGNNGACGTAAGATGTCGCTTCTCGCTATCATATCATTCGAGCTCTTTTAGAAGGGGAGCTCACTCAAAGAGAGATAGCAGAGAAATACGGAGTCAGTATCGCACAAATTACCAGAGGATCTAATAAAAAAAAAAAAAAAAAAAAAAAAAAAAAAAAAAAATAAAAAAAAAAAAAAAAAAAAAAAAAAAAAGTACTCTGCGTTGATACCCCTGCTTGCCCTATAGTGAGTCGTATTAGGAATTCCCTGGCCGNNGTTTTACAACGGCGGGACTGGNAAAACCCTGGCTTTNNNAANNTTAATCGCCTTGTNANCTATCCTGNTTCCNNCATCTGGNAAATTNAAAANAGGCCGGCNTTCNTGGCCTGTGCNNACCCTTGAANGN

>p250-7

NNNNNNNNNNNNNGANGTNNNATGTCGCTTCTCGCTATCATATCATTCGAGCTCTTTTAGAAGGGGAGCTCACTCAAAGAGAGATAGCAGAGAAATACGGAGTCAGTATCGCACAAATTACCAGAGGATCTAATGCCCTTAAAGGATCAGATCCTCAATCTAAAAAAAAAAAAAAAAAAAAAAAAAAAAANNACTCTGCGTTGANACCACTGCTTGCCCTATAGTGAGTCGATTAGGAATTNCTGGCCGTCGTTTTACAACGTCGTGACTGGGAAAACCCTGGCGTTACCCAACTTAATCGCCTTGCAGCACATCCCCCTTTCGCCAGCTGGCGTAATAGCGAAGAGGCCCGCACCGATCGCCCTTCCCAACAGTTGCGCAGCCTGAATGGCGAATGGCGCCTGATGCGGTATTTTCTCCTTACGCATCTGTGCGGTATTTCACACCGNNTATGGTGCACTCTCAGTACAATCTGCTCTGATGCCGCATAGTTAAGCCAGCCCCGACACCCGCCAACACNNGCTGANNNGCNNTGNNGGNTNNNNCTGATNNGGCGTNGANNANCCANCCANTAANG

>p250-8

NNNNNNNNNNNNNANGTNNGATGTCGCTTCTCGCTATCATATCATTCGAGCTCTTTTAGAAGGGGAGCTCACTCAAAGAGAGATAGCAGAGAAATACGGAGTCAGTATCGCACAAATTACCAGAGGATCTAATGCCCTTAAAGGATCAGATCCTCAATTTAAAAAAAAAAAAAAAAAGAAAAAAAAAAAAAAAAAAAAAAAAAAAAAAAGTACTCTGCGTTGATACCACTGCTTGCCCTATAGTGAGTCGTATTAGGAATTCACTGGCCGTCGTTTTACAACGTCGTGACTGGGAAAACCCTGGCGTTACCCAACTTAATCGCCTTGCAGCACATCCCCCTTTCGCCAGCTGGCGTAATAGCGAAGAGGCCCGCACCGATCGCCCTTCCCAACAGTTGCGCAGCCTGAATGGCGAATGGCGCCTGATGCGGTATTTTCTCCTTACGCATCTGTGCGGTATTTCACACCGCATATGGTGCACTCTCAGTACAATCTGCTCTGATGCCGCATAGTTAAGCCAGCCCCGACACCCGCCAACACCCGCTGACGCGCCCTGACGGGCTTGTCTGCTCCCGGCATCCGCTTACAGACAAGCTGTGACCGTCTCCGGGAGCTGCATGTGTCAGAGGTTTTCACCGTCATCACCGAAACGCGCGAGACGAAAGGGCCTCGTGATACGCCTATTTTTATAGGTTAATGTCATGATAATAATGGTTTCTTAGACGTCAGGTGGCACTTTTCGGGGAAATGTGCGCGGAACCCCTATTTGTTTATTTTTCTAAATACATTCAAATATGTATCCGCTCATGAGACAATAACCCTGATAAATGCTTCAATAATATTGAAAAAGGAAGAGTATGAGTATTCCACNTTTTCCGGGTCGCCCTTATTCCCTTTTTTGNNGCATTTTGCCTTCNNNNT

>250-14

GNNNNNNNNNNANGNNGATGTCGCTTCTCGCTATCATATCATTCGAGCTCTTTTAGAAGGGGAGCTCACTCAAAGAGAGATAGCAGAGAAATACGGAGTCAGTATCGCACAAATTACCAGAGGATCTAATGCCCTTAAAGGATCAGATCCTCAATTGAAAAAAAAAAAAAAAAAAAAAAAAAAAAAAAAAAGTACTCTGCGTTGATACCACTGCTTGCCCTATAGTGAGTCGTATTAGGAATTCACTGGCCGTCGTTTTACAACGTCGTGACTGGGAAAACCCTGGCGTTACCCAACTTAATCGCCTTGCAGCACATCCCCCTTTCGCCAGCTGGCGTAATAGCGAAGAGGCCCGCACCGATCGCCCTTCCCAACAGTTGCGCAGCCTGAATGGCGAATGGCGCCTGATGCGGTATTTTCTCCTTACGCATCTGTGCGGTATTTCACACCGCATATGGTGCACTCTCAGTACAATCTGCTCTGATGCCGCATAGTTAAGCCAGCCCCGACACCCGCCAACACCCGCTGACGCGCCCTGACGGGCTTGTCTGCTCCCGGCATCCGCTTACAGACAAGCTGTGACCGTCTCCGGGAGCTGCATGTGTCAGAGGTTTTCACCGTCATCACCGAAACGCGCGAGACGAAAGGGCCTCGTGATACGCCTATTTTTATAGGTTAATGTCATGATAATAATGGTTTCTTAGACGTCAGGTGGCACTTTTCGGGGAAATGTGCGCGGAACCCCTATTTGTTTATTTTTCTAAATACATTCAAATATGTATCCGCTCATGAGACAATAACCCTGATAAATGCTTCAATAATATTGAAAAAGGAAGAGTATGAGTATTCAACATTTCCGTGTCGCCCTTATTCCCTTTTTTGCGGCATTTTGCCTTCCTGTTTTTGCTCACCCAGAAACGCTGGTGAAAGTAAAAGATGCTGAAGATCAGTTGGGTGCACGAGTGGGTTACATCGAACTGGATCTCAACAGCGGTAAGATCCTTGANAGTTTTCGCCCCGAANAACGTTTTCCAATGATGAACACTTTTAAAGTTCTGCTATGTGGNCGCGGTATTATCCCGTATTGANNCCGGGCAANANCAACTCGGNCNCCGCNA

>p400-4

GNNNNNNNGCGACGNNNATGTCGCTTCTCGCTATCATATCATTCGAGCTCTTTTAGAAGGGGAGCTCACTCAAAGAGAGATAGCAGAGAAATACGGAGTCAGTATCGCACAAATTACCAGAGGATCTAATGCCCTTAAAGGATCAGATCCTCAATTTAAAGAGTTTTTACAAAAAGAGATCTGATCTTCTTTTGTAAAATACAAATAAGATTGAAAGTATTTGTATGCACGCGTTGTTAATGAACAAATATTCTGTTTTAGCAGTTTTGGTACGTAAGTATAGCTGCAGCATGCCATGCAAATCAGCTTTTCAAGCTAAAAAAAAAAAAAAAAAAAAAAAAAAAAAAAAGTACTCTGCGTTGATACCACTGCTTGCCCTATAGTGAGTCGTATTAGGAATTCACTGGCCGTCGTTTTACAACGTCGTGACTGGGAAAACCCTGGCGTTACCCAACTTAATCGCCTTGCAGCACATCCCCCTTTCGCCAGCTGGCGTAATAGCGAAGAGGCCCGCACCGATCGCCCTTCCCAACAGTTGCGCAGCCTGAATGGCGAATGGCGCCTGATGCGGTATTTTCTCCTTACGCATCTGTGCGGTATTTCACACCGCATATGGTGCACTCTCAGTACAATCTGCTCTGATGCCGCATAGTTAAGCCAGCCCCGACACCCGCCAACACCCGCTGACGCGCCCTGACGGGCTTGTCTGCTCCCGGCATCCGCTTACAGACAAGCTGTGACCGTCTCCGGGAGCTGCATGTGTCAGAGGTTTTCACCGTCATCACCGAAACGCGCGAGACGAAAGGGCCTCGTGATACGCCTATTTTTATAGGTTAATGTCATGATAATAATGGTTTCTTAGACGTCAGGTGGCACTTTTCGGGGAAATGTGCGCGGAACCCCTATTTGTTTATTTTTCTAAATACATTCAAATATGTATCCGCTCATGAGACAATAACCCTGATAAATGCTTCAATAATATTGAAAAAGGAAGAGTATGAGTATTCAACATTTCCGTGTCGCCCTTATTCCCTTTTTTGCGGCATTTTGCCTTCCTGTTTTNGCTCACCNNNAANCGCTGGTGAAANTAAAANATGCTGAAGATCAGTTGGGTGCNCNANNGGNTTANNTCCAANNGGATTCNAANNCNGGNAANATCCTNNNNNNTTNCC

>p400-8

NNNNNNNNNNNNNGTNNNATGTCGCTTCTCGCTATCATATCATTCGAGCTCTTTTAGAAGGGGAGCTCACTCAAAGAGAGATAGCAGAGAAATACGGAGTCAGTATCGCACAAATTACCAGAGGATCTAATGCCCTTAAAGGATCAGATCCTCAATTTAAAGAGTTTTTACAAAAAGAGATCTGATCTTCTTTTGTAAAATACAAATAAGATTGAAAGTATTTGTATGCACGCGTTGTTAATGAACAAATATTCTGTTTTAGCAGTTTTGGTACGTAAGAAAAAAAAAAAAAAAAAAAAAAAAAAAAAAAAAAAAAAAAAAAAGAAAAAAAAAAAAAAAAAAAAAAAAAAAAAAAGTACTCTGCGTTGATACCACTGCTTGCCCTATAGGGAGTCGTATTAGGAATTCACTGGCCGTCGTTTTACAACGTCGGGACTGGGAAAACCCTGGCGTTACCCAACTTAATCGCCTTGCAGCACATCCCCCTTTCGCCAGCTGGGGTAATANCGAAAAGGCCCGCACCGATCGCCCTTCCCAACAGTTGCGCACCCTGAANGGNNAAGGGNNCCTGATGCGGTATTTTCNCCTTACNCATCTGTGCGGTATTTCACACCGCATATGGGGCACTCTCANTACAATCTGCTCTGATGCCGCATAGTTAAGCCAGCCCCGACACCCGCCAACACCCGCTGACGCGCCCTGACGGGCTTGTCTGCTCCCGGCATCCGCTTACAGACAAGCTGTGACCGTCTCCGGGAGCTGCATGTGTCAGAGGTTTTCACCGTCATCACCGAAACGCGCGAGACGAAAGGGCCTCGTGATACGCCTATTTTTTATANGTTAATGTCATGATAANAANGGTTTCTTANACGTCNNNGGCACTTTTCGGGGGAAATGTGCGCGGAACCCCTATTTGTTTANTTTTTCNAAATACNTTCAAATATGTATCCGCTCATGANANNNNNNNNGANAAATGNTTCAATAATNNNNAANNNNNNAGTANGANNANTCNNNTTTNCNNNNCNCNNNNANTNCNNTTTTGCGGCATTTTNNCNNNNNNNNNCTCNCCNNANNNCNNNNNANNAANANNCTNANANNNNNNGGNNNNNNNNNNNNNNNTNNAACNGNNATTCTNNANNNNN

>p400-10

NNNNNNNGNNGANGTNNGNTGTCGCTTCTCGCTATCATATCATTCGAGCTCTTTTAGAAGGGGAGCTCACTCAAAGAGAGATAGCAGAGAAATACGGAGTCAGTATCGCACAAATTACCAGAGGATCTAATGCCCTTAAAGGATCAGATCCTCAATTTAAAGAGTTTTTACAAAAAGAGATCTGATCTTCTTTTGTAAAATACAAATAAGATTGAAAGTATTTGTATGCACGCGTTGTTAATGAACAAATATTCTGTTTTAGCAGTTTTGGTACGTAAGTATAGCTGCAGCATGCCATAAAAAAAAAAAGAAAAAAAAAAAAAAAAAAAAAAAAAAAAAAAGTACTCTGCGTTGATACCACTGCTTGCCCTATAGTGAGTCGTATTAGGAATTCACTGGCCGTCGTTTTACAACGTCGTGACTGGGAAAACCCTGGCGTTACCCAACTTAATCGCCTTGCAGCACATCCCCCTTTCGCCAGCTGGCGTAATAGCGAAGAGGCCCGCACCGATCGCCCTTCCCAACAGTTGCGCAGCCTGAATGGCGAATGGCGCCTGATGCGGTATTTTCTCCTTACGCATCTGTGCGGTATTTCACACCGCATATGGTGCACTCTCAGTACAATCTGCTCTGATGCCGCATAGTTAAGCCAGCCCCGACACCCGCCAACACCCGCTGACGCGCCCTGACGGGCTTGTCTGCTCCCGGCATCCGCTTACAGACAAGCTGTGACCGTCTCCGGGAGCTGCATGTGTCAGAGGTTTTCACCGTCATCACCGAAACGCGCGAGACGAAAGGGCCTCGTGATACGCCTATTTTTATAGGTTAATGTCATGATAATAATGGTTTCTTAGACGTCAGGTGGCACTTTTCGGGGAAATGTGCGCGGAACCCCNATNTGGTTATTTTTCCAAAAACATTCCAATATGTATCCGCTCATGAGACAATACCCCTGAAANNGCCTTCNNTAATATTGAAAAAGGGAANAGNNNNAAGAATTCAANATTTCCNGGGNNNCCCTNNNCCCCNTTTTNGGNGGNATTTGGCCTNCCNGGTTTNANNNNNNCCNAAAAANNTNGGT

>p400-11

NNNNNNNNNNNANGTAAGATGTCGCTTCTCGCTATCATATCATTCGAGCTCTTTTAGAAGGGGAGCTCACTCAAAGAGAGATAGCAGAGAAATACGGAGTCAGTATCGCACAAATTACCAGAGGATCTAATGCCCTTAAAGGATCAGATCCTCAATTTAAAGAGTTTTTACAAAAAGAGATCTGATCTTCTTTTGTAAAATACAAATAAGATTGAAAGTATTTGTATGCACGCGTTGTTAATGAACAAATATTCTGTTTTAGCAGTTTTGGTACGTAAGTATAGCTGCAGCATGTCNAAAAAAAAAAAAAAAAAAAAAAAAAAAAATCNACTCTGCGTTGATACCACTGCTTGCCCTATAGTGAGTCTTATTACGAATGCNTGGGCCGTCGTTTTACAACGTCGGGACTGGGAAAACCCTGGTTTTACCCAACTTAATCGCCTTGCACCACATCCCCCTTTCCCCAGCTGGCGTAATACCGAACAGGCCCNCACCGATCGCCCTTCCCAACAATAGCGCAGCCTGAATGGCCAATGGCGCCTGATGCGGTATTTTCTCGTTACACGTCTGTGCGGGATTTCACACCGCATATGGTGCACTCGCAGTACAATCTGCTCTGATGNCNNNNAGTTAAGCCANCCCCGANNCCCNCCAACACCCGCTGACNCGCCCTGACGGNCTTGTCTGCTCNCGGAATCCGCTTACNNACNNGNGTGTGACCGNCNCNGGNAGCTGCATGNNNCANANGTTTGNNNCNTGATCNCNGCAAACGCNCNANATNNANCGNGCCCNNGNGANAAA

>p400-13

NNNNNNNNNNNNACGTNAGATGTCGCTTCTCGCTATCATATCATTCGAGCTCTTTTAGAAGGGGAGCTCACTCAAAGAGAGATAGCAGAGAAATACGGAGTCAGTATCGCACAAATTACCAGAGGATCTAATGCCCTTAAAGGATCAGATCCTCAATTTAAAGAGTTTTTACAAAAAGAGATCTGATCTTCTTTTGTAAAATACAAATAAGATTGAAAGTATTTGTATGCACGCGTTGTTAATGAACAAATATTCTGTTTTAGCANNTTTGGGGGGTAAATATAGCTGCACCGCGAAAAAAAAAAANAAAAAAAAATTAATCTGGATTGCTCCGATGGGATTCCCAAGAGNAGCCTTAATTTGGNCTTCCCTGGAAATGGATTGACCCANAAAGGGGAAGGCTACAACGNGGAGGGGTCNCAGCTTTAGCAAATGGTGGGGCATACAAACGTTGACAATGGGGGCATTCGGCAGGGATAGNN

>p400-14

NNNNNNNNNNNNANGTNNNATGTCGCTTCTCGCTATCATATCATTCGAGCTCTTTTAGAAGGGGAGCTCACTCAAAGAGAGATAGCAGAGAAATACGGAGTCAGTATCGCACAAATTACCAGAGGATCTAATGCCCTTAAAGGATCAGATCCTCAATTTAAAGAGTTTTTACAAAAAGAGATCTGATCTTCTTTTGTAAAATACAAATAAGATTGAAAGTATTTGTATGCACGCGTTGTTAATGAACAAATATTCTGTTTTAGCAGTTTTGGTACAAAAAAAAAAAAAAAAAAAAAAAAAAAAAAAAAAAAAAAAAAGTACTCTGCGTTGATACCACTGCTTGCCCTATAGTGAGTCGTATTAGGAATTCACTGGCCGTCGTTTTACAACGTCGTGACTGGGAAAACCCTGGCGTTACCCAACTTAATCGCCTTGCAGCACATCCCCCTTTCGCCAGCTGGCGTAATAGCGAAGAGGCCCGCACCGATCGCCCTTCCCAACAGTTGCGCAGCCTGAATGGCGAATGGCGCCTGATGCGGTATTTTCTCCTTACGCATCTGTGCGGTATTTCACACCGCATATGGTGCACTCTCAGTACAATCTGCTCTGATGCCGCATAGTTAAGCCAGCCCCGACACCCGCCAACACCCGCTGACGCGCCCTGACGGGCTTGTCTGCTCCCGGCATCCGCTTACAGACAAGCTGTGACCGTCTCCGGGAGCTGCATGTGTCAGAGGTTTTCACCGTCATCACCGAAACGCGCGAGACAAAAGGGCCTCGTGATACNCCTATTTTTATAGGTTAATGTCATGANAANAATGGTTTCTTAAANGTCAGGTG

>p450-1

NNNNNNNNNNNNANGTAAGATGTCGCTTCTCGCTATCATATCATTCGAGCTCTTTTAGAAGGGGAGCTCACTCAAAGAGAGATAGCAGAGAAATACGGAGTCAGTATCGCACAAATTACCAGAGGATCTAATGCCCTTAAAGGATCAGATCCTCAATTTAAAGAGTTTTTACAAAAAGAGATCTGATCTTCTTTTGTAAAATACAAATAAGATTGAAAGTATTTGTATGCACGCGTTGTTAATGAACAAATATTCTGTTTTAGCAGTTTTGGTACGTAAGTATAGCTGCAGCATGCCATGCAAATCAGCTTTTCAAGCTGATTGCTTCCAAGATATTCAAAAATTCATCCTCTTACAGCGTGCCTGGCTAAAAAAAAAAAAAAAAAAAAAAAAAGTACTCTGCGTTGATACCACTGCTTGCCCTATAGTGAGTCGTATTAGGAATTCACTGGCCGTCGTTTTACAACGTCGTGACTGGGAAAACCCTGGCGTTACCCAACTTAATCGCCTTGCAGCACATCCCCCTTTCGCCAGCTGGCGTAATAGCGAAGAGGCCCGCACCGATCGCCCTTCCCAACAGTTGCGCAGCCTGAATGGCGAATGGCGCCTGATGCGGTATTTTCTCCTTACGCATCTGTGCGGTATTTCACACCGCATATGGTGCACTCTCAGTACAATCTGCTCTGATGCCGCATAGTTAAGCCAGCCCCGACACCCGCCAACACCCGCTGACGCGCCCTGACGGGCTTGTCTGCTCCCGGCATCCGCTTACAGACAAGCTGTGACCGTCTCCGGGAGCTGCATGTGTCAGAGGTTTTCACCGTCATCACCGAAACGCGCGAGACGAAAGGGCCTCGTGATACGCCTATTTTTATAGGTTAATGTCATGATAATAATGGTTTCTTAGACGTCAGGTGGCACTTTTCGGGGAAATGTGCGCGGAACCCCTATTTGTTTATTTTTCTAAATACATTCCAATATGTATCCGCTCATGAAACAATAACCCTGATAAATGCTTCAATAANATTGAAAANGGAANANNNNNNGNATTCAACATTTCCGGNTCGCCCTTATTCCCTTTTTTGNGNCATTNGNCCTTCCTGGTTTTNNNNNACCNAAANCNNNNNNNNAAANTAAAAANNNNCNNAAAANNNNTTNNNNNNNCCNAANNGGNNT

>p450-3

NNNNNNNNNNNACGNAGATGTCGCTTCTCGCTATCATATCATTCGAGCTCTTTTAGAAGGGGAGCTCACTCAAAGAGAGATAGCAGAGAAATACGGAGTCAGTATCGCACAAATTACCAGAGGATCTAATGCCCTTAAAGGATCAGATCCTCAATTTAAAGAGTTTTTACAAAAAGAGATCTGATCTTCTTTTGTAAAATACAAATAAGATTGAAAGTATTTGTATGCACGCGTTGTNAATGAACAAATATTCTGTTTTAGGAGNTTTGGATGACAAGNATGNNGGCCGNCTGAGTGCCCAATAACATNGANGNGNGNATGGTGACAACACNCCACCTGCCGCCCTCCCACGCCGTGGGCTGCNCTNNAGGCAGNGGGGNGACATGNNNGATTCNNCCNCTGAAANGNNCGGCCTTTTCCTGCCNGCGGCGCTTCTTCTTTCTGANNANCCCCCATGTCCCTGAANAGNAATTCNTTCTNAACAATNCCAAATAAGAAGAAAAACCAGCCNCCCGTGANNANANATATTTTATTTTATTTNCCTCTTTTATCNCNTNTCNTANTNNGTCGTGGACCCTGANNCTA

>p450-5

GNNNNNNNNNNNNNGNNGNTGTCGCTTCTCGCTATCATATCATTCGAGCTCTTTTAGAAGGGGAGCTCACTCAAAGAGAGATAGCAGAGAAATACGGAGTCAGTATCGCACAAATTACCAGAGGATCTAATGCCCTTAAAGGATCAGATCCTCAATTTAAAGAGTTTTTACAAAAAGAGATCTGATCTTCTTTTGTAAAATACAAATAAGATTGAAAGTATTTGTATGCACGCGTTGTTAATGAACAAATATTCTGTTTTAGCAGTTTTGGTACGTAAGTATAGCTGCAGCATGCCATGCAAATCAGCTTTTCAAGCTGATTGCTTCCAAGATATTCAAAAATTCATCCTCTTACAGCGTGCCTGGCTTTCTTTTGAAAGCTGGCGCTTATAAAAAAAAAAAAAAAAAAAAAAAAAAAGTACTCTGCGTTGATACCACTGCTTGCCCTATAGTGAGTCGTATTAGGAATTCACTGGCCGTCGTTTTACAACGTCGTGACTGGGAAAACCCTGGCGTTACCCAACTTAATCGCCTTGCAGCACATCCCCCTTTCGCCAGCTGGCGTAATAGNNAANNGGCCCGCACCGATCGCCCGTCNCAACAGTTGCGCANCCTGAATGGCGAATGGNNCCTGNNNCGNGANTGNNTCATTACGCANNGGGNGNNGTACTNNNGCNNGNCGATATGAGTGCANNNCTCNNANAANNAA

>p450-6

NNNNNNNNNNNNANGNNGATGTCGCTTCTCGCTATCATATCATTCGAGCTCTTTTAGAAGGGGAGCTCACTCAAAGAGAGATAGCAGAGAAATACGGAGTCAGTATCGCACAAATTACCAGAGGATCTAATGCCCTTAAAGGATCAGATCCTCAATTTAAAGAGTTTTTACAAAAAGAGATCTGATCTTCTTTTGTAAAATACAAATAAGATTGAAAGTATTTGTATGCACGCGTTGTTAATGAACAAATATTCTGTTTTAGCAGTTTTGGTACGTAAGTATAGCTGCAGCATGCCATGCAAATCAGCTTTTCAAGCTGATTGCTTCCAAGATATTCAAAAATTCATCCTCTTACAGCGTGCCTGGCTTTCTTTTGAAAGCTGGCGCTTATAAAAAAAAAAAAAAAAAAAAAAAGAAAAAAAAAAAAAAAAAAAAAAAAAAAAAAGTACTCTGCGTTGATACCACTGCTTGCCCTATAGTGAGTCGTATTAGGAATTCACTGGCCGTCGTTTTACAACGTCGTGACTGGGAAAACCCTGGCGTTACCCAACTTAATCGCCTTGCAGCACATCCCCCTTTCGCCAGCTGGCGTAATAGCGAAGAGGCCCGCACCGATCGCCCTTCCCAACAGTTGCGCAGCCTGAATGGCGAATGGCGCCTGATGCGGTATTTTCTCCTTACGCATCTGTGCGGTATTTCACACCGCATATGGTGCACTCTCAGTACAATCTGCTCTGATGCCGCATAGTTAAGCCAGCCCCGACACCCGCCAACACCCGCTGACGCGCCCTGACGGGCTTGTCTGCTCCCGGCATCCGCTTACAGACAAGCTGTGACCGTCTCCGGGAGCTGCATGTGTCAGAGGTTTTCACCGTCATCACCGAAACGCGCGAGACGAAAGGGCCTCGTGATACGCCTATTTTTATAGGTTAATGTCATGATAATAATGGTTTCTTAGACGTCAGGTGGCACTTTTCGGGGAAATGTGCGCGGAACCCCTATTTGTTTATTTTTCTAAATACATTCAAATNNGTANCCGCTCATGNNNNAATAACCCTGAAAANGGCTTCAATAATATTGAAAAAGGAANNNTTNGNNTATTCAANNTTTCCGGNTCCCCNTTATTCCCTTTTTTGNNGNNATTTTGCCTTCCTGTTTTNNNNCCCNNNAANCCNNGGGNAANNNAAAAANNNNNAANNNNNNTTGGGNNNNCCANNGGNTTNNCNNNANNNG

>p450-7

NNNNNNNNNNNNCGTNNGATGTCGCTTCTCGCTATCATATCATTCGAGCTCTTTTAGAAGGGGAGCTCACTCAAAGAGAGATAGCAGAGAAATACGGAGTCAGTATCGCACAAATTACCAGAGGATCTAATGCCCTTAAAGGATCAGATCCTCAATTTAAAGAGTTTTTACAAAAAGAGATCTGATCTTCTTTTGTAAAATACAAATAAGATTGAAAGTATTTGTATGCACGCGTTGTTAATGAACAAATATTCTGTTTTAGCAGTTTTGGTACGTAAGTATAGCTGCAGCATGCCATGCAAATCAGCTTTTCAAGCTGATTGCTTCCAAGATATTCAAAAATTCATCCTCTTACAGCGTGCCTGGCTTTCTTTTGAAAGCTGGAAAAAAAAAAAAAAAAAAAAAAAAAAAAGTACTCTGCGTTGATACCACTGCTTGCCCTATAGTGAGTCGTATTAGGAATTCACTGGCCGTCGTTTTACAACGTCGTGACTGGGAAAACCCTGGCGTTACCCAACTTAATCGCCTTGCAGCACATCCCCCTTTCGCCAGCTGGCGTAATAGCGAAGAGGCCCGCACCGATCGCCCTTCCCAACAGTTGCGCAGCCTGAATGGCGAATGGCGCCTGATGCGGTATTTTCTCCTTACGCATCTGTGCGGTATTTCACACCGCATATGGTGCACTCTCAGTACAATCTGCTCTGATGCCGCATAGTTAAGCCAGCCCCGACACCCGCCAACACCCGCTGACGCGCCCTGACGGGCTTGTCTGCTCCCGGCATCCGCTTACAGACAAGCTGTGACCGTCTCCGGGAGCTGCATGTGTCAGAGGTTTTCACCGTCATCACCGAAACGCGCGAGACGAAAGGGCCTCGTGATACGCCTATTTTTATAGGTTAATGTCATGATAATAATGGTTTCTTAGACGTCAGGTGGCACTTTTCGGGGAAATGTGCGCGGAACCCCTATTTGTTNATTTTTCNAAATACATTCAAATANGTATCCGCTCATGAGANANTAACCCTGAAAAATGCTTCATNAANNTTGNAAAANGGANNNGNNGNNGTNTTCNANNTTNCCGNNNCCCCNTTATTCCCTTTTTTGNGNNATTTTGCCTTCCNGNTTNNGNNNNCCCNNAAACCNTGGGGAANGNNAAAAANNNNNNAANNNNNNNTTGGGNGGCNANNNGGGNTTNNNNCNAAATGGATTCCNNNNCNGAAAANTNNNNGNNNNTTTNNCCCNNANANNNNT

>p550-2

NNNNNNCNNNNACGTNNNNTGTCGCTTCTCGCTATCATATCATTCGAGCTCTTTTAGAAGGGGAGCTCACTCAAAGAGAGATAGCAGAGAAATACGGAGTCAGTATCGCACAAATTACCAGAGGATCTAATGCCCTTAAAGGATCAGATCCTCAATTTAAAGAGTTTTTACAAAAAGAGATCTGATCTTCTTTTGTAAAATACAAATAAGATTGAAAGTATTTGTATGCACGCGTTGTTAATGAACAAATATTCTGTTTTAGCAGTTTTGGTACGTAAGTATAGCTGCAGCATGCCATGCAAATCAGCTTTTCAAGCTGATTGCTTCCAAGATATTCAAAAATTCATCCTCTTACAGCGTGCCTGGCTTTCTTTTGAAAGCTGGCGCTTATCTACTTGGCGATAGGCCTAATTAAGAAGCCTTTTATTTGATTAAGAGATGTTCTTATAGAAGTAAGAGCGTCTTTTTTGCGCAAAAAAAAAAAAAAAAAAAAAAAAAAAAAAGTACTCTGCGTTGATACCACTGCTTGCCCTATAGTGAGTCGTATTAGGAATTCACTGGCCGTCGTTTTACAACGTCGTGACTGGGAAAACCCTGGCGTTACCCAACTTAATCGCCTTGCAGCACATCCCCCTTTCGCCAGCTGGCGTAATAGCGAAGAGGCCCGCACCGATCGCCCTTCCCAACAGTTGCGCAGCCTGAATGGCGAANGGCGCCTGATGCGGNATTATCTCCTTACGCATCNNTGCGGAAATTTCACACCNTNNTATGG

>p550-3

NNNNNNNNNNNNCGNNNATGTCGCTTCTCGCTATCATATCATTCGAGCTCTTTTAGAAGGGGAGCTCACTCAAAGAGAGATAGCAGAGAAATACGGAGTCAGTATCGCACAAATTACCAGAGGATCTAATGCCCTTAAAGGATCAGATCCTCAATTTAAAGAGTTTTTACAAAAAGAGATCTGATCTTCTTTTGTAAAATACAAATAAGATTGAAAGTATTTGTATGCACGCGTTGTTAATGAACAAATATTCTGTTTTAGCAGTTTTGGTACGTAAGTATAGCTGCAGCATGCCATGCAAATCAGCTTTTCAAGCTGATTGCTTCCAAGATATTCAAAAATTCATCCTCTTACAGCGTGCCTGGCTTTCTTTTGAAAGCTGGCGCTTATCTACTTGGCGATAGGCCTAATTAAGAAGCCTTTTATTTGATTAAGAGATGTTCTTATAGAAGTAAGAGCGTCTTTTTTGCGCAGGATTATTCCGAAAAAAAAAAAAAAAAAAAAAAAAAAAAAAGTACTCTGCGTTGATACCACTGCTTGCCCTATAGTGAGTCGTATTAGGAATTCACTGGCCGTCGTTTTACAACGTCGTGACTGGGAAAACCCTGGCGTTACCCAACTTAATCGCCTTGCAGCACATCCCCCTTTCGCCAGCTGGCGTAATAGCGAAGAGGCCCGCACCGATCGCCCTTCCCAACAGTTGCGCAGCCTGAATGGCGAATGGCGCCTGATGCGGTATTTTCTCCTTACGCATCTGTGCGGTATTTCACACCGCATATGGTGCACTCTCAGTACAATCTGCTCTGATGCCGCATAGTTAAGCCAGCCCCGACACCCGCCAACACCCGCTGACGCGCCCTGACGGGCTTGTCTGCTCCCGGCATCCGCTTACAGACAAGCTGTGACCGTCTCCGGGAGCTGCATGTGTCAGAGGTTTTCACCGTCATCACCGAAACGCGCGAGACGAAAGGGCCTCGTGATACGCCTATTTTTATAGGTTAATGTCATGATAATAATGGTTTCTTAGACGTCAGGNGGCANTTTTCGGGGAAATGNNNNNGGAACCCCTATTTGTTTATTTTTNNAANTACATTCAANTNNNTATCCGNNCNNGANNAANTAACCCTGNNNAANGCTTCNNNNNNNTTGAAAANGGANNNGTTNGNNNATTNNNNNTTTNCCGGNTCCCCNTTNTTCCCTTTTTTGNNGGNNTTTNNCCNNCNNNTTTNNNNNNCCC

>p550-7

NNNNNNGNNGACGTAAGATGTCGCTTCTCGCTATCATATCATTCGAGCTCTTTTAGAAGGGGAGCTCACTCAAAGAGAGATAGCAGAGAAATACGGAGTCAGTATCGCACAAATTACCAGAGGATCTAATGCCCTTAAAGGATCAGATCCTCAATTTAAAGAGTTTTTACAAAAAGAGATCTGATCTTCTTTTGTAAAATACAAATAAGATTGAAAGTATTTGTATGCACGCGTTGTTAATGAACAAATATTCTGTTTTAGCAGTTTTGGTACGTAAGTATAGCTGCAGCATGCCATGCAAATCAGCTTTTCAAGCTGATTGCTTCCAAGATATTCAAAAATTCATCCTCTTACAGCGTGCCTGGCTTTCTTTAAAAAAAAAAAAAAAAAAAAAAAAAAAAAAAAAAAAAAAAAAAAAAAAAAAAAAAAAAAAAAAAAAAAAAAAAAAAAAAAAAAAAAAAAAAAAAAAAAAAAAAAAAAAAAAAAAAAAAAAAANCCNGGGTTNANNNCCCNGGTNNNCCCAAAAGGNNNTCANNTAAGAAATTNNGGGNCCGNCTTTTTNNANCNNNGNNNCGGGAAAAACCCGGGNNTTCCCCAATNNANCCCNTTTNNACNNNNCCCCCNTTTCCNAGNGGGGNNAAAAACCAAAAAGGCCCCCCCNGATCCCCCTCNNNAACGTNGGCNNNCCCGGAAGGGCAAAGGGCCCCNNNNNCGGAATTTTCCCCTNNNNCNNGGNNGGNAATTTNNNCCNNNNANNGGGGGGCACNCCNNAAANNAANCGGCTNGNAGGCCCNNNNTTNAAACCCACCCCCGACCCNNGCCAAANCCNGGNNNAACCCCCNNNNNNNGGNTTNTTTGNTNCCGGAATTCCCTTAAAAAAAAAACNNGGNNNNNNNNNCCNGGGANNNNNNNGNNNCCAAAGNTTTTCCCCNNNNNNNCCCCGAAAANNNNNNNAAAAAAANNGGGCCCCCCGGAANNCCCCANTTTTTTAANGGGNAANGNNCNNGGGAAAAAANNGGNTTTTTTAAAGNNNTTNNGGGGGNNNNCTT

>p550-8

NNNNNNNNGNNNANGTNNGATGTCGCTTCTCGCTATCATATCATTCGAGCTCTTTTAGAAGGGGAGCTCACTCAAAGAGAGATAGCAGAGAAATACGGAGTCAGTATCGCACAAATTACCAGAGGATCTAATGCCCTTAAAGGATCAGATCCTCAATTTAAAGAGTTTTTACAAAAAGAGATCTGATCTTCTTTTGTAAAATACAAATAAGATTGAAAGTATTTGTATGCACGCGTTGTTAATGAACAAATATTCTGTTTTAGCAGTTTTGGTACGTAAGTATAGCTGCAGCATGCCATGCAAATCAGCTTTTCAAGCTGATTGCTTCCAAGATATTCAAAAATTCATCCTCTTACAGCGTGCCTGGCTTTCTTTTGAAAGCTGGCGCTTATCTACTTGGCGATAGGCCTAATTAAGAAGCCTTTTATTTGATTAAGAGATGTTCTTATAGAAGTAAGAGCGTCTTTTTTGCGCAGGAAAAAAAAAAAAAAAAAAAAAAAAAAAGTACTCTGCGTTGATACCACTGCTTGCCCTATAGTGAGTCGTATTAGGAATTCACTGGCCGTCGTTTTACAACGTCGTGACTGGGAAAACCCTGGCGTTACCCAACTTAATCGCCTTGCAGCACATCCCCCTTTCGCCAGCTGGCGTAATAGCGAAGAGGCCCGCACCGATCGCCCTTCCCAACAGTTGCGCAGCCTGAATGGCGAATGGCGCCTGATGCGGTATTTTCTCCTTACGCATCTGTGCGGTATTTCACACCGCATATGGTGCACTCTCAGTACAATCTGCTCTGATGCCGCATAGTTAAGCCAGCCCCGACACCCGCCAACACCCGCTGACGCGCCCTGACGGGCTTGTCTGCTCCCGGCATCCGCTTACAGACAAGCTGTGACCGTCTCCGGGAGCTGCATGTGTCAGAGGTTTTCACCGTCATCACCGAAACGCGCGAGACGAAAGGGCCTCGTGATACGCCTATTTTTATAGGTTAATGTCATGATAATAANGGTTTCTTANACGTCAGGNGGCACTTTTCGGGGAAATNNGGCCGGAACCCCTATTTGTTTATTTTNNNAAATANATTCAAANNNGTATCCNCTNNNGNNNNNATAACCCNGNANAAAGGCTTCAATANAATTGAAAAANGGAANNNNANNNNNNTTCCNNNTTTCCGNNCCCNNTNNNTTCCT

>p550-9

NNNNNNNNNNNANGTNNNATGTCGCTTCTCGCTATCATATCATTCGAGCTCTTTTAGAAGGGGAGCTCACTCAAAGAGAGATAGCAGAGAAATACGGAGTCAGTATCGCACAAATTACCAGAGGATCTAATGCCCTTAAAGGATCAGATCCTCAATTTAAAGAGTTTTTACAAAAAGAGATCTGATCTTCTTTTGTAAAATACAAATAAGATTGAAAGTATTTGTATGCACGCGTTGTTAATGAACAAATATTCTGTTTTAGCAGTTTTGGTACGTAAGTATAGCTGCAGCATGCCATGCAAATCAGCTTTTCAAGCTGATTGCTTCCAAGATATTCAAAAATTCATCCTCTTACAGCGTGCCTGGCTTTCTTTTGAAAGCTGGCGCTTATCTACTTGGCGATAGGCCTAATTAAGAAGCCTTTTATTTGATTAAGAGATGTTCTTATAGAAGTAAGAGCGTCTTTTTTGCGCAGGATTATTCTAAAAAAAAAAAAAAAAAAAAAAAAAAAAAGTACTCTGCGTTGATACCACTGCTTGCCCTATAGTGAGTCGTATTAGGAATTCACTGGCCGTCGTTTTACAACGTCGTGACTGGGAAAACCCTGGCGTTACCCAACTTAATCGCCTTGCAGCACATCCCCCTTTCGCCAGCTGGGGGAANAGCGAAGAGGCCCGCACCGATCGCCCCTTCCCAACAGTTGNNNNNNNNNNA

>p550-13

NNNNNNNNGNNNANGTAAGATGTCGCTTCTCGCTATCATATCATTCGAGCTCTTTTAGAAGGGGAGCTCACTCAAAGAGAGATAGCAGAGAAATACGGAGTCAGTATCGCACAAATTACCAGAGGATCTAATGCCCTTAAAGGATCAGATCCTCAATTTAAAGAGTTTTTACAAAAAGAGATCTGATCTTCTTTTGTAAAATACAAATAAGATTGAAAGTATTTGTATGCACGCGTTGTTAATGAACAAATATTCTGTTTTAGCAGTTTTGGTACGTAAGTATAGCTGCAGCATGCCATGCAAATCAGCTTTTCAAGCTGATTGCTTCCAAGATATTCAAAAATTCATCCTCTTACAGCGTGCCTGGCTTTCTTTTGAAAGCTGGCGCTTATCTACTTGGCGATAGGCCTAATTAAGAAGCCTTTTATTTGATTAAGAGATGTTCTTATAGAAGTAAGAGCGTCTTTTTTGCGCAGGATTATTCTGTCGCCAGTTTTTTCTAAAAAAAAAAAANNNNAAAAAAAANNGTACTCTGCGTTGATACCACTGCTTGCCCTATAGTGAGTCNNATTAGGAATTCACTGGCCGTCGTTNAATNCNGTCGTGACTGGGAAAATCNTGGNNTTACACAACTTAATCNCNTTGCAGCACATCCCCCTNNNNNCAGCTGGCGCAATANNNAAAAGGCCCGGACCGATCGCGCTTTCCCAACAGTNNNCNNNGCNNGGACGGANNAACGNCC

18 April 2018

>250-2

GNNNNNNNNNCGANGTNNNATGTCGCTTCTCGCTATCATATCATTCGAGCTCTTTTAGAAGGGGAGCTCACTCAAAGAGAGATAGCAGAGAAATACGGAGTCAGTATCGCACAAATTACCAGAGGATCTAATGCCCTTAAAGGATCAGATCCTCAATTTAAAAAAAAAAAAAAAAAAAAAAAAAAAAGTACTCTGCGTTGATACCACTGCTTGCCCTATAGTGAGTCGTATTAGGAATTCACTGGCCGTCGTTTTACAACGTCGTGACTGGGAAAACCCTGGCGTTACCCAACTTAATCGCCTTGCAGCACATCCCCCTTTCGCCAGCTGGCGTAATAGCGAAGAGGCCCGCACCGATCGCCCTTCCCAACAGTTGCGCAGCCTGAATGGCGAATGGCGCCTGATGCGGTATTTTCTCCTTACGCATCTGTGCGGTATTTCACACCGCATATGGTGCACTCTCAGTACAATCTGCTCTGATGCCGCATAGTTAAGCCAGCCCCGACACCCGCCAACACCCGCTGACGCGCCCTGACGGGCTTGTCTGCTCCCGGCATCCGCTTACAGACAAGCTGTGACCGTCTCCGGGAGCTGCATGTGTCAGAGGTTTTCACCGTCATCACCGAAACGCGCGAGACGAAAGGGCCTCGTGATACGCCTATTTTTATAGGTTAATGTCATGATAATAATGGTTTCTTAGACGTCAGGTGGCACTTTTCGGGGAAATGTGCGCGGAACCCCTATTTGTTTATTTTTCTAAATACATTCAAATATGTATCCGCTCATGAGACAATAACCCTGATAAATGCTTCAATAATATTGAAAAAGGAAGAGTATGAGTATTCAACATTTCCGTGTCGCCCTTATTCCCTTTTTTGCGGCATTTTGCCTTCCTGTTTTTGCTCACCCAGAAACGCTGGTGAAAGTAAAAGATGCTGAAGATCAGTTGGGTGCACGAGTGGGTTACATCGAACTGGATCTCAACAGCGGTAAGATCCTTGAAANTTTTCGCCCCGAANAACGTTTTCCAATGATGAACACTTTTAAAGTTCTGCTATNNGNNGCGGNNTTATCCCGTNTTGNNNCCGGGCAAAANCAACTCGGTCNCCNNNANCCNNTTCNNAAANNNANTTGNTNNNNNNNNNNNNNNCCNNNNAANNNNNNTTACGGNNGGNNNNNNNNAANNAANTNNNNNNNNNNCNNNANCNNNNNNNNAANCC

>250-3

NNNNNNNNNNNNACGTAAGNTGTCGCTTCTCGCTATCATATCATTCGAGCTCTTTTAGAAGGGGAGCTCACTCAAAGAGAGATAGCAGAGAAATACGGAGTCAGTATCGCACAAATTACCAGAGGATCTAATGCCCTTAAAGGATCAGATCCTCAATCAAAAAAAAAAAAAAAAAAAAAAAAAAAAAAAAGTACTCTGCGTTGATACCACTGCTTGCCCTATAGTGAGTCGTATTAGGAATTCACTGGCCGTCGTTTTACAACGTCGTGACTGGGAAAACCCTGGCGTTACCCAACTTAATCGCCTTGCAGCACATCCCCCTTTCGCCAGCTGGCGTAATAGCGAAGAGGCCCGCACCGATCGCCCTTCCCAACAGTTGCGCAGCCTGAATGGCGAATGGCGCCTGATGCGGTATTTTCTCCTTACGCATCTGTGCGGTATTTCACACCGCATATGGTGCACTCTCAGTACAATCTGCTCTGATGCCGCATAGTTAAGCCAGCCCCGACACCCGCCAACACCCGCTGACGCGCCCTGACGGGCTTGTCTGCTCCCGGCATCCGCTTACAGACAAGCTGTGACCGTCTCCGGGAGCTGCATGTGTCAGAGGTTTTCACCGTCATCACCGAAACGCGCGAGACGAAAGGGCCTCGTGATACGCCTATTTTTATAGGTTAATGTCATGATAATAATGGTTTCTTAGACGTCAGGTGGCACTTTTCGGGGAAATGTGCGCGGAACCCCTATTTGTTTATTTTTCTAAATACATTCAAATATGTATCCGCTCATGAGACAATAACCCTGATAAATGCTTCAATAATATTGAAAAAGGAAGAGTATGAGTATTCAACATTTCCGTGTCGCCCTTATTCCCTTTTTTGCGGCATTTTGCCTTCCTGTTTTTGCTCACCCAGAAACGCTGGTGAAAGTAAAAGATGCTGAAGATCAGTTGGGTGCACGAGTGGGTTACATCGAACTGGATCTCAACAGCGGTAAGATCCTTGAAAGTTTTCNCCCCGAANAACGTTTTCCANNNNTGANCNNTTTTAAAGTTNGNCANNGGNNCCGGNNTNNNCCCGNNTTGACCCNGGGCAAAANNANNNGGNCNNNNNNNACCTATTNCCAAANGGNNTGGTNNNAANNNCCCNNTCCNNAAAANCCNTNNNGGAGGNNNNNNNNAAAAAAANNNCNGGGGNCNNAANCCGNNNGNAANCNTGGNNNNNNNNNNNNNNAANNNNNGNNNNNA

>250-7

NNNNNNNNCGNNGTNNNNATGTCGCTTCTCGCTATCATATCATTCGAGCTCTTTTAGAAGGGGAGCTCACTCAAAGAGAGATAGCAGAGAAATACGGAGTCAGTATCGCACAAATTACCAGAGGATCTAATGCCCTTAAAGGATCAGATCCTCAATTTAAAGAGTTTTTATAAAAAAAAAAAAAAAAAAAAAAAAAAAAAGTACTCTGCGTTGATACCACTGCTTGCCCTATAGTGAGTCGTATTAGGAATTCACTGGCCGTCGTTTTACAACGTCGTGACTGGGAAAACCCTGGCGTTACCCAACTTAATCGCCTTGCAGCACATCCCCCTTTCGCCAGCTGGCGTAATAGCGAAGAGGCCCGCACCGATCGCCCTTCCCAACAGTTGCGCAGCCTGAATGGCGAATGGCGCCTGATGCGGTATTTTCTCCTTACGCATCTGTGCGGTATTTCACACCGCATATGGTGCACTCTCAGTACAATCTGCTCTGATGCCGCATAGTTAAGCCAGCCCCGACACCCGCCAACACCCGCTGACGCGCCCTGACGGGCTTGTCTGCTCCCGGCATCCGCTTACAGACAAGCTGTGACCGTCTCCGGGAGCTGCATGTGTCAGAGGTTTTCACCGTCATCACCGAAACGCGCGAGACGAAAGGGCCTCGTGATACGCCTATTTTTATAGGTTAATGTCATGATAATAATGGTTTCTTAGACGTCAGGTGGCACTTTTCGGGGAAATGTGCGCGGAACCCCTATTTGTTTATTTTTCTAAATACATTCAAATATGTATCCGCTCATGAGACAATAACCCTGATAAATGCTTCAATAATATTGAAAAAGGAAGAGTATGAGTATTCAACATTTCCGTGTCGCCCTTATTCCCTTTTTTGCGGCATTTTGCCTTCCTGTTTTTGCTCACCCAGAAACGCTGGTGAAAGTAAAAGATGCTGAAGATCAGTTGGGTGCACGAGTGGGTTACATCGAACTGGATCTCAACAGCGGTAAGATCCTTGAAAGTTTTCNCCCCAAAAACGTTTTCAATGATGANNANTTNAAAGTTCTGCTANNNGGCNGGNNTTNNCCCGTATTGACCCNGGGCAANANNANNNCGGTCCCNNNAANCNNNTNNNCANNNNNNTNGGTTGNNNNCCCCNANNCNNNAAAANNNNTNNCGGAGGNNNNANNTAAAAAANTTNGNNNGNNNCCNAACCTGNNNNNAAACNTGNNNCNANTTNNNNNNGANNNATNNNNNNNNAANGNNNNACCNTTT

>250-10

NNNNNNNNNNNNNNNNNNNGNTNNNNTCTANNNNANTAATCTATTTGGATTACCTAGTTCAGGGCCACGGGCTATCNCNNNNTAATACCCTCCGGGCGGGATTTTTTTGACGCTTACGCTGTAATACTAGGAAACTCCCTTTCCTCCACAAATCCCTCTTATATTATTTCTTTAAAAGTTCAGTACAACTGGTACATTTCTAAGATCAAAGTTTCCNGAGTTGGTGCTCACAACACTAATTTAAACTCAAATGATTGTAGGNTATACGTCATGATTTTTGATGANGTTTCGTGANTTTTCTGANATTTTTTNNCTTCTCTTCCACCACN

>250-12

NNNNNNNNNNNNGTNNNATGTCGCTTCTCGCTATCATATCATTCGAGCTCTTTTAGAAGGGGAGCTCACTCAAAGAGAGATAGCAGAGAAATACGGAGTCAGTATCGCACAAATTACCAGAGGATCTAATGCCCTTAAAGGATCAGATCCTCAAAAAAAAAAAATAAAAAAAAAAAAAAAAAAAAAAAAAAAAAAGTACTCTGCGTTGATACCACTGCTTGCCCTATAGTGAGTCGTATTAGGAATTCACTGGCCGTCGTTTTACAACGTCGTGACTGGGAAAACCCTGGCGTTACCCAACTTAATCGCCTTGCAGCACATCCCCCTTTCGCCAGCTGGCGTAATAGCGAAGAGGCCCGCACCGATCGCCCTTCCCAACAGTTGCGCAGCCTGAATGGCGAATGGCGCCTGATGCGGTATTTTCTCCTTACGCATCTGTGCGGTATTTCACACCGCATATGGTGCACTCTCAGTACAATCTGCTCTGATGCCGCATAGTTAAGCCAGCCCCGACACCCGCCAACACCCGCTGACGCGCCCTGACGGGCTTGTCTGCTCCCGGCATCCGCTTACAGACAAGCTGTGACCGTCTCCGGGAGCTGCATGTGTCAGAGGTTTTCACCGTCATCACCGAAACGCGCGAGACGAAAGGGCCTCGTGATACGCCTATTTTTATAGGTTAATGTCATGATAATAATGGTTTCTTAGACGTCAGGTGGCACTTTTCGGGGAAATGTGCGCGGAACCCCTATTTGTTTATTTTTCTAAATACATTCAAATATGTATCCGCTCATGAGACAATAACCCTGATAAATGCTTCAATAATATTGAAAAAGGAAGAGTATGAGTATTCAACATTTCCGTGTCGCCCTTATTCCCTTTTTTGCGGCATTTTGCCTTCCTGTTTTTGCTCACCCAGAAACGCTGGTGAAAGTAAAAGATGCTGAAGATCAGTTGGGTGCACGAGTGGGTTACATCGAACTGGATCTCAACAGCGGTAAGATCCTTGAAAGTTTTCGCCCCGAAGAACGTTTTCCAATGATGANCACTTTTAAAGTTCTGCNNNNNGGNNNNGGNNTTATCCCGTNTTGACGCCGGGCAAGANCAACTCGGTCNCCNNNNNNNNNNTTNTNNNAAGNACTTGGTTNNNNACNCCCNNNNCNNGAAANNNTTCTTACGGATGGCTGGANNTAANNANNTNNGNNNNNNNNNCNNAANNNNNNNNNAANNNTGGGGNNNANTTAT

>250-14

NNNNNNNNNNNNNGANGTNNNATGTCGCTTCTCGCTATCATATCATTCGAGCTCTTTTAGAAGGGGAGCTCACTCNNNNAGAGATAGCAGAGAAATACGGAGTCAGTATCGCACAAATTACCAGAGGATCTAATGCCCTTAAAGGATCAGATCCTCAATTTAAAGAGTTTAAAAAAAAAAAAAGAAAAAAAAAAAAAAAAAAAAAAAAAAAAGTACTCTGCGTTGATACCACTGCTTGCCCTATAGTGAGTCGTATTAGGAATTCACTGGCCGTCGTTTTACAACGTCGTGACTGGGAAAACCCTGGCGTTACCCAACTTAATCGCCTTGCAGCACATCCCCCTTTCGCCAGCTGGCGTAATAGCGAAGAGGCCCGCACCGATCGCCCTTCCCAACAGTTGCGCAGCCTGAATGGCGAATGGCGCCTGATGCGGTATTTTCTCCTTACGCATCTGTGCGGTATTTCACACCGCATATGGTGCACTCTCAGTACAATCTGCTCTGATGCCGCATAGTTAAGCCAGCCCCGACACCCGCCAACACCCGCTGACGCGCCCTGACGGGCTTGTCTGCTCCCGGCATCCGCTTACAGACAAGCTGTGACCGTCTCCGGGAGCTGCATGTGTCAGAGGTTTTCACCGTCATCACCGAAACGCGCGAGACGAAAGGGCCTCGTGATACGCCTATTTTTATAGGTTAATGTCATGATAATAATGGTTTCTTAGACGTCAGGTGGCACTTTTCGGGGAAATGTGCGCGGAACCCCTATTTGTTTATTTTTCTAAATACATTCAAATATGTATCCGCTCATGAGACAATAACCCTGATAAATGCTTCAATAATATTGAAAAAGGAAGAGTATGAGTATTCAACATTTCCGTGTCNCACTTATTCCCTTTTTTGCGGCATTTTGCCTTCCTGTTTTTGCTCACCCAGAAACGCTGGTGAAAGTAAAAGATGCTGAAGNTCAGTTGGGTGCACGANNGGGTTACATCGAACTGGATCTCAACAGCGGTAAGATCCTTGAANNNTTTNNCCCCNAAGAACGTTTTCCATGATGAGNNTTTNAANTNCTGCTAGNNGCGNGGNNNNNCCNNNTTGACGCGGNCAAGNNNNNCGGTCCCNNNNNCNNTNNNCAANNNACTGGNTGNNNNCCNNNNTCCNNAANNNNCTNACGGNGNCTNNNNNNAANAANTNNGNNNT

>400-1

NNNNNNNGNNNANGTNNNATGTCGCTTCTCGCTATCATATCATTCGAGCTCTTTTAGAAGGGGAGCTCACTCAAAGAGAGATAGCAGAGAAATACGGAGTCAGTATCGCACAAATTACCAGAGGATCTAATGCCCTTAAAGGATCAGATCCTCAATTTAAAGAGTTTTTACAAAAAGAGATCTGATCTTCTTTTGTAAAATACAAATAAGATTGAAAGTATTTGTATGCACGCGTTGTTAATGAACAAATATTCTGTTTTAGCAGTTTTGGTACGTAAGTATAGCTGCAGCATGCCATGCAAATCAGCTTTTCAAGCTGATTGCTTCCAAGATAAAAAAAAAAAAAAAAAAAAAAAAAAAAAGTACTCTGCGTTGATACCACTGCTTGCCCTATAGTGAGTCGTATTAGGAATTCACTGGCCGTCGTTTTACAACGTCGTGACTGGGAAAACCCTGGCGTTACCCAACTTAATCGCCTTGCAGCACATCCCCCTTTCGCCAGCTGGCGTAATAGCGAAGAGGCCCGCACCGATCGCCCTTCCCAACAGTTGCGCAGCCTGAATGGCGAATGGCGCCTGATGCGGTATTTTCTCCTTACGCATCTGTGCGGTATTTCACACCGCATATGGTGCACTCTCAGTACAATCTGCTCTGATGCCGCATAGTTAAGCCAGCCCCGACACCCGCCAACACCCGCTGACGCGCCCTGACGGGCTTGTCTGCTCCCGGCATCCGCTTACAGACAAGCTGTGACCGTCTCCGGGAGCTGCATGTGTCAGAGGTTTTCACCGTCATCACCGAAACGCGCGAGACGAAAGGGCCTCGTGATACGCCTATTTTTATAGGTTAATGTCATGATAATAATGGTTTCTTAGACGTCAGGTGGCACTTTTCGGGGAAATGTGCGCGGAACCCCTATTTGTTTATTTTTCTAAATACATTCAAATATGTATCCGCTCATGANACAATAACCCTGANAAANGCTTCANTAANNTTGAAAAAGGGANNNTNTGAGTATTCAACATTTCCGGGTCCCCCTTATTCCCTTTTTNGNGNCATTTTGCCTTCCNGNTTTNGCTNNCCCAAAANCCNNGGNNAAANGTAAAAANNNNNAAAANNNNTTNGGGNGGCCNAAGGGGNTTANCNNNANNNGNNTTCNNNNNNNGGNAAAANCCNNNNNNNTTNNCNCCCNGANAANNTTTNCCAAGNNNNGNCNTTTTAAANTTNNNNNNNGGNNNNNNNA

>400-3

NNNNNNNNNNNNGTAAAGATGTCGCTTCTCACTATCATATCATTCGAGCTCTTTTAGAAGGGGAGCTCACTCAAAGAGAGATAGCAGAGAAATACGGAGTCAGTATCGCACAAATTACCAGAGGATCTAATGCCCTTAAAGGATCAGATCCTCAATTTAAAGAGTTTTTACAAAAAGAGATCTGATCTTCTTTTGTAAAATACAAATAAGATTGAAAGTATTTGTATGCACGCGTTGTTAATGAACAAATATTCTGTTTTAGCAGTTTTGGTACGTAAGTATAGCTGCAGCATGCCATGCAAATCAGCTTAAGAAAAAAAAAAAAAAAAAAAAAAAAAAAAAAGTACTCTGCGTTGATACCACTGCTTGCCCTATAGTGAGTCGTATTAGGAATTCACTGGCCGTCGTTTTACAACGTCGTGACTGGGAAAACCCTGGCGTTACCCAACTTAATCGCCTTGCAGCACATCCCCCTTTCGCCAGCTGGCGTAATAGCGAAGAGGCCCGCACCGATCGCCCTTCCCAACAGTTGCGCAGCCTGAATGGCGAATGGCGCCTGATGCGGTATTTTCTCCTTACGCATCTGTGCGGTATTTCACACCGCATATGGTGCACTCTCAGTACAATCTGCTCTGATGCCGCATAGTTAAGCCAGCCCCGACACCCGCCAACACCCGCTGACGCGCCCTGACGGGCTTGTCTGCTCCCGGCATCCGCTTACAGACAAGCTGTGACCGTCTCCGGGAGCTGCATGTGTCAGAGGTTTTCACCGTCATCACCGAAACGCGCGAGACGAAAGGGCCTCGTGATACGCCTATTTTTATAGGTTAATGTCATGATAATAATGGTTTCTTAGACGTCAGGTGGCACTTTTCGGGGAAATGTGCGCGGAACCCCTATTTGTTTATTTTTCTAAATACATTCAAATATGTATCCGCTCATGAGACAATAACCCTGATAAATGCTTCAATAATATTGAAAAAGGAAGAGTATGAGTATTCAACATTTCCGGGTCCCCCTTATTCCCTTTTTTGCGGNATTTTGCCTTCCGNTTTTNGCCCCCNAAAANCCNTGGNGAAANNAAAAANNCTGAAANNNNNTGGGGNCCNAANNGGGTTANNNAANTGGNATTCNNNNNGGGNAAANTNNTGGAANTTTNCCCCCNAAAANNNTTTCCNNNNNNNACNNTTTAA

>400-6

NNNNNNNGNNNANGTNNNATGTCGCTTCTCGCTATCATATCATTCGAGCTCTTTTAGAAGGGGAGCTCACTCAAAGAGAGATAGCAGAGAAATACGGAGTCAGTATCGCACAAATTACCAGAGGATCTAATGCCCTTAAAGGATCAGATCCTCAATTTAAAGAGTTTTTACAAAAAGAGATCTGATCTTCTTTTGTAAAATACAAATAAGATTGAAAGTATTTGTATGCACGCGTTGTTAATGAACAAATATTCTGTTTTAGCAGTTTTGGTACGTAAGTATAGCTGCAGCATGCCATGCAAATCAGCTTTTCAAGCCAAAAAAAAAAAAAAAAAAAAAAAAAAAAGTACTCTGCGTTGATACCACTGCTTGCCCTATAGTGAGTCGTATTAGGAATTCACTGGCCGTCGTTTTACAACGTCGTGACTGGGAAAACCCTGGCGTTACCCAACTTAATCGCCTTGCAGCACATCCCCCTTTCGCCAGCTGGCGTAATAGCGAAGAGGCCCGCACCGATCGCCCTTCCCAACAGTTGCGCAGCCTGAATGGCGAATGGCGCCTGATGCGGTATTTTCTCCTTACGCATCTGTGCGGTATTTCACACCGCATATGGTGCACTCTCAGTACAATCTGCTCTGATGCCGCATAGTTAAGCCAGCCCCGACACCCGCCAACACCCGCTGACGCGCCCTGACGGGCTTGTCTGCTCCCGGCATCCGCTTACAGACAAGCTGTGACCGTCTCCGGGAGCTGCATGTGTCAGAGGTTTTCACCGTCATCACCGAAACGCGCGAGACGAAAGGGCCTCGTGATACGCCTATTTTTATAGGTTAATGTCATGATAATAATGGTTTCTTAGACGTCAGGTGGCACTTTTCGGGGAAATGTGCGCGGAACCCCTATTTGTTTATTTTTCTAAATACATTCAAATATGTATCCGCTCATGANACAATAACCCTGATAAATGCTTCCATAATATTGGAAAANGGAAGAGTATGAGTATTCAANATTTCCGNGTCGCCCTTATTCCCTTTTTTGCGGCATTTTGCCTTCCGGTTTTNGCTNNCCNAAAAACGCTGGTGAAANNAAAAANNGCTGAAANTNAATTGGNNGCCNAANNGGNTTNNNNNNAANNGGNTNCNAANNNNGGNAANNCCTTGGANNNTTNNNCCCNGAAAACNTTTTCCAAGNAGAANNNTTTNAANGTNNNNNNANNGGNNNNGGNNNNNNNCCGNNTNGGCCCNGGNAAANNNNNNNNNNNNC

>400-7

NNNNNNNNNNCGNNGTNNNATGTCGCTTCTCGCTATCATATCATTCGAGCTCTTTTAGAAGGGGAGCTCACTCAAAGAGAGATAGCAGAGAAATACGGAGTCAGTATCGCACAAATTACCAGAGGATCTAATGCCCTTAAAGGATCAGATCCTCAATTTAAAGAGTTTTTACAAAAAGAGATCTGATNAAAAAAAAAAAAAAAAAAAAAAAAAAAAAAAAAAAAAAAAAAAAAAAAAAAAAAAAANGAAAAAAAAAAAAAAAAAAAAAAAAAAAANNNNNNNGNGTTGNNCCNGGCTTGCCCNANGGGAGNNANTTAGGAATTCNGGGCNNNGTTTTACAACGTCGNNACGGGGAAAACCCTGGCNTNCCCAACTTAATCGCCTTGCAGCACATCCCCCTTTCCCCAGNTGGGGAANNAGNGAAAAGGCCCGCACCGATCGCCCTTCCCAACAGTNGNNCACCCNNAAGGGCAAAGGGCCCCNNAGGCGGTATTTTCTCCTTACGCATCTGGGCGGNNTTTCACACCGCANNGGGGGCACTNNCAGTACAATNNNCTCNNNTGCCGCANAGTTAAGCCAGCCCCGACACCCGCCAACACCCGCNNACGNNCCCTGACGGGCTTGTNGNCTCCCGGCATCCNCTTAANAANGCTGNNACCGTCTCGGGNGCTGCNNGNTCAAANNTTTCNCCGTCATCACCAAACNNCNAANAAGGGCNCGNAACCCNTTTTATAGGTAAGTCANGAAAAANGTNNNAANTNGGGGNNTTTNGGGNANNGNNGNACCCNTTGTNNTTTNAANNNTCAANNGNTCNNNNGAACANACNGAANTGCTCANANTGAAAGANNNNGNNNTCANTTCGNCNCCTNTCCTTTGNGNTTGCNCNGTTTNNNNCAANCNNGGNAAANNGNNAANATGGNNNNNGGNNNNNNNNNNNNNGANNCTNANTTCCCNANNTTNAGNNNNTTAGTNNNGNNGNNNCGNGCCGAANNNGNCNNCNTCAANNGNNA

>400-9

NNNNNNGNNNNGTAAGNTGTCGCTTCTCGCTATCATATCATTCGAGCTCTTTTAGAAGGGGAGCTCACTCAAAGAGAGATAGCAGAGAAATACGGAGTCAGTATCGCACAAATTACCAGAGGATCTAATGCCCTTAAAGGATCAGATCCTCAATTTAAAGAGTTTTTACAAAAAGAGATCTGATCTTCTTTTGTAAAATACAAATAAGATTGAAAGTATTTGTATGCACGCGTTGTTAATGAACAAATATTCTGTTTTAGCAGTTTTGGTACGTAAGTATAGCTGCAGCATGCCATGCAAATCAGCTTTTCAAGCTGATTGCTTCCAAGATATAAAAAAAAAAAAAAAAAAAAAAAAAAAAAAGTACTCTGCGTTGATACCACTGCTTGCCCTATAGTGAGTCGTATTAGGAATTCACTGGCCGTCGTTTTACAACGTCGTGACTGGGAAAACCCTGGCGTTACCCAACTTAATCGCCTTGCAGCACATCCCCCTTTCGCCAGCTGGCGTAATAGCGAAGAGGCCCGCACCGATCGCCCTTCCCAACAGTTGCGCAGCCTGAATGGCGAATGGCGCCTGATGCGGTATTTTCTCCTTACGCATCTGTGCGGTATTTCACACCGCATATGGTGCACTCTCAGTACAATCTGCTCTGATGCCGCATAGTTAAGCCAGCCCCGACACCCGCCAACACCCGCTGACGCGCCCTGACGGGCTTGTCTGCTCCCGGCATCCGCTTACAGACAAGCTGTGACCGTCTCCGGGAGCTGCATGTGTCAGAGGTTTTCACCGTCATCACCGAAACGCGCGAGACGAAAGGGCCTCGTGATACGCCTATTTTTATAGGTTAATGTCATGATAATAATGGTTTCTTAGACGTCAGGTGGCACTTTTCGGGGAAATGTGCGCGGAACCCCTATTTGTTTATTTTTCTAAATACATTCAAATATGTATCCGCTCATGAGACAATAACCCTGATAAATGCTTCATAATATTGGAAAAGGGAANAGTATGAGTATTCAACATTTCCGTGTCNCCCTTATTCCCTTTTTTGCGGCATTTTGCCTTCCGGTTTNNNNNNCCNAAAAACGCTGGNGAANGTAAAAAANNCTGAAANNNNTTNGGNGGCCNAATGGNTTNCNNNAACTGGATTCNNNNNNNGGAANNNCNTNNAANNTTNNNCCNGAAANNNTTNCNANNNNNNNNTTTNAAGTNNNNNNNGGGNNGGNNNNNCCNGNNNNNNCNGGNNGANNNNNNGNNNNNNNNANNNTTNC

>400-14

NNNNNNNNNNNNNNGTAAGNTGTCGCTTCTCGCTATCATATCATTCGAGCTCTTTTAGAAGGGGAGCTCACTCAAAGAGAGATAGCAGAGAAATACGGAGTCAGTATCGCACAAATTACCAGAGGATCTAATGCCCTTAAAGGATCAGATCCTCAATTTAAAGAGTTTTTACAAAAAGAGATCTGATCTTCTTTTGTAAAATACAAATAAGATTGAAAGTATTTGTATGCACGCGTTGTTAATGAACAAATATTCTGTTTTAGCAGTTTTGGTACGTAAGTATAGCTGCAGCATGCCAAAAAAAAAAAAAAAAAAAAAAAAAAAAAAGTACTCTGCGTTGATACCACTGCTTGCCCTATAGTGAGTCGTATTAGGAATTCACTGGCCGTCGTTTTACAACGTCGTGACTGGGAAAACCCTGGCGTTACCCAACTTAATCGCCTTGCAGCACATCCCCCTTTCGCCAGCTGGCGTAATAGCGAAGAGGCCCGCACCGATCGCCCTTCCCAACAGTTGCGCAGCCTGAATGGCGAATGGCGCCTGATGCGGTATTTTCTCCTTACGCATCTGTGCGGTATTTCACACCGCATATGGTGCACTCTCAGTACAATCTGCTCTGATGCCGCATAGTTAAGCCAGCCCCGACACCCGCCAACACCCGCTGACGCGCCCTGACGGGCTTGTCTGCTCCCGGCATCCGCTTACAGACAAGCTGTGACCGTCTCCGGGAGCTGCATGTGTCAGAGGTTTTCACCGTCATCACCGAAACGCGCGAGACGAAAGGGCCTCGTGATACGCCTATTTTTATAGGTTAATGTCATGATAATAATGGTTTCTTAGACGTCAGGTGGCACTTTTCGGGGAAATGTGCGCGGAACCCCTATTTGTTTATTTTTCTAAATACATTCAAATATGTATCCGCTCATGAGACAATAACCCTGATAAATGCTTCAATAATATTGAAAAAGGAANAGTATGAGTATTCAACATTTCCGTGTCGCCCTTATTCCCTTTTTTGCGGCATTTTGCCTTCCNGTTTTNGCTCACCNAAANCGCTGGTGAAAGTAAANNANGCTGAANATCNNTTGGNNNCCNANTGGGTTANNCNAACTGGATCNNNNNNGGNNAAANNCCTNNNNNNTTNCCCCNNAAANNGTTTTCCAAGGNNGNNNTTTNAAAGNNNGNNNNNGGGGNNGGNNNNNCC

>450-2

NNNNNNNNNNNGTAAGNTGTCGCTTCTCGCTATCATATCATTCGAGCTCTTTTAGAAGGGGAGCTCACTCAAAGAGAGATAGCAGAGAAATACGGAGTCAGTATCGCACAAATTACCAGAGGATCTAATGCCCTTAAAGGATCAGATCCTCAATTTAAAGAGTTTTTACAAAAAGAGATCTGATCTTCTTTTGTAAAATACAAATAAGATTGAAAGTATTTGTATGCACGCGTTGTTAATGAACAAATATTCTGTTTTAGCAGTTTTGGTACGTAAGTATAGCTGCAGCATGCCATGCAAATCAGCTTTTCAAGCTGATTGCTTCCAAGATATTCAAAAATTCATCCTCTTACAGCGTGCCTGGCTTTCTTTTGAAAGCTGGCGCTTATCCAAAAAAAAAAAAAAAAAAAAAAAAAAAAAAGTACTCTGCGTTGATACCACTGCTTGCCCTATAGTGAGTCGTATTAGGAATTCACTGGCCGTCGTTTTACAACGTCGTGACTGGGAAAACCCTGGCGTTACCCAACTTAATCGCCTTGCAGCACATCCCCCTTTCGCCAGCTGGCGTAATAGCGAAGAGGCCCGCACCGATCGCCCTTCCCAACAGTTGCGCAGCCTGAATGGCGAATGGCGCCTGATGCGGTATTTTCTCCTTACGCATCTGTGCGGTATTTCACACCGCATATGGTGCACTCTCAGTACAATCTGCTCTGATGCCGCATAGTTAAGCCAGCCCCGACACCCGCCAACACCCGCTGACGCGCCCTGACGGGCTTGTCTGCTCCCGGCATCCGCTTACAGACAAGCTGTGACCGTCTCCGGGAGCTGCATGTGTCAGAGGTTTTCACCGTCATCACCGAAACGCGCGAGACGAAAGGGCCTCGTGATACGCCTATTTTTATAGGTTAATGTCATGATAATAATGGTTTCTTAGACGTCAGGTGGCACTTTTCGGGGAAATGTGCGCGGAACCCCTATTTGTTTATTTTTCTAAATACATTCAAATATGTATCCGCTCATGAAACAATAACCCTGNNAAAGNCTTCAATAATATTGAAAANGGNANNNNTNGNAATATTCAACATTTCCNGNNCNCCCTTNTNCCCTTTTTTGNGGNNNTTNGNCCTTCCGGTTTTNNNNNNCCAAAAANGNNGGGGNAANNAAANAANNNCGAAAANNNNTTNGGNNGGCCNANNNGGNTTANNNNAANNTGGATTCCNNNNNGGNNANNNCNTTNNNNNNTTNCNCCCNGAANNNNTTTNCCNNNNNNNNNCCTTTNAANTNNNNNNNNG

>450-4

NNNNNNNGNNGANGNNNNATGTCGCTTCTCGCTATCATATCATTCGAGCTCTTTTAGAAGGGGAGCTCACTCAAAGAGAGATAGCAGAGAAATACGGAGTCAGTATCGCACAAATTACCAGAGGATCTAATGCCCTTAAAGGATCAGATCCTCAATTTAAAGAGTTTTTACAAAAAGAGATCTGATCTTCTTTTGTAAAATACAAATAAGATTGAAAGTATTTGTATGCACGCGTTGTTAATGAACAAATATTCTGTTTTAGCAGTTTTGGTACGTAAGTATAGCTGCAGCATGCCATGCAAATCAGCTTTTCAAGCTGATTGCTTCCAAGATATTCAAAAATTCATCCTCTTACAGCGTGCCTGGCTTTCTTTTGAAAGCTGGCGCTTAAAAGAAAAAAAAAAAAAAAAAAAAAAAAAAAAGTACTCTGCGTTGATACCACTGCTTGCCCTATAGTGAGTCGTATTAGGAATTCACTGGCCGTCGTTTTACAACGTCGTGACTGGGAAAACCCTGGCGTTACCCAACTTAATCGCCTTGCAGCACATCCCCCTTTCGCCAGCTGGCGTAATAGCGAAGAGGCCCGCACCGATCGCCCTTCCCAACAGTTGCGCAGCCTGAATGGCGAATGGCGCCTGATGCGGTATTTTCTCCTTACGCATCTGTGCGGTATTTCACACCGCATATGGTGCACTCTCAGTACAATCTGCTCTGATGCCGCATAGTTAAGCCAGCCCCGACACCCGCCAACACCCGCTGACGCGCCCTGACGGGCTTGTCTGCTCCCGGCATCCGCTTACAGACAAGCTGTGACCGTCTCCGGGAGCTGCATGTGTCAGAGGTTTTCACCGTCATCACCGAAACGCGCGAGACGAAAGGGCCTCGTGATACGCCTATTTTTATAGGTTAATGTCATGATAATAATGGTTTCTTAGACGTCAGGTGGCACTTTTCGGGGAAATGTGCGCGGAACCCCTATTTGTTTATTTTTCTAAATACATTCAAATATGTATCCGCTCATGAAANANTAACCCTGANAAATGCTTCAATAANNTTGNAAANGGGAANNGTNNGAGTATTCAANNTTTCCGGNNCCCCCTTATTCCCTTTTTTGNGNCATTTNGCCTTCCGNTTTNNNNNNCCNAAAACCNNGGGGNAANNAAAAANNNCTGAAANNNNNTTGGNNNCNNAANNGGG

>450-5

NNNNNNNNNNCNANGTNNNNATGTCGCTTCTCGCTATCATATCATTCGAGCTCTTTTAGAAGGGGAGCTCACTCAAAGAGAGATAGCAGAGAAATACGGAGTCAGTATCGCACAAATTACCAGAGGATCTAATGCCCTTAAAGGATCAGATCCTCAATTTAAAGAGTTTTTACAAAAAGAGATCTGATCTTCTTTTGTAAAATACAAATAAGATTGAAAGTATTTGTATGCACGCGTTGTTAATGAACAAATATTCTGTTTTAGCAGTTTTGGTACGTAAGTATAGCTGCAGCATGCCATGCAAATCAGCTTTTCAAGCTGATTGCTTCCAAGATAAAAAAAAAAAAAAAAAAAAAAAAAAAAAAAAAAAAAAAAAGAAAAAAAAAAAAAAAAAAAAAAAAAAAAAAGTACTCTGCGTTGATACCACTGCTTGCCCTATAGTGAGTCGTATTAGGAATTCACTGGCCGTCGTTTTACAACGTCGTGACGGGNAAAACCCTGGCGTTACCCAACTTAATCGCCTTGCAGCACATCCCCCTTTCGCCAGCTGGCGTAATAGCGAAAAGGCCCGCACCGATCGCCCTTCCCAACAGTTGCGCAGCCTGAATGGCGAAGGNCGCCTGATGCGGTATTTTCTCCTTACGCATCTGTGCGGTATTTCACACCGCATATGGGNCACTCTCAGTACAATCTGCTCTGATGCCGCATAGTTAAGCCAGCCCCGACACCCGCCAACACCCGCTGACGCGCCCTGACGGGCTTGTCTGCTCCCGGCATCCGCTTACAGACAAGCTGTGACCGTCTCCGGGAGCTGCATGTGTCANNGGTTTTCACCGTCATCACCGAAACGCGCGAGACGAAAGGGCCTCGTGATACGCCTATTTTTATAGGTTAATGTCATGATAATAAGGGTTTCTTAAACGTCAGGTGGCACTTTTCGGGGAAATGTGCGCGGAACCCCTATTTGTTTATTTTTCTAAATACATTCAAATNNGTATCCGCTCATGAAACAATAACCCNGAAAATGCTTCANNAANNTTGGNAAGGGAANNNTTNNNGNATCNANNNTTNCCGGNNCCCCNTTATTCCCTTTTNGNGGAATTGNCCTTCCGGTTTNGNNNNNCCNAAACCNGGGGNAAANNAANAANNNNNAAANCTNNGGGGNCNAANGGGGTTNNCNNNNNGNNNCCNNNNGGG

>450-9

NNNNNNNNNNGNGANGTANGATGTCGCTTCTCGCTATCATATCATTCGAGCTCTTTTAGAAGGGGAGCTCACTCAAAGAGAGATAGCAGAGAAATACGGAGTCAGTATCGCACAAATTACCAGAGGATCTAATGCCCTTAAAGGATCAGATCCTCAATTTAAAGAGTTTTTACAAAAAGAGATCTGATCTTCTTTTGTAAAATACAAATAAGATTGAAAGTATTTGTATGCACGCGTTGTTAATGAACAAATATTCTGTTTTAGCAGTTTTGGTACGTAAGTATAGCTGCAGCATGCCATGCAAATCAGCCTTTCAAGCTGATTGCTTCCAAGATATTCAAAAATTCATCCTCTTACAGCGTGCCTGGCTTTCTTTTGAAAGCTGGCGCTTATCAAAAAAAAAAAAAAAAAAAAAAAAAAAAAAAAGTACTCTGCGTTGATACCACTGCTTGCCCTATAGTGAGTCGTATTAGGAATTCACTGGCCGTCGTTTTACAACGTCGTGACTGGGAAAACCCTGGCGTTACCCAACTTAATCGCCTTGCAGCACATCCCCCTTTCGCCAGCTGGCGTAATAGCGAAGAGGCCCGCACCGATCGCCCTTCCCAACAGTTGCGCAGCCTGAATGGCGAATGGCGCCTGATGCGGTATTTTCTCCTTACGCATCTGTGCGGTATTTCACACCGCATATGGTGCACTCTCAGTACAATCTGCTCTGATGCCGCATAGTTAAGCCAGCCCCGACACCCGCCAACACCCGCTGACGCGCCCTGACGGGCTTGTCTGCTCCCGGCATCCGCTTACAGACAAGCTGTGACCGTCTCCGGGAGCTGCATGTGTCAGAGGTTTTCACCGTCATCACCGAAACGCGCGAGACGAAAGGGCCTCGTGATACGCCTATTTTTATAGGTTAATGTCATGATAATAATGGTTTCTTAGACGTCAGGTGGCACTTTTCGGGGAAATGTGCGCGGAACCCCTATTTGTTTATTTTTCTAAATACATTCAAATATGTATCCGCTCATGAAANANTAACCCTGAAAANTGCTTCAATAANNTTGAAAANGGNANNNGTTNGANTNTTCNANNTTNCCGGNTCCCCNTTATTCCCTTTTTNNNGNNNTTTNGCCTTCCGGTTTTNGNNCCCCNAAAANCNNNGGNGAANNNAAAAAANNNNNAAAANNNNTNGGGGGNCCNANNGGNTTNNNNNNAACTGGATNNNNNNNNGNNAAANTCCTNNAANNTTTCNNCCNAAANNNTTT

>450-11

NNNNNNNNNNNNGANGTNNGATGTCGCTTCTCGCTATCATATCATTCGAGCTCTTTTAGAAGGGGAGCTCACTCAAAGAGAGATAGCAGAGAAATACGGAGTCAGTATCGCACAAATTACCAGAGGATCTAATGCCCTTAAAGGATCAGATCCTCAATTTAAAGAGTTTTTACAAAAAGAGATCTGATCTTCTTTTGTAAAATACAAATAAGATTGAAAGTATTTGTATGCACGCGTTGTTAATGAACAAATATTCTGTTTTAGCAGTTTTGGTACGTAAGTATAGCTGCAGCATGCCATGCAAATCAGCTTTTCAAGCTGATTGCTTCCAAGATATTCAAAAATTCATCCTCTTACAGCGTGCCTGGCTTTCTTTTGAAAGCTGGCGCTCAAAAAAAAAAAAAAAAAAAAAAAAAAANGTACTCTGCGTTGATACCACTGCTTGCCCTATAGTGAGTCGTATTAGGAATTCACTGGCCGTCGTTTTACAACGTCGTGACTGGGAAAACCCTGGCGTTACCCAACTTAATCGCCTTGCAGCACATCCCCCTTTCGCCAGCTGGCGTAATAGCGAAGAGGCCCGCACCGATCGCCCTTCCCAACAGTTGCGCAGCCTGAATGGCGAATGGCGCCTGATGCGGTATTTTCTCCTTACGCATCTGTGCGGTATTTCACACCGCATATGGTGCACTCTCAGTACAATCTGCTCTGATGCNNNATAGTTAAGCCAGCCCCGACACCCGCCAACACCCGCTGACGCGNCCTGACGGGCTTGTCTGCTCCCGGCATCCGCTTACAGACAAGCTGTGACCGTCTCCGGGAGCTGCATGTGTCAGAGGTTTNCACCGTCATCACCGAAACGCGCGAGACGAAAGGGNCNTCGTGATACGCCTATTTTAATAGGTTAATGTCATGATAAGAATGGGTTNNTTANNCGTCNNGGGGGNNCTTTTCCGGGGAAATGNNNNNNGGAACCCCNNNTTNGNTTNNTTTTNCTAANNANANTTCAAANANGGAATCNNCNCCNGGNANNNANAAACCCNGGAAAAANGNNTTNNNTNAAANTTGGAAANGGNAAANNNTNNNNNTTNNTTNNCCNTTNCNNNG

>450-12

NNNNNNNNNGCGACGTAAGNTGTCGCTTCTCGCTATCATATCATTCGAGCTCTTTTAGAAGGGGAGCTCACTCAAAGAGAGATAGCAGAGAAATACGGAGTCAGTATCGCACAAATTACCAGAGGATCTAATGCCCTTAAAGGATCAGATCCTCAATTTAAAGAGTTTTTACAAAAAGAGATCTGATCTTCTTTTGTAAAATACAAATAAGATTGAAAGTATTTGTATGCACGCGTTGTTAATGAACAAATATTCTGTTTTAGCAGTTTTGGTACGTAAGTATAGCTGCAGCATGCCATGCAAATCAGCTTTTCAAGCTGATTGCTTCCAAGATATTCAAAAATTCATCCTCTTACAGCGTGCCTGGCTAAAAAAAAAAAAAAAAAAAAAAAAAAAAAAGTACTCTGCGTTGATACCACTGCTTGCCCTATAGTGAGTCGTATTAGGAATTCACTGGCCGTCGTTTTACAACGTCGTGACTGGGAAAACCCTGGCGTTACCCAACTTAATCGCCTTGCAGCACATCCCCCTTTCGCCAGCTGGCGTAATAGCGAAGAGGCCCGCACCGATCGCCCTTCCCAACAGTTGCGCAGCCTGAATGGCGAATGGCGCCTGATGCGGTATTTTCTCCTTACGCATCTGTGCGGTATTTCACACCGCATATGGTGCACTCTCAGTACAATCTGCTCTGATGCCGCATAGTTAAGCCAGCCCCGACACCCGCCAACACCCGCTGACGCGCCCTGACGGGCTTGTCTGCTCCCGGCATCCGCTTACAGACAAGCTGTGACCGTCTCCGGGAGCTGCATGTGTCAGAGGTTTTCACCGTCATCACCGAAACGCGCGAGACGAAAGGGCCTCGTGATACGCCTATTTTTATAGGTTAATGTCATGATAATAATGGTTTCTTAGACGTCAGGTGGCACTTTTCGGGGAAATGTGCGCGGAACCCCTATTTGTTTATTTTTCTAAATACATTCAAATATGTATCCGCTCATGAAACAATAACCCTGATAAATGCTTCAATAATATTGAAAAAGGAANAGTNNGAGTATTCAACATTTCCGGNTCNCCCTTATTCCCTTTTTTGCGGCNTTTNGCCTTCCNGTTTTNNCCCCNNNNAAACCCTGGGGAAANNNAAAANTNCTGAAANNNNTTNGGNNGCCNNNNGGGTTNNNCNAANTGGATTNNNNNNNNGNAANNNNCTTNNNNNNTTTNNCCCNNAAANNNTTTTCCAANNNNNANNCTTTAA

>550-2

NNNNNGNNNNNGTAAGNTGTCGCTTCTCGCTATCATATCATTCGAGCTCTTTTAGAAGGGGAGCTCACTCAAAGAGAGATAGCAGAGAAATACGGAGTCAGTATCGCACAAATTACCAGAGGATCTAATGCCCTTAAAGGATCAGATCCTCAATTTAAAGAGTTTTTACAAAAAGAGATCTGATCTTCTTTTGTAAAATACAAATAAGATTGAAAGTATTTGTATGCACGCGTTGTTAATGAACAAATATTCTGTTTTAGCAGTTTTGGTACGTAAGTATAGCTGCAGCATGCCATGCAAATCAGCTTTTCAAGCTGATTGCTTCCAAGATATTCAAAAATTCATCCTCTTACAGCGTGCCTGGCTTTCTTTTGAAAGCTGGCGCTTATCTACTTGGCGATAGGCCTAATTAAGAAGCCTTTTAAAAAAAAAAAAAAAAAAAAAAAAAAAAAAAAAAAAAAAAAAAAAAAAAATAAAAAAAAAAAAAAAAAAAAAAAAAAAAAAGTACTCTGCGTTGATACCACTGCTTGCCCTATANNNAGTCNNNTTAGGAATTCANGGNCCGTCGTTTTACAACGTCGGGACGGGGAAAACCCTGGCGTTACCCAACTTAATCGCCTTGCAGCACATCCCCCTTTCCCNAGNTGGCGTAATAGCGAAAAGGCCCGCACCGATCGCCCTTCCCAACAGTTGCNNACCCTGAATGGCNAAGGGCGCCTGATGCGGTATTTTCTCCTTACGCATCTGTGCGGTATTTCACACCGCATATGGNGCACTCTCAGTACAATCTGCTCTGATGCCGCATAGTTAANCCAGCCCCGACACCCGCCAACACCCGCTGACGCGCCCTGACGGGCTTGTCTGCTCCCGGCATCCGCTTACAGACAAGCTGTGACCGTCTCCGGGAGCTGCNNNNNNNCAAAGGTTTTCACCGTCATCACCGAAACGCGCGAAANNAAAGGGCCTCNNGANNCGCCTATTTTTNNNGGGTAANGGCNGGAAAANAANGGTTTCTTAAACGTCAGGGGGGNNTTTNCNGGGGAAATGNNNCNGGAACCCNTNTTTNNTTATTTTTNAAAANNNTTCNAANNGNNCNNNNNNNGNAAAAAANNCCCNNNAANNGNNTCNNNNANTTGGAAAAGGNNNNNNTNNANTNCAANTTNCCGGGGNCCCNNNNTNCCTTTTTNGNNANNTTNGCNNNCNGGTTTTGNNNNCCNAAACCNNGGNAAAANAAAANNNNNAAAANNTTNGGG

>550-4

NNNNNNNNNNNANGTAAGATGTCGCTTCTCGCTATCATATCATTCGAGCTCTTTTTAGAAGGGGAGCTCACTCAAAGAGAGATAGCAGAGAAATACGGAGTCAGTATCGCACAAATTACCAGAGGATCTAATGCCCTTAAAGGATCAGATCCTCAATTTAAAGAGTTTTTACAAAAAGAGATCTGATCTTCTTTTGTAAAATACAAATAAGATTGAAAGTATTTGTATGCACGCGTTGTTAATGAACAAATATTCTGTTTTAGCAGTTTTGGTACGTAAGTATAGCTGCAGCATGCCATGCAAATCAGCTTTTCAAGCTGATTGCTTCCAAGATATTCAAAAATTCATCCTCTTACAGCGTGCCTGGCTTTCTTTTGAAAGCTGGCGCTTATCTACTTGGCGATAGGCCTAATTAAGAAGCCTTTTATTTGATTAAGAGATGTTCTTATAGAAGTAAGAGCGTCTTTTTTGCGCAGGATTATTCCAAAAAAAAAAAAAAAAAAAAAAAAAAAAAAAGTACTCTGCGTTGATACCACTGCTTGCCCTATAGTGAGTCGTATTAGGAATTCACTGGCCGTCGTTTTACAACGTCGTGACTGGGAAAACCCTGGCGTTACCCAACTTAATCGCCTTGCAGCACATCCCCCTTTCGCCAGCTGGCGTAATAGCGAAGAGGCCCGCACCGATCGCCCTTCCCAACAGTTGCGCAGCCTGAATGGCGAATGGCGCCTGATGCGGTATTTTCTCCTTACGCATCTGTGCGGTATTTCACACCGCATATGGTGCACTCTCAGTACAATCTGCTCTGATGCCGCATAGTTAAGCCAGCCCCGACACCCGCCAACACCCGCTGACGCGCCCTGACGGGCTTGTCTGCTCCCGGCATCCGCTTACAGACAAGCTGTGACCGTCTCCGGGAGCTGCATGTGTCAAAGGTTTTCACCGTCATCACCGAAACGCGCGAAACGAAAGGGCCTCGTGATACNCCNNTTTTTNNNGGTTAATGNCNANGAANAAAGGGTTTCTTAAANNTCCGGGGGGCACTTTTNGGGNAANNNNNGNNNGGAACCCNNANTTTGTTTAATTTTNCCAANNACATTCAAANNGNTATCCCCNNNNGNAANNNANAACCNNNAAANNNGCTNCAAAAANNTTGAAAAAGGNAANNNNANNANNNNNNNNNNNTTNCCNGNCCCCCNTTTTNCCNTTTTTTNGNGNNATTNNNC

>550-6

NNNNNNNNNNNGANGTNNNATGTCGCTTCTCGCTATCATATCATTCGAGCTCTTTTAGAAGGGGAGCTCACTCAAAGAGAGATAGCAGAGAAATACGGAGTCAGTATCGCACAAATTACCAGAGGATCTAATGCCCTTAAAGGATCAGATCCTCAATTTAAAGAGTTTTTACAAAAAGAGATCTGATCTTCTTTTGTAAAATACAAATAAGATTGAAAGTATTTGTATGCACGCGTTGTTAATGAACAAATATTCTGTTTTAGCAGTTTTGGTACGTAAGTATAGCTGCAGCATGCCATGCAAATCAGCTTTTCAAGCTGATTGCTTCCAAGATATTCAAAAATTCATCCTCTTACAGCGTGCCTGGCTTTCTTTTGAAAGCTGGCGCTTATCTACTTGGCGATAGGCCTAATTAAGAAGCCTTTTATTTGATTAAGAGATGTTCTTATAGAAGTAAGAGCGTCTTTTTTGCGCAGGATTATTCTGTCGCCAGTTTTTTCTGAAAAAAAAAAAAAAAAAAAAAAAAAGTACTCTGCGTTGATACCACTGCTTGCCCTATAGTGAGTCGTATTAGGAATTCACTGGCCGTCGTTTTACAACGTCGTGACTGGGAAAACCCTGGCGTTACCCAACTTAATCGCCTTGCAGCACATCCCCCTTTCGCCAGCTGGCGTAATAGCGAAGAGGCCCGCACCGATCGCCCTTCCCAACAGTTGCGCAGCCTGAATGGCGAATGGCGCCTGATGCGGTATTTTCTCCTTACGCATCTGTGCGGTATTTCACACCGCATATGGTGCACTCTCAGTACAATCTGCTCTGATGCCGCATAGTTAAGCCAGCCCCGACACCCGCCAACACCCGCTGACGCGCCCTGACGGGCTTGTCTGCTCCCGGCATCCGCTTACAGACAAGCTGTGACCGTCTCCGGGAGCTGCATGTGTCAGAGGTTTTCACCGTCNTCACCGAAACGCGCGAGACGAAAGGGCCTCGTGATACGCCTATTTTTAAAGGTTAATGTCATGANAANAAGGGTTTCTTANANGTCAGGNGGNACTTTNCGGGNAANNNNNCNNGGAACCCCTATTTGTTNNNTTTNCCAANNNNTTNCAANNNNNNNCCGNNNNGNANNNANAACCNNNAANNNGNNNNCNNANNNTNGAAAANGGAANNNNNNNNNNTTNNNNNTTCCNGNNGCCCNTNNTNCCCTTTTNGNNNNTTTTNNCCNNCNNT

>550-7

NNNNNNNGNNNANGNNNNATGTCGCTTCTCGCTATCATATCATTCGAGCTCTTTTAGAAGGGGAGCTCACTCAAAGAGAGATAGCAGAGAAATACGGAGTCAGTATCGCACAAATTACCAGAGGATCTAATGCCCTTAAAGGATCAGATCCTCAATTTAAAGAGTTTTTACAAAAAGAGATCTGATCTTCTTTTGTAAAATACAAATAAGATTGAAAGTATTTGTATGCACGCGTTGTTAATGAACAAATATTCTGTTTTAGCAGTTTTGGTACGTAAGTATAGCTGCAGCATGCCATGCAAATCAGCTTTTCAAGCTGATTGCTTCCAAGATATTCAAAAATTCATCCTCTTACAGCGTGCCTGGCTTTCTTTTGAAAGCTGGCGCTTATCTACTTGGCGATAGGCCTAATTAAGAAGCCTTTTATTTGATTAAGAGATGTTCTTATAGAAGTAAGAGCGTCTTTTAAAAAAAACAGAAAAAAAAAAAAAAAAAAAAAAAAAAAAGTACTCTGCGTTGATACCACTGCTTGCCCTATAGTGAGTCGTATTAGGAATTCACTGGCCGTCGTTTTACAACGTCGTGACTGGGAAAACCCTGGCGTTACCCAACTTAATCGCCTTGCAGCACATCCCCCTTTCGCCAGCTGGCGTAATAGCGAAGAGGCCCGCACCGATCGCCCTTCCCAACAGTTGCGCAGCCTGAATGGCGAATGGCGCCTGATGCGGTATTTTCTCCTTACGCATCTGTGCGGTATTTCACACCGCATATGGTGCACTCTCAGTACAATCTGCTCTGATGCCGCATAGTTAAGCCAGCCCCGACACCCGCCAACACCCGCTGACGCGCCCTGACGGGCTTGTCTGCTCCCGGCATCCGCTTACAGACAAGCTGTGACCGTCTCCGGGAGCTGCATGTGTCAGAGGTTTTCACCGTCATCACCGAAACGCGCGAGACGAAAGGGCCTCGTGATACGCCTATTTTTNNAGGTTAATGNCATGANAAANAATGGTTTCTTAGACGTCAGGNGGCACTTTTCGGGGAAATNNNNCNGGAACCCCTATTNGGTTATTTTTCNAAATNNNTTCAANNNGNTATCCNNNCNNNANNNANTAACCNNGGNNAANGNCTTCANNAAANTTGAAAAAGGAANGNTTGGNNTNTNNNNNATTTCCGGGNCCCCNTNNTNCCTTTTTNGGNNNATTNGNCNTTCCNNTTTNNNCCCNNNAAAANCNNNGGNAANNNAAAAAGGCGNNAANNCTTGGGGNNCNNNGGGGTTNNCNNNNNGG

>550-8

NNNNNNNNNNNNNGTNNNATGTCGCTTCTCGCTATCATATCATTCGAGCTCTTTTAGAAGGGGAGCTCACTCAAAGAGAGATAGCAGAGAAATACGGAGTCAGTATCGCACAAATTACCAGAGGATCTAATGCCCTTAAAGGATCAGATCCTCAATTTAAAGAGTTTTTACAAAAAGAGATCTGATCTTCTTTTGTAAAATACAAATAAGATTGAAAGTATTTGTATGCACGCGTTGTTAATGAACAAATATTCTGTTTTAGCAGTTTTGGTACGTAAGTATAGCTGCAGCATGCCATGCAAATCAGCTTTTCAAGCTGATTGCTTCCAAGATATTCAAAAATTCATCCTCTTACAGCGTGCCTGGCTTTCTTTTGAAAGCTGGCGCTTATCTACTTGGCGATAGGCCTAATTAAGAAGCCTTTTATTTGATTAAGAGATGTTCTTATAGAAGTAAGAGCGTCTTTTTTGCGCAGGATTATTCCAAAAAAAAAAAAAAAAAAAAAAAAAAAAGTACTCTGCGTTGATACCACTGCTTGCCCTATAGTGAGTCGTATTAGGAATTCACTGGCCGTCGTTTTACAACGTCGTGACTGGGAAAACCCTGGCGTTACCCAACTTAATCGCCTTGCAGCACATCCCCCTTTCGCCAGCTGGCGTAATAGCGAAGAGGCCCGCACCGATCGCCCTTCCCAACAGTTGCGCAGCCTGAATGGCGAATGGCGCCTGATGCGGTATTTTCTCCTTACGCATCTGTGCGGTATTTCACACCGCATATGGTGCACTCTCAGTACAATCTGCTCTGATGCCGCATAGTTAAGCCAGCCCCGACACCCGCCAACACCCGCTGACGCGCCCTGACGGGCTTGTCTGCTCCCGGCATCCGCTTACAGACAAGCTGTGACCGTCTCCGGGAGCTGCATGTGTCAAAGGTTTTCACCGTCATCACCGAAACGCGCGAGANNAAAGGGCCTCGNNATACGCCTATTTTTAANGGGTAAAGGCCAGGGANAAAANGGGTTTCTTANANNNNNGGNGGCCNNTTTTCGGGGAAATGNGCNNGGAACCCCNTNNTTGNTTATTTTTNNAAANNCTTNCAAANNNNNNNCNNNNNNGGANNNANNANCCCNGNNAANNGGCNNCCAAAANTTGAAAANGGAANNNTTNNANNTTNNNNNNTTCCCGGGNCCCCNTNATTCCTTTNNTTGNNNNANTTGGCNNNNNGGNTTTNGGCCCCCNNAAAAACCNGNGGNA

>550-12

NNNNNNNCNNNGNNNNATGTCGCTTCTCGCTATCATATCATTCGAGCTCTTTTAGAAGGGGAGCTCACTCAAAGAGAGATAGCAGAGAAATACGGAGTCAGTATCGCACAAATTACCAGAGGATCTAATGCCCTTAAAGGATCAGATCCTCAATTTAAAGAGTTTTTACAAAAAGAGATCTGATCTTCTTTTGTAAAATACAAATAAGATTGAAAGTATTTGTATGCACGCGTTGTTAATGAACAAATATTCTGTTTTAGCAGTTTTGGTACGTAAGTATAGCTGCAGCATGCCATGCAAATCAGCTTTTCAAGCTGATTGCTTCCAAGATATTCAAAAATTCATCCTCTTACAGCGTGCCTGGCTTTCTTTTGAAAGCTGGCGCTTATCTACTTGGCGATAGGCCTAATTAAGAAGCCTTTTATTTGATTAAGAGATGTTCTTATAGAAGTAAGAGCGTCTTTTTTACTAAAAAAAAAAAAAAAAAAAAAAAAAAAAAAAGTACTCTGCGTTGATACCACTGCTTGCCCTATAGTGAGTCGTATTAGGAATTCACTGGCCGTCGTTTTACAACGTCGTGACTGGGAAAACCCTGGCGTTACCCAACTTAATCGCCTTGCAGCACATCCCCCTTTCGCCAGCTGGCGTAATAGCGAAGAGGCCCGCACCGATCGCCCTTCCCAACAGTTGCGCAGCCTGAATGGCGAATGGCGCCTGATGCGGTATTTTCTCCTTACGCATCTGTGCGGTATTTCACACCGCATATGGTGCACTCTCAGTACAATCTGCTCTGATGCCGCATAGTTAAGCCAGCCCCGACACCCGCCAACACCCGCTGACGCGCCCTGACGGGCTTGTCTGCTCCCGGCATCCGCTTACAGACAAGCTGTGACCGTCTCCGGGANCTGCATGTGGTCAAAGGTTTTCACCGTCATCACCGAAACGCCNNAAANCAAAAGGGCCTCGGGATACGCCTATTTTTAAAGGGTAAGNNCNGGAAAANAAAGGGTTTCTTAAANNNCNNGGGGGCACTTTTNNGGGGAAANGGGNNNGGNAACCCNNNNTNNNTTNNTTTTNCNAAAAANNNTTCCAAANNGGNNCCCCCNTNNNNAANCAAAANCCCNGNAAAAAGGNNNNNNNNNNTTNNGGAAAG
