# Supplementary material for: A bipartite iron-dependent transcriptional regulation of the tryptophan salvage pathway in Chlamydia trachomatis
Source: eLife. 2019 Apr 2;8:e42295. doi: 10.7554/eLife.42295 (PMC6504234; doi:10.7554/eLife.42295)
Supplement: Transparent reporting form [file elife-42295-transrepform.pdf]

eLIFE
1st Floor
24 Hills Road
Cambridge CB2 1JP, UK
P 01223 855340
W elifesciences.org
T @elife

eLife's transparent reporting form

We encourage authors to provide detailed information *within their submission* to facilitate the interpretation and replication of experiments. Authors can upload supporting documentation to indicate the use of appropriate reporting guidelines for health-related research (see EQUATOR Network), life science research (see the BioSharing Information Resource), or the ARRIVE guidelines for reporting work involving animal research. Where applicable, authors should refer to any relevant reporting standards documents in this form.

If you have any questions, please consult our Journal Policies and/or contact us: editorial@elifesciences.org.

Sample-size estimation

- You should state whether an appropriate sample size was computed when the study was being designed
- You should state the statistical method of sample size computation and any required assumptions
- If no explicit power analysis was used, you should describe how you decided what sample (replicate) size (number) to use

Please outline where this information can be found within the submission (e.g., sections or figure legends), or explain why this information doesn't apply to your submission:

Information on sample sizes can be found in the respective figure legends. No explicit power analysis was used to compute sample size. Unless otherwise stated, three biological replicates were collected for each quantitative experiment.

Replicates

- You should report how often each experiment was performed
- You should include a definition of biological versus technical replication
- The data obtained should be provided and sufficient information should be provided to indicate the number of independent biological and/or technical replicates
- If you encountered any outliers, you should describe how these were handled
- Criteria for exclusion/inclusion of data should be clearly stated
- High-throughput sequence data should be uploaded before submission, with a private link for reviewers provided (these are available from both GEO and ArrayExpress)

Please outline where this information can be found within the submission (e.g., sections or figure legends), or explain why this information doesn't apply to your submission:

Information of replicates collected and considered for analysis can be found in respective methods sections for each experiment. Technical replication was considered to be any replication that occurred at the level of sample collection or analysis of a single experiment (*e.g.* replicate assays on sample collected from given day) and biological replication was considered to be any replication between independent experiments (*e.g.* replicate samples collected and processed independently).

eLife Sciences Publications, Ltd is a limited liability non-profit non-stock corporation incorporated in the State of Delaware, USA, with company number 5030732, and is registered in the UK with company number FC030576 and branch number BR015634 at the address 1st Floor, 24 Hills Road, Cambridge CB2 1JP | August 2014

Statistical reporting

- Statistical analysis methods should be described and justified
- Raw data should be presented in figures whenever informative to do so (typically when N per group is less than 10)
- For each experiment, you should identify the statistical tests used, exact values of N, definitions of center, methods of multiple test correction, and dispersion and precision measures (e.g., mean, median, SD, SEM, confidence intervals; and, for the major substantive results, a measure of effect size (e.g., Pearson's r, Cohen's d)
- Report exact p-values wherever possible alongside the summary statistics and 95% confidence intervals. These should be reported for all key questions and not only when the p-value is less than 0.05.

Please outline where this information can be found within the submission (e.g., sections or figure legends), or explain why this information doesn't apply to your submission:

Information of statistical analysis can be found in the respective figure legend of a given experiment and also in the materials and methods section. Exact P-values are reported in text for all critical experimental questions.

(For large datasets, or papers with a very large number of statistical tests, you may upload a single table file with tests, Ns, etc., with reference to sections in the manuscript.)

Group allocation

- Indicate how samples were allocated into experimental groups (in the case of clinical studies, please specify allocation to treatment method); if randomization was used, please also state if restricted randomization was applied
- Indicate if masking was used during group allocation, data collection and/or data analysis

Please outline where this information can be found within the submission (e.g., sections or figure legends), or explain why this information doesn't apply to your submission:

Group allocation was based on treatment or experimental condition in all cases, and therefore there is no specific description of the methods used to determine experimental groups.

Additional data files ("source data")

- We encourage you to upload relevant additional data files, such as numerical data that are represented as a graph in a figure, or as a summary table
- Where provided, these should be in the most useful format, and they can be uploaded as "Source data" files linked to a main figure or table
- Include model definition files including the full list of parameters used
- Include code used for data analysis (e.g., R, MatLab)
- Avoid stating that data files are "available upon request"

Please indicate the figures or tables for which source data files have been provided:

eLIFE
1st Floor
24 Hills Road
Cambridge CB2 1JP, UK
P 01223 855340
W elifesciences.org
T @elife

Source data for Figure 1 qPCR and RT-qPCR experiments have been provided
Source data for Figure 2 RT-qPCR experiments have been provided.
Source data for Figure 3 RT-qPCR experiments have been provided.
Source data for Figure 4B RT-qPCR experiment has been provided.
Raw sequencing data for Figure 4C has been provided in Supplementary File 3.
Source data has been provided for Figure 4C and Figure 6C, in supplementary files 1 and 2, respectively. These files also include descriptive statistical computations to allow the reader to unbiasedly assess the datasets.
Source data for Figure 5 Beta-galactosidase reporter experiments have been provided.
Source data for the RNA-Sequencing in Figure 6A can be found at GEO accession number GSE106763.
Raw sequencing data for Figure 6C has been provided in Supplementary File 4.
Source data for Figures 7C-E RT-qPCR experiments have been provided.
Example of source code used for generating the histogram and density plot figures for Figure 4C and 6C is provided in Source Code File 1.
Example of source code for One-Way ANOVA and post-hoc pairwise t-tests is provided in Source Code File 2.
Example of source code for Student's t-test with Welch's correction for unequal variance is provided in Source Code File 3.

eLife Sciences Publications, Ltd is a limited liability non-profit non-stock corporation incorporated in the State of Delaware, USA, with company number 5030732, and is registered in the UK with company number FC030576 and branch number BR015634 at the address 1st Floor, 24 Hills Road, Cambridge CB2 1JP | August 2014
